# Proteomic analysis reveals changes in the proteome of human THP-1 macrophages infected with *Paracoccidioides brasiliensis*

**DOI:** 10.3389/fcimb.2023.1275954

**Published:** 2023-11-16

**Authors:** Ana Marina Barroso de Figueiredo, Dayane Moraes, Alexandre Melo Bailão, Olivia Basso Rocha, Lana Ohara Souza Silva, Fátima Ribeiro-Dias, Célia Maria de Almeida Soares

**Affiliations:** ^1^ Laboratório de Imunidade Natural (LIN), Instituto de Patologia Tropical e Saúde Pública, Universidade Federal de Goiás, Goiânia, Goiás, Brazil; ^2^ Laboratório de Biologia Molecular, Instituto de Ciências Biológicas, Universidade Federal de Goiás, Goiânia, Goiás, Brazil

**Keywords:** *Paracoccidioides brasiliensis*, proteomic analysis, metabolic reprogramming, immunometabolism, macrophages

## Abstract

*Paracoccidioides* spp. is the etiologic agent of Paracoccidioidomycosis (PCM), a systemic disease with wide distribution in Latin America. Macrophages are very important cells during the response to infection by *P. brasiliensis*. In this study, we performed a proteomic analysis to evaluate the consequences of *P. brasiliensis* yeast cells on the human THP-1 macrophage proteome. We have identified 443 and 2247 upregulated or downregulated proteins, respectively, in macrophages co-cultured with yeast cells of *P. brasiliensis* in comparison to control macrophages unexposed to the fungus. Proteomic analysis revealed that interaction with *P. brasiliensis* caused metabolic changes in macrophages that drastically affected energy production pathways. In addition, these macrophages presented regulated many factors related to epigenetic modifications and gene transcription as well as a decrease of many proteins associated to the immune system activity. This is the first human macrophage proteome derived from interactions with *P. brasiliensis*, which contributes to elucidating the changes that occur during the host response to this fungus. Furthermore, it highlights proteins that may be targets for the development of new therapeutic approaches to PCM.

## 1 Introduction

Paracoccidioidomycosis (PCM) is a systemic mycosis widely distributed in Latin America; Brazil is responsible for the largest number of reported cases. The etiological agent of PCM consists of thermodimorphic fungus of the genus *Paracoccidioides*, which presents the mycelium form in the soil (25°C) and the yeast form in the host (37°C). *Paracoccidioides brasiliensis* has a wide distribution in Latin America and is the most studied species. PCM affects mainly rural workers, and infection occurs by inhalation of the infectious propagules present in the soil that, when reaching the lungs, differentiate into yeast cells. The lung is the main organ affected during the disease; however, yeast cells can proliferate in the lung and spread through the host organism affecting organs such as lymph nodes, liver, and skin (Shikanai-Yasuda et al., 2006; Mendes et al., 2017; Chaves et al., 2021).

Individuals infected with *Paracoccidioides* spp. may present different clinical manifestations depending on both pathogen and host factors. The infection mainly affects children, adolescents, and young adults, who usually develop a T helper (Th) lymphocyte response with a Th2/Th9 profile. This profile is associated with susceptibility and characterizes the acute form of PCM. On the other hand, the chronic form of the disease is characterized by a slow progression and predominantly affects adults over 30 years of age, who usually develop a Th17/Th22 type response, and a substantial participation of Th1 lymphocytes. This mixed immune response is crucial for infection control. Asymptomatic individuals usually develop a Th1-type immune response, which can contain the spread of the fungus and prevent the progression of the disease (Oliveira et al., 2002; De Castro et al., 2013; Martinez, 2017).

The innate immune mechanisms responsible for pathogen elimination are induced by pathogen-associated molecular patterns (PAMPs) of the fungus that activate pattern-recognizing receptors (PRRs) on the host cells. Therefore, fungus cell wall composition is a critical factor for the pathogen-host interaction and, consequently, the outcome of the infection (Gow et al., 2017). The hypha to yeast transition of *Paracoccidioides* spp. involves changes in the composition and structure of the cell wall, which are necessary for the establishment of the infection. Yeast cells present a thicker cell wall as well as a higher amount of chitin and α-glucans than hyphae (Chaves et al., 2021). As β-glucan is a relevant PAMP to activate the innate immune response against *Paracoccidioides* spp. (Loures et al., 2014), the masking of β-glucan by α-glucan is considered an immune evasion mechanism (Puccia et al., 2011). In addition, *P. brasiliensis* has other virulence factors such as proteases and adhesins, which facilitate the invasion and colonization of tissue by the fungus (Vicentini et al., 1994; Hernández et al., 2010; Bailão et al., 2012; Lacerda Pigosso et al., 2017).

Macrophage are a crucial cell in the immune response against fungal infections. The recognition of fungal PAMPs and/or activation by cytokines triggers macrophage microbicidal mechanisms. These responses include the production of reactive oxygen (ROS) or nitrogen (RNS) species, which are responsible by the killing of the pathogen (Bogdan et al., 2000). However, even though living inside macrophages is a big challenge *Paracoccidioides* spp. may evade of the immune response and multiply inside these cells (Brown et al., 2009). Therefore, efficient infection control depends on how macrophages are activated. As described above, type 1 cytokines, such as interferon-gamma (IFNγ) and tumor necrosis factor (TNF), activate macrophages, contributing to a pro-inflammatory phenotype and increased microbicidal activity. On the other hand, in the presence of type 2 or regulatory cytokines (IL-4, IL-13, IL-10) macrophages are alternatively activated, showing an anti-inflammatory profile, and contribute to tissue repair (Ferrante and Leibovich, 2012). Recently, our group demonstrated that murine alveolar macrophages activated with IFNγ are less permissive to *P. brasiliensis* survival and proliferation (Chaves et al., 2019). Other studies have also demonstrated its relevance for *P. brasiliensis* infection control (Brummer et al., 1988; Brummer et al., 1989; Moscardi-Bacchi et al., 1994; Gonzalez et al., 2000).

It has been shown that during the infections, the host cells and fungi can undergo reprogramming in their gene expression and metabolism. The fungus changes to establish infection and evade the immune system while the host cell adopts mechanisms to eradicate the infection (Silva et al., 2008; De Arruda Grossklaus et al., 2013; Parente-Rocha et al., 2015; Lacerda Pigosso et al., 2017; Weerasinghe and Traven, 2020). Our group has studied the proteome of *Paracoccidioides* spp. after interaction with macrophages. Members of the *Paracoccidioides* complex have been observed to respond to host cell effector mechanisms through an oxidative stress response characterized by increase of several antioxidant enzymes and virulence factors. In addition, fungus presents metabolic reprogramming changing from glycolysis to gluconeogenesis, beta-oxidation, and catabolism of amino acids (De Arruda Grossklaus et al., 2013; Parente-Rocha et al., 2015; Lacerda Pigosso et al., 2017; Chaves et al., 2019). Despite these descriptions, so far there is only one study that performed a proteomic analysis focusing on the host response to fungal infection whose objective was to identify serum biomarkers of severe pulmonary sequelae and relevant signaling pathways in different clinical stages of patients with PCM (Santos et al., 2021). Santos et al. (2021) have identified 72 proteins differentially present in the serum of patients with severe sequelae compared to the serum of patients with mild/moderate sequelae. In addition, the study showed that the development of severe sequelae is associated with an increase in glycolytic pathway proteins and proteins involved in oxygen exchange, suggesting high cellular activity and deregulation of wound healing. Understanding the host-pathogen interface is an important tool that can help the development of new therapeutic strategies for diseases. THP-1 cells, a human leukemia monocytic cell line, have been one of the widely used models for studies on monocytes and macrophages. Among several advantages, THP-1 cells are easily derived into macrophages and are advantageous for studies that require a large number of cells. Furthermore, these cells can be stored for several years, and as they are immortalized, they can be cultured for a long time (Chanput et al., 2014). Thus, THP-1 macrophages are a good experimental *in vitro* model to study the human host response to *P. brasiliensis* infection. The present study aimed to evaluate the effect of *P. brasiliensis* on the THP-1 macrophage proteome, highlighting the proteins from the host cell are produced in response to the first 24 h of this interaction.

## 2 Materials and methods

### 2.1 Culture of *Paracoccidioides brasiliensis* yeast cells

Yeast cells of *P. brasiliensis* (*Pb*18 - ATCC 32069) were grown in complete solid Fava-Netto medium [1% (w/v) peptone (Kasvi, São José dos Pinhais, PR, Brazil), 0.5% (w/w) yeast extract (BD, Franklin Lakes, NJ, USA), 0.3% (w/v) proteose peptone (Sigma-Aldrich, Saint Louis, MO, USA), 0.5% (w/v) meat extract (BD), 0.5% (w/v) NaCl (Sigma-Aldrich), 4% (w/w) glucose (Sigma-Aldrich), 1% (w/v) brain and heart infusion (BD) and 1.2% (w/v) agar (BD), pH 7.2]. The medium was supplemented with 5% (v/v) fetal bovine serum (FBS, Cripion, Andradina, SP, Brazil) and 10 μg/mL gentamicin (Sigma-Aldrich). The culture was maintained at 36°C and sub cultivations were made every seven days (Bastos et al., 2007). To obtain yeast cells of *P. brasiliensis*, fungi were cultured in brain and heart infusion (BD) medium, supplemented with 4% (w/w) glucose, 5% (v/v) FBS, and 10 μg/mL gentamicin (Complete BHI medium). The culture was maintained at 36 °C during three days under agitation, centrifuged (1,400 ɡ, 5 min), washed three times with phosphate-buffered saline solution (PBS), and the supernatant was discarded. Yeast cell quantification was performed on a hematocytometer, and vital staining with 0.1% (w/v) trypan blue in PBS assessed cell viability.

### 2.2 Obtaining THP-1 macrophage and interaction assays

**Figure 1:**
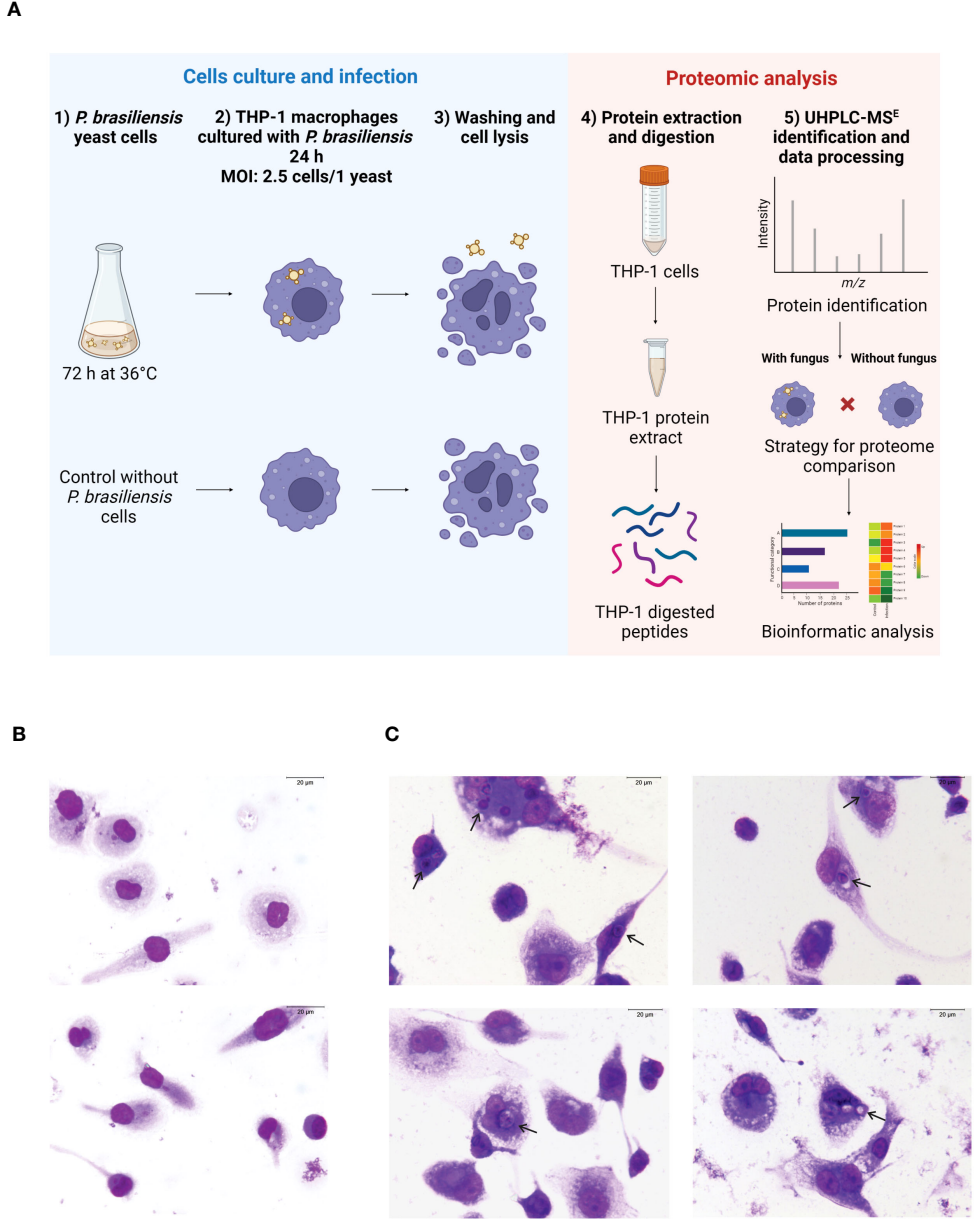
THP-1 macrophages and *Paracoccidioides brasiliensis* yeast cells were co-cultured for 24 h, and proteomic analysis was done by UHPL-MSE. **(A)** Flowchart of the methodology (Created with BioRender.com). **(B)** Photomicrographs of macrophages in the absence of fungus. **(C)** Photomicrographs of macrophages co-cultured with the fungi. Arrows indicate cells with internalized fungus (400 times magnification). Scale bar is 20 μm. Cells were stained with Giemsa.

The human monocyte cell line THP-1 was cultured in RPMI 1640 medium (Sigma), supplemented with 10% inactivated FBS (Gibco), 2 mM L-glutamine (Sigma), 11 mM sodium bicarbonate, 100 U/mL of penicillin (Sigma), and 100 µg/mL of streptomycin (Sigma), referred as complete medium. Cells were maintained in a humidified atmosphere with at 5% CO_2_, at 37°C. To obtain macrophages, THP-1 cells were cultivated in petri dishes for cell culture (TPP, Switzerland) (10^6^ cells/mL) and incubated with 100 ng/mL of phorbol myristate acetate (PMA) for 48 h. After incubation, the cells were washed 5x with PBS at 37°C. After washing, the complete medium was replaced, and the cells were re-incubated for 24 h (Lund et al., 2016). Then, the THP-1 macrophages were incubated with yeast cells of *P. brasiliensis* (MOI: 2.5 cells/1 yeast) for 24 h. To obtain photomicrographs of THP-1 macrophages co-cultured with the fungi, glass coverslips (Knittel Glass, SP, São Paulo) were added to a 24-well plate (Corning-Costar - Kennebunk, ME, USA), and the same experimental procedure for cell derivation and macrophage co-cultured with the fungus was performed as described above. Coverslips were harvested after 24 h, and cells were fixed with absolute methanol, and stained with Giemsa (Merck Millipore, Damstadt, Germany). Photomicrographs were taken under a light microscope (400x magnification). The percentage of infected cells and the mean of fungi per cell were obtained by counting a total of 300 cells per coverslips (experimental triplicate). The infection index was calculated as % x mean of fungi per cell. A schematic presentation of the cell and fungus interaction conditions is presented in **Figure 1A**.

### 2.3 Preparation and digestion of protein extracts

After 24h infection, the macrophages were rinsed to remove the unphagocytosed yeast cells. THP-1 macrophages co-cultured with *P. brasiliensis* or not (control macrophages) were removed from the plates with ice-cold PBS using a cell scraper (Corning), washed twice by centrifugation (600 g for 10 min, at 10 °C) and the pellet was resuspended in ammonium bicarbonate buffer (NH_4_HCO_3_) 50 mM, pH 8.5. Then, the cells were centrifuged at 600 g for 10 min at 4°C, and the supernatant was discarded; NH_4_HCO_3_ 50 mM, pH 8.5, and glass beads were added to the pellet. Cells were mechanically lysed in bead beater equipment (BioSpec Products Inc., Bartlesville, OK, USA) for 5 cycles of 30 s. The lysate was centrifuged three times (10,000 ɡ, 10 min, at 4°C); for protein quantification, the Bradford reagent (Sigma Aldrich) was used. The enzymatic digestion of proteins was performed based on Murad et al. (2011). In brief, to microcentrifuge tubes (Axygen, Tewksbury, MA) were added 150 μg of protein sample, 10 μL of NH_4_HCO_3_ at 50 mM, pH 8.5 and 75 μL of RapiGest SF solution (0.2% v/v) (Waters, Milford, MA, USA). The samples were vortexed and incubated in a thermoblock at 80°C for 15 min. To reduce disulfide bonds, 2.5 μL of 100 mM dithiothreitol (DTT) (GE Healthcare, Little Chalfont, UK) was added and the tubes were incubated at 60°C for 30 min. Then, for cysteine ​​alkylation, 2.5 μL of 300 mM iodocetamide (GE Healthcare, Piscataway, NJ, USA) was added, followed by vortexing and incubation at room temperature in the dark, for 30 min. Proteins were digested with 30 μL of trypsin solution (Promega, Madison, WI, USA) 0.05 mg/mL in 50 mM ammonium bicarbonate, then vortexed and incubated in a thermoblock at 37°C for 16 h. Stopping trypsin digestion and RapiGEST precipitation were performed by acidifying samples with 30 μL of 5% (v/v) trifluoroacetic acid (TFA) (Sigma-Aldrich). The samples were vortexed, incubated in a thermoblock at 37°C for 90 min and centrifuged at 18,000 ɡ, at 4°C for 30 min. Then supernatant was transferred to another tube and dried in a speed vacuum device (Eppendorf, Hamburg, Germany). The peptides were resuspended in 80 μL of ammonium formate (20 mM NH_4_HCO_3_, pH 10.0).

### 2.4 Data processing, protein identification and bioinformatics analysis

We used the ACQUITY UPLC^®^ M-Class system (Waters Corporation, Manchester, UK) coupled to Synapt G1 HDMS™ mass spectrometer (Waters Micromass, Manchester, UK) to perform Ultra High-Performance Liquid Chromatography (Baeza et al., 2017). *Oryctolagus cuniculus* glycogen phosphorylase_muscle form (MassPREP™ Digestion Standard) was used as internal standard (200 fmol/mL.) and [GLU1]-Fibrinopeptide B (GFB) for calibration. ProteinLynx Global Server software (PLGS) version 3.0.2 (Waters, Manchester, UK) loaded with a specific database for *Homo sapiens* was used to process the mass spectra according to Geromanos et al. (2009). So, the protein IDs were obtained by using a one-randomized *Homo sapiens* databank in the PLGS software. In summary, the PLGS software uses fourteen parameters to identify a protein in the provided database. According to Moraes et al. (2023), the following protein identification parameters were used: the detection of at least two fragment ions per peptide; five fragment per protein; the determination of at least one unique peptide per protein; carbamidomethylation of cysteine as a fixed modification; variable modifications were considered to be serine, threonine and tyrosine phosphorylations and methionine oxidation. In addition, a maximum protein mass (600 kDa); a lost cleavage site was allowed for trypsin; and 4% of the maximum false positive ratio were considered. Proteins identified with differences in abundance of minimum fold-change of 50% were considered as regulated, that is, fold-change ≤ 0.67 (ratio 1/1.5) to downregulated proteins and fold change ≥ 1.5 (ratio 1.5/1) to upregulated proteins). The PLGS score, the result of different mathematical-statistical models for predicting the assignment of peptides and fragments, was presented for each protein. The number of protein identifications in the three replicates can be verified in column B of **Supplementary Spreadsheet 1**, with the number one indicating that the protein was identified in two replicates and the number two indicating that it was identified in three replicates. Proteins identified in only one replicate were excluded from the analysis. To classify and separate identified proteins according to their function in different biological pathways were used online tools: Kyoto Encyclopedia of Genes and Genomes (KEGG) (https://www.genome.jp/kegg/), UniProt database (https://www.uniprot.org/), NCBI database (https://www.ncbi.nlm.nih.gov/) and CORUM database (http://mips.helmholtz-muenchen.de/corum/#browse). The proteomic data have been deposited to the ProteomeXchange Consortium via the PRIDE (Perez-Riverol et al., 2022; Deutsch et al., 2023), partner repository with the dataset identifier PXD043727.

### 2.5 Assessment of macrophage mitochondrial activity

To verify whether *P. brasiliensis* yeasts altered the mitochondrial membrane integrity and mitochondrial activity of THP-1 macrophages co-cultured or not with the fungi, cells were stained with Rhodamine 123 (Sigma-Aldrich) and MitoTracker Green FM (Sigma-Aldrich). After 24 h of interaction with the fungi, macrophages were collected and simultaneously stained with Rhodamine 123 (2.4 μM) and MitoTracker Green FM (400 μM) and incubated for 45 min in the dark, at room temperature. Cells were centrifuged and washed three times with PBS. Fluorescence was analyzed using a fluorescence microscope (Zeiss Axiocam MRc-Scope A1) using a wavelength of 515–575 nm for Rhodamine and 450–490 nm for MitoTracker Green. The images were captured at 400x magnification. The AxioVision software (Carl Zeiss) evaluated the pixel density. The quantification of pixels was performed in triplicate and all macrophage cells that showed fluorescence, delimited borders and without overlapping were evaluated (according to Rocha et al. (2022). Data represent the mean and standard deviation of three biological replicates. The log of 2.7 was used to normalize the data for statistical analyses. For statistical analysis, the Student’s t test was used, and the established significance level was p ≤ 0.05.

### 2.6 Evaluation of the production of reactive oxygen species by THP-1 macrophages

To evaluate the production of ROS by THP-1 macrophages co-cultured or not with fungi, cells were stained with 2’,7’-dichlorodihydrofluorescein diacetate (DCFH-DA) (Sigma-Aldrich) at 25 µM in the dark, for 25 min. After this period, the macrophages were washed three times with PBS and analyzed in a fluorescence microscope (Axiocam MRc-Scope A1) using the 450-490 nm filter. Pixel density was determined using the AxioVision software (Carl Zeiss). The images were captured at 400x magnification. The quantification of pixels was performed in triplicate and all macrophage cells that showed fluorescence, delimited borders and without overlapping were evaluated. Data represent the mean and standard deviation of three biological replicates. The log of 2.7 was used to normalize the data for statistical analyses. Data was analyzed by Student’s t test and the established significance level was p ≤ 0.05.

## 3 Results

### 3.1 *P. brasiliensis* infects THP-1 macrophages

A flowchart of the methodology is shown in **Figure 1A**. Macrophages derived from THP-1 monocytes were obtained as described in Section 2.2. To investigate the effect of *P. brasiliensis* on THP-1 macrophage proteome, these cells were incubated in the absence (**Figure 1B**) or presence of yeast cells of *P. brasiliensis* for 24 h (**Figure 1C**). This incubation time was selected based on experiments with THP-1-derived macrophages, showing 24 has adequate time to observe production of cytokines, enzymes such as inducible nitric oxide synthase (iNOS) and other proteins following activation by microorganisms or PAMPs (He et al., 2012; Meijer et al., 2015; Dos Santos et al., 2017). Macrophages were efficiently infected by the fungi, as depicted in **Figure 1C**. After 24 h of co-cultures, the percentage of infected macrophages was 24% with a mean of 1.5 yeasts per cell, leading to an infection index 36.

### 3.2 Differentially regulated proteins in TH1-P macrophages co-cultured with *P. brasiliensis*

**Figure 2:**
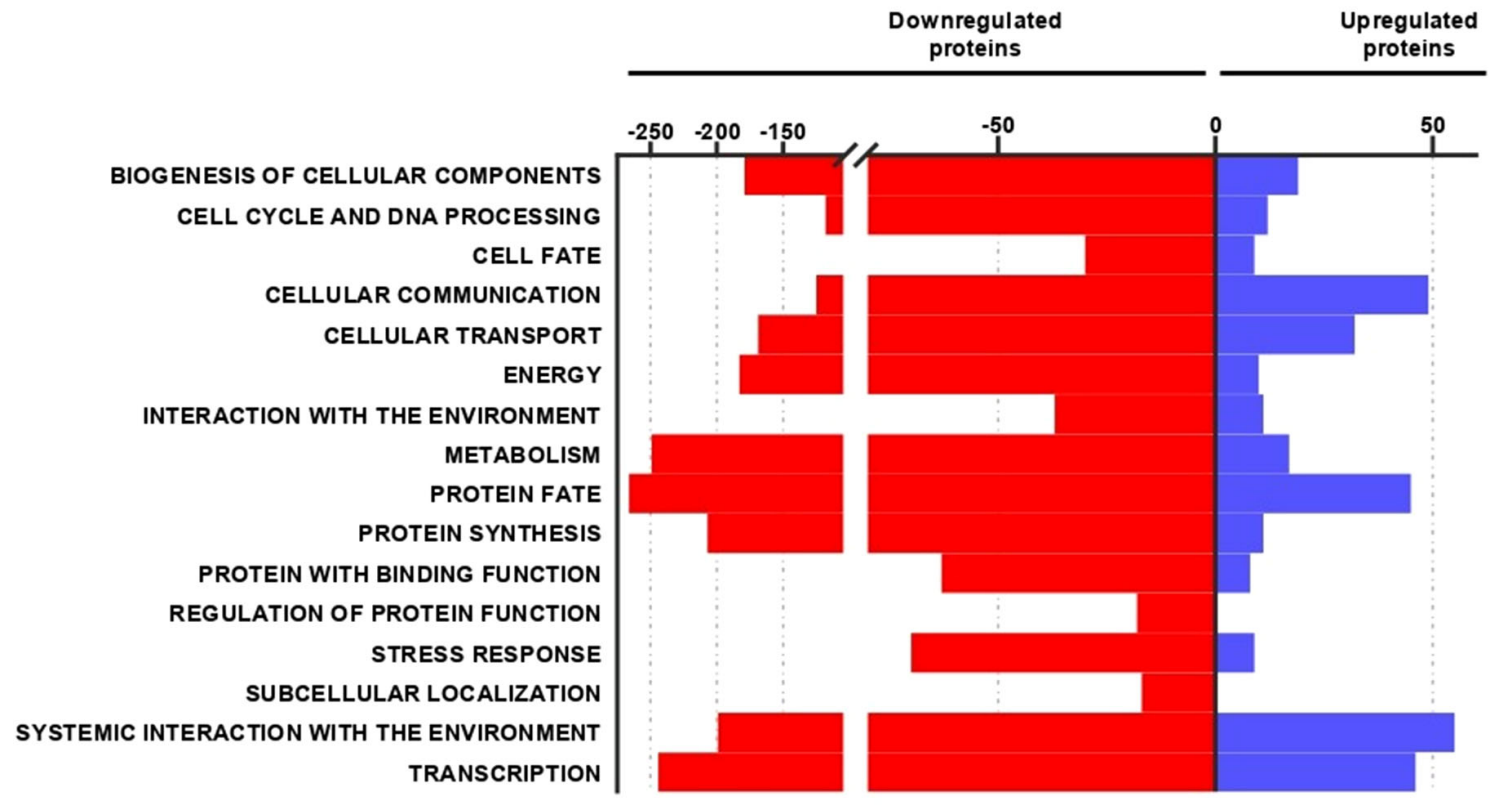
Functional classification of decreased and increased proteins in macrophages co-cultured with *Paracoccidioides brasiliensis*. The top part of the graph shows the number of proteins regulated in each biological process. Red bars and blue bars correspond to the number of proteins repressed or increased, respectively.

To study the effects of *P. brasiliensis* on human macrophage proteomes, these host cells were cultured in the absence or presence of the fungal yeasts. In this large-scale analysis, macrophages were collected at 24 h of incubation. Samples were analyzed in biological triplicates and mass spectrometry analysis provided 2,905 identified proteins (**Supplementary Spreadsheet 1**). Of all proteins, 65% were identified in three replicates and 35% were identified in two replicates. Considering a 1.5-fold change value, the proteomic analysis identified 2,690 differentially expressed proteins (**Supplementary Spreadsheet 1**), among them 2,247 (83.53%) proteins decreased (**Supplementary Table 1**) and 443 (16.47%) proteins increased (**Supplementary Table 2**). These proteins are organized in different functional categories so that we can synthesize information and evaluate the main differences between the protein profile of macrophages cultured without or with *P. brasiliensis* yeasts. The regulated proteins are related to various biological processes, as shown by **Figure 2**. Down-regulated proteins are mainly related to: protein fate (12%), metabolism (11%), transcription (11%), protein synthesis (10%), systemic interaction with the environment, mainly immune response (9%), and energy (8%). Most of *P. brasiliensis*-induced proteins in macrophages play a role in systemic interaction with the environment (16%), cellular communication (15%), transcription (14%), protein fate (13%) and cellular transport (10%). These functional categories are subdivided into smaller groups, for example: protein fate (folding, modification and destination); metabolism (carbohydrates, proteins, lipids, vitamins, etc.); transcription (RNA synthesis, and RNA processing); energy (glycolysis and gluconeogenesis, tricarboxylic acid cycle (TCA), electron transport and membrane-associated energy conservation, etc.).

### 3.3 *P. brasiliensis* induces changes in THP-1 macrophages decreasing enzymes of energy metabolic processes and affecting mitochondrial activity

*P. brasiliensis* decreased proteins involved in macrophage energy production, impairing glycolysis, the TCA and oxidative phosphorylation (**Tables 1**, **2**). Noteworthy, 24 enzymes of glycolysis, including their isoforms, were decreased in macrophages co-cultured with the fungus as depicted in **Table 1**. In addition, the isoforms of lactate dehydrogenase and alcohol dehydrogenase were decreased in macrophages exposed to *P. brasiliensis* indicating an inhibition of fermentation. Also, the downregulation of glycogen phosphorylase suggests a reduction in glycogen degradation, consequently leading to decreased glucose consumption by macrophages (**Table 1**). Additionally, 14 TCA enzymes and 12 proteins of the oxidative phosphorylation (OXPHOS) were decreased in macrophages co-cultured with *P. brasiliensis* (**Table 2**).

**Table 1:** Decreased proteins in macrophages infected with *Paracoccidioides brasiliensis* yeast cells involved in glucose metabolism.

Accession number^1^	Protein description and biological process^2^	Score^3^	Fold change^4^
	Glycolysis and Gluconeogenesis		
A0A2U3TZU2	Glucose-6-phosphate isomerase	3765.44	0.394
A0A7I2V3Z0	ATP-dependent 6-phosphofructokinase	6534.87	*
P17858	ATP-dependent 6-phosphofructokinase_ liver type	670.40	*
F8VZI0	ATP-dependent 6-phosphofructokinase_ muscle type	416.68	*
Q01813	ATP-dependent 6-phosphofructokinase_ platelet type	6601.00	*
J3KPS3	Fructose-bisphosphate aldolase	31785.80	*
P05062	Fructose-bisphosphate aldolase B	1668.83	*
P09972	Fructose-bisphosphate aldolase C	12130.18	*
P60174	Triosephosphate isomerase	35540.98	0.379
P04406	Glyceraldehyde-3-phosphate dehydrogenase	21535.55	*
O14556	Glyceraldehyde-3-phosphate dehydrogenase_ testis-specific	399.52	*
P00558	Phosphoglycerate kinase 1	23595.51	*
P07205	Phosphoglycerate kinase 2	1115.35	*
P18669	Phosphoglycerate mutase 1	26342.06	*
P15259	Phosphoglycerate mutase 2	17441.32	*
Q8N0Y7	Probable phosphoglycerate mutase 4	13757.52	*
P06733	Alpha-enolase	63037.82	*
P13929	Beta-enolase	5425.63	*
P09104	Gamma-enolase	5319.81	*
E5RGZ4	2-phospho-D-glycerate hydro-lyase	5340.21	*
F5H0C8	2-phospho-D-glycerate hydro-lyase	5297.14	*
A0A2R8Y6G6	2-phospho-D-glycerate hydro-lyase	23270.41	*
P30613	Pyruvate kinase PKLR	1599.20	*
P14618	Pyruvate kinase PKM	47876.48	0.132
	Fermentation		
P11766	Alcohol dehydrogenase class-3	5335.49	*
P00338	L-lactate dehydrogenase A chain	13796.04	*
Q6ZMR3	L-lactate dehydrogenase A-like 6A	703.09	*
Q9BYZ2	L-lactate dehydrogenase A-like 6B	914.21	*
P07195	L-lactate dehydrogenase B chain	41051.41	*
P07864	L-lactate dehydrogenase C chain	748.34	*
	Glycogen metabolism		
E9PK47	Alpha-1_4 glucan phosphorylase	1890.55	*
P11216	Glycogen phosphorylase_ brain form	2313.76	0.051
P06737	Glycogen phosphorylase_ liver form	1890.55	*
P11217	Glycogen phosphorylase_ muscle form	8381.66	0.477

**^1^** Accession number of matched protein from Homo sapiens’ macrophages Uniprot database (https://www.uniprot.org/).

**^2^** Proteins annotation from Homo sapiens’ database or by homology in NCBI database (http://www.ncbi.nlm.nih.gov/) and biological process according to the classification of KEGG (https://www.genome.jp/kegg/), UniProt database (https://www.uniprot.org/), NCBI database (http://www.ncbi.nlm.nih.gov/) and CORUM database (http://mips.helmholtz-muenchen.de/corum/).

**^3^** PLGS score is the result of different mathematical models for peptide and fragment assign prediction.

**^4^** Fold-change values were obtained by dividing the values of protein abundance (in fmol) from macrophages during infection by live PB by the abundance in the uninfected macrophages. Proteins with a minimum fold-change of 50% (≤ 0.67) were considered to be dowregulated.

^*^ Proteins detected only in uninfected macrophages.

A fold equal to or less than 0.67 was considered.

**Table 2:** Decreased proteins in macrophages infected with *Paracoccidioides brasiliensis* yeast cells involved in energy production.

Accession number^1^	Protein description and biological process^2^	Score^3^	Fold change^4^
	Tricarboxylic-acid cycle		
P53396	ATP-citrate synthase	1520.56	*
O75390	Citrate synthase_ mitochondrial	12200.29	*
A2A274	Aconitate hydratase_ mitochondrial	916.17	*
P50213	Isocitrate dehydrogenase [NAD] subunit alpha_ mitochondrial	1918.82	*
O75874	Isocitrate dehydrogenase [NADP] cytoplasmic	975.87	*
P09622	Dihydrolipoyl dehydrogenase_ mitochondrial	1686.78	*
P53597	Succinate–CoA ligase [ADP/GDP-forming] subunit alpha_ mitochondrial	651.86	*
A0A5K1VW95	Malate dehydrogenase	15614.68	*
C9JRL4	Cytosolic malate dehydrogenase (Fragment)	13862.17	*
P07954	Fumarate hydratase_ mitochondrial	2669.02	*
	Oxidative phosphorylation		
P13804	Electron transfer flavoprotein subunit alpha_ mitochondrial	1834.68	*
C9J8T6	Cytochrome c oxidase copper chaperone	7210.97	*
Q5SQT6	Inorganic diphosphatase	403.07	*
H0Y9D8	Inorganic diphosphatase (Fragment)	1522.39	*
Q15181	Inorganic pyrophosphatase	721.87	*
Q9H2U2	Inorganic pyrophosphatase 2_ mitochondrial	1668.87	*
K7EJP1	ATP synthase subunit alpha_ mitochondrial (Fragment)	17166.89	*
P06576	ATP synthase subunit beta_ mitochondrial	18491.35	*
P36542	ATP synthase subunit gamma_ mitochondrial	1169.11	*
A8MUH2	ATP synthase-coupling factor 6_ mitochondrial	1566.69	*
Q93050	V-type proton ATPase 116 kDa subunit a1	2050.49	*
O75323	Protein NipSnap homolog 2	2351.76	*

**^1^** Accession number of matched protein from Homo sapiens’ macrophages Uniprot database (https://www.uniprot.org/).

**^2^** Proteins annotation from Homo sapiens’ database or by homology in NCBI database (http://www.ncbi.nlm.nih.gov/) and biological process according to the classification of KEGG (https://www.genome.jp/kegg/), UniProt database (https://www.uniprot.org/), NCBI database (http://www.ncbi.nlm.nih.gov/) and CORUM database (http://mips.helmholtz-muenchen.de/corum/).

**^3^** PLGS score is the result of different mathematical models for peptide and fragment assign prediction.

**^4^** Fold-change values were obtained by dividing the values of protein abundance (in fmol) from macrophages during infection by live PB by the abundance in the uninfected macrophages. Proteins with a minimum fold-change of 50% (≤ 0.67) were considered to be dowregulated.

^*^ Proteins detected only in uninfected macrophages.

A fold equal to or less than 0.67 was considered.

**Figure 3:**
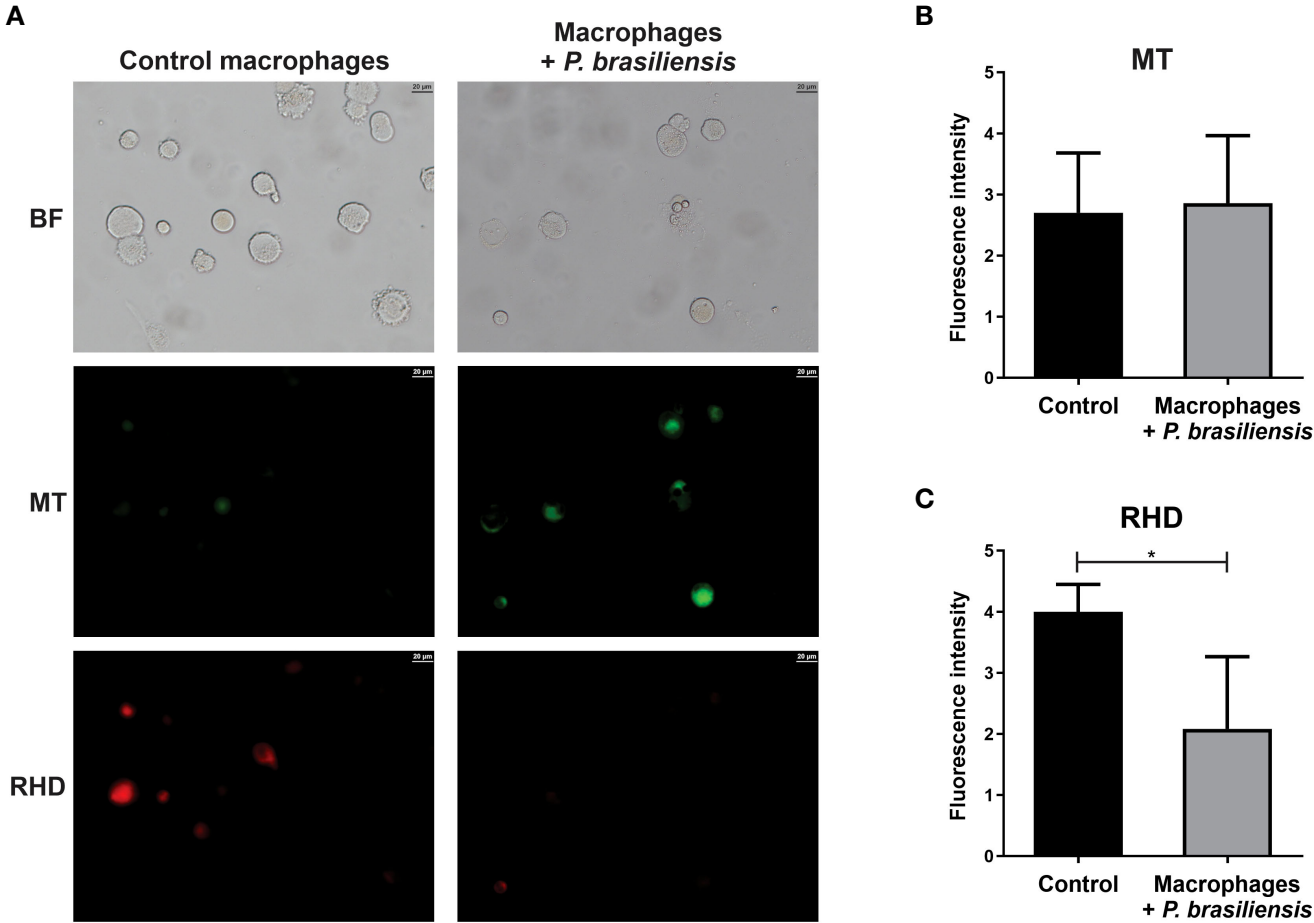
Mitochondrial membrane activity in THP-1 macrophages co-cultured with *Paracoccidioides brasiliensis* yeast cells (for 24 h). Mitochondria integrity and activity was analyzed by fluorescence microscopy using MitoTracker Green FM and Rhodamin 123 dyes, respectively. **(A)** Macrophages in absence or presence of *P. brasiliensis* were photographed using an Axio-Scope A1 microscope at 400x magnification. **(B)** Fluorescence intensity (in pixels) of cells stained with MT **(C)** Fluorescence intensity (in pixels) of cells stained with RDH. Data represent the mean and standard deviation of three biological replicates. Brightfield (BF), Rhodamine 123 (RDH) and MitoTracker Green (MT). *p ≤ 0.05 by using Student’s t-test.

As enzymes related to the electron transport chain were decreased in *P. brasiliensis*-exposed macrophages, we assessed whether the integrity and activity of mitochondria in macrophages were impaired. There was no difference in mitochondrial membrane integrity in macrophages co-cultured or not with *P. brasiliensis* yeasts (**Figure 3**). Photomicrographs presented in **Figure 3A** show macrophages with fluorescence of MitoTracker Green FM whereas in **Figure 3B**, the results of pixels of fluorescence quantification.We observed a significant decrease in mitochondrial activity in macrophages co-cultured with *P. brasiliensis* compared to the controls (**Figure 3**, p < 0.05). In **Figure 3A** the photomicrographs of cells and in 3C the quantification of pixels of fluorescence of Rhodamine 123 in macrophages exposed to the fungi was lower than in control macrophages. Therefore, the data indicate that *P. brasiliensis* infection affects mitochondrial activity what is probably due to the repression of enzymes in energy production pathways.

### 3.4 *P. brasiliensis* regulated detoxification proteins and increased ROS production in THP-1 macrophages

*P. brasiliensis* induced changes in the proteins that play a role in the stress response of THP-1 macrophages. Among the repressed proteins related to the stress response, three proteins of the heat shock family were identified as well as catalase, glutathione reductase_mitochondrial, glutathione S-transferase omega-1, peroxiredoxin-1, 4, 5 and 6, superoxide dismutase [Cu-Zn] (SOD1), and five proteins of the thioredoxin system (**Table 3**). On the other hand, among the *P. brasiliensis*-induced proteins, glutathione peroxidase 1(GPX1), glutathione S-transferase kappa (GSTK1), glutathione S-transferase P (GSTP1), and peroxiredoxin-2 (PRDX2) (**Table 4**), all very important in the response to oxidative stress, were identified.

**Table 3:** Decreased proteins in macrophages infected with live *Paracoccidioides brasiliensis* yeast cells involved in stress response.

Accession number^1^	Protein description and biological process^2^	Score^3^	Fold change^4^
	Stress response		
A0A7I2V2S7	75 kDa glucose-regulated protein	7343.21	0.677
P04040	Catalase	6229.48	*
P00390	Glutathione reductase_ mitochondrial	3504.57	*
P78417	Glutathione S-transferase omega-1	1469.15	*
Q5TA02	Glutathione-dependent dehydroascorbate reductase (Fragment)	1469.15	*
A8MX94	GST class-pi	21612.53	0.386
A0A6Q8PGK1	Heat shock 27 kDa protein	30162.08	*
H0Y8K0	Heat shock 70kDa protein 9B (Mortalin-2)_ isoform CRA_a	5920.49	*
P04792	Heat shock protein beta-1	30168.58	*
H3BSU0	Liver carboxylesterase 1 (Fragment)	407.80	*
P55145	Mesencephalic astrocyte-derived neurotrophic factor	12025.95	*
G5E977	Nicotinate phosphoribosyltransferase	2537.61	*
A1KZ92	Peroxidasin-like protein	344.36	*
A0A0A0MRQ5	Peroxiredoxin-1	6482.96	*
Q13162	Peroxiredoxin-4	8387.69	*
P30044	Peroxiredoxin-5_ mitochondrial	1369.47	*
P30041	Peroxiredoxin-6	13986.24	*
P10768	S-formylglutathione hydrolase	1667.33	*
P38646	Stress-70 protein_ mitochondrial	7419.77	0.670
P31948	Stress-induced-phosphoprotein 1	10056.83	*
P00441	Superoxide dismutase [Cu-Zn]	8491.53	*
P10599	Thioredoxin	7245.89	*
O95881	Thioredoxin domain-containing protein 12	1161.27	*
Q16881	Thioredoxin reductase 1_ cytoplasmic	4239.45	*
P30048	Thioredoxin-dependent peroxide reductase_ mitochondrial	5641.69	*
A0A182DWI3	Thioredoxin-disulfide reductase	4235.96	*

**^1^** Accession number of matched protein from Homo sapiens’ macrophages Uniprot database (https://www.uniprot.org/).

**^2^** Proteins annotation from Homo sapiens’ database or by homology in NCBI database (http://www.ncbi.nlm.nih.gov/) and biological process according to the classification of KEGG (https://www.genome.jp/kegg/), UniProt database (https://www.uniprot.org/), NCBI database (http://www.ncbi.nlm.nih.gov/) and CORUM database (http://mips.helmholtz-muenchen.de/corum/).

**^3^** PLGS score is the result of different mathematical models for peptide and fragment assign prediction.

**^4^** Fold-change values were obtained by dividing the values of protein abundance (in fmol) from macrophages during infection by live PB by the abundance in the uninfected macrophages. Proteins with a minimum fold-change of 50% (≤ 0.67) were considered to be dowregulated.

^*^ Proteins detected only in uninfected macrophages.

A fold equal to or less than 0.67 was considered.

**Table 4:** Increased proteins in macrophages infected with *Paracoccidioides brasiliensis* yeast cells involved in stress response.

Accession number^1^	Protein description and biological process^2^	Score^3^	Fold change^4^
	Stress response		
P07203	Glutathione peroxidase 1	4743.71	2.225
Q9Y2Q3	Glutathione S-transferase kappa 1	843.45	*
P09211	Glutathione S-transferase P	22608.57	1.682
K7EN27	Maillard deglycase (Fragment)	13395.05	2.117
K7ELW0	Parkinson disease protein 7	17649.65	3.706
P32119	Peroxiredoxin-2	1915.38	1.896

**^1^** Accession number of matched protein from Homo sapiens’ macrophages Uniprot database (https://www.uniprot.org/).

**^2^** Proteins annotation from Homo sapiens’ database or by homology in NCBI database (http://www.ncbi.nlm.nih.gov/) and biological process according to the classification of KEGG (https://www.genome.jp/kegg/), UniProt database (https://www.uniprot.org/), NCBI database (http://www.ncbi.nlm.nih.gov/) and CORUM database (http://mips.helmholtz-muenchen.de/corum/).

**^3^** PLGS score is the result of different mathematical models for peptide and fragment assign prediction.

**^4^** Fold-change values were obtained by dividing the values of protein abundance (in fmol) from macrophages during infection by live PB by the abundance in the uninfected macrophages. Proteins with a minimum fold-change of 50% (≥ 1.5) were considered to be upregulated.

^*^ Proteins detected only in infected macrophages.

A fold equal to or higher than 1.5 was considered.

**Figure 4:**
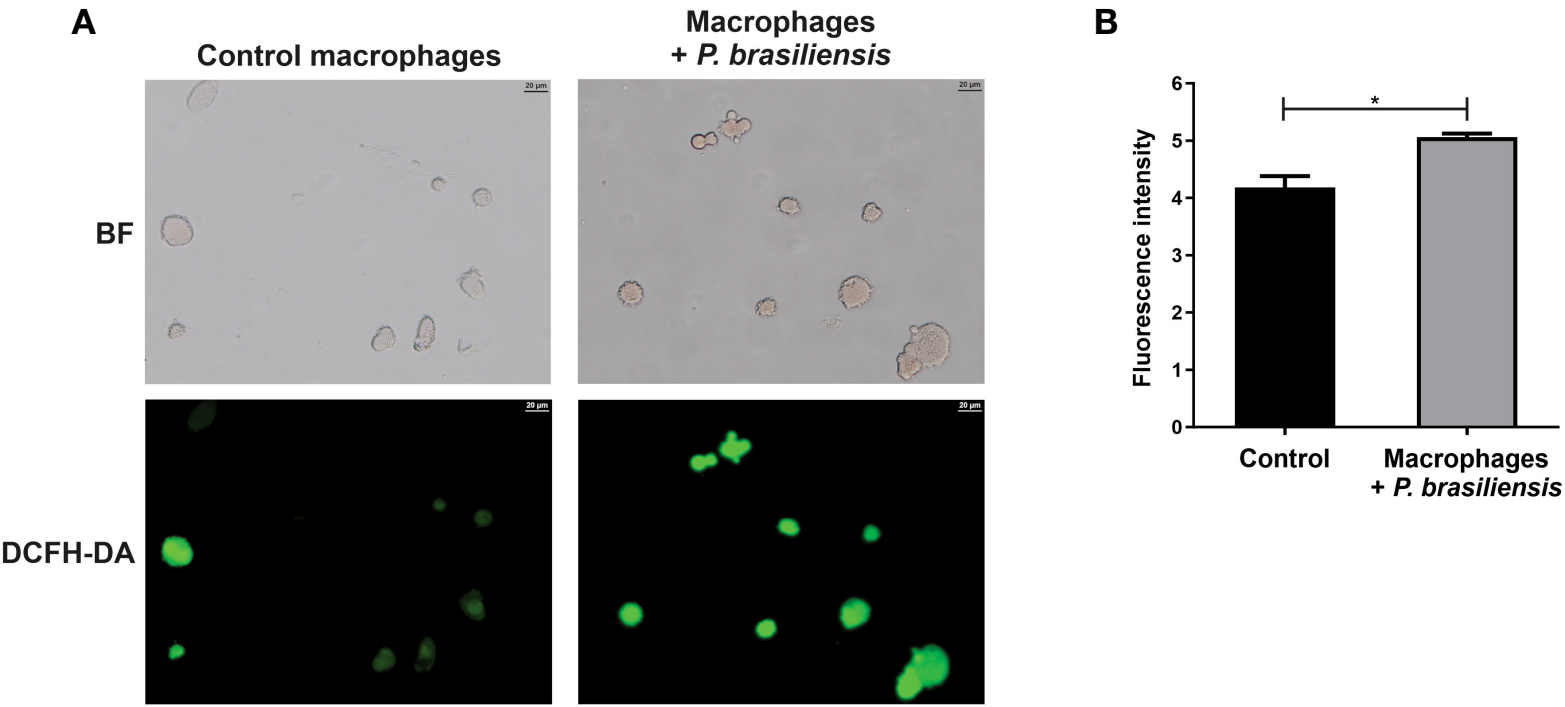
Production of reactive oxygen species in THP-1 macrophages co-cultured with *P. brasiliensis* yeast cells (for 24 h). Macrophages were stained with 2’,7’-dichlorodihydrofluorescein diacetate (DCFH-DA). **(A)** Macrophages were photographed using an Axio-Scope A1 microscope at 400× magnification. **(B)** Fluorescence intensities (in pixels) of cells stained with DCFH. Brightfield (BF) and 2’,7’-dichlorodihydrofluorescein diacetate (DCFH-DA). Data represent the mean and standard deviation of three biological replicates. *p ≤ 0.05 by using Student’s t-test.

The ROS are generated during oxidative phosphorylation, but the mitochondrial dysfunction can lead to an exacerbated ROS production, which can cause tissue damage (Peoples et al., 2019; Rizwan et al., 2020). Since we have observed a decrease in mitochondrial activity in macrophages exposed to *P. brasiliensis* as well as a regulation of proteins relative to oxidative stress response, we investigated whether those macrophages could produce ROS. In fact, we observed that macrophages co-cultured with yeasts showed higher ROS production than control cells (an increase of 18%), as shown in **Figure 4**.

### 3.5 Proteins of DNA processing, cell cycle, and transcription are regulated in THP-1 macrophages co-cultured with *P. brasiliensis*

Co-culture with *P. brasiliensis* and macrophages caused a repression of 21 and 17 proteins that play a role in DNA processing and cell cycle in macrophages, respectively. Some of these proteins are important in the process of DNA replication and repair such as ATP-dependent DNA helicase 2 subunit 1, DNA damage-binding protein 1 and proliferating cell nuclear antigen. Other proteins, which play a role in chromatin remodeling such as histones H1.2, H1.3, H1.4, H2A type 3, and HIST2H3PS2 were reduced (**Supplementary Table 1**). Among the proteins increased in macrophages co-cultured with *P. brasiliensis* involved in DNA processing (**Supplementary Table 2**) there are two subunits of the DNA polymerase enzyme, which plays a key role in DNA replication, and three other proteins involved in chromatin remodeling [Lethal(3)malignant brain tumor-like protein 3 (L3MBTL3), Lysine-specific demethylase 5A (KDM5A) and SWI/SNF-related matrix-associated actin-dependent regulator of chromatin subfamily A containing DEAD/H box 1 (SMARCAD1)], which means that access to chromatin is altered and, consequently, there are changes in gene expression. Regarding the cell cycle, the decreased proteins are those ones involved with the organization and maintenance of microtubules, formation of the mitotic spindle, progression and regulation of the cell cycle, and histone transport (**Supplementary Table 1**). Therefore, the repression of these proteins can deregulate the cell cycle, causing damage to the cell or decreasing its ability to proliferate.

It is also relevant to highlight that 61 proteins involved in gene transcription control were negatively (**Supplementary Table 1**) whereas 22 proteins were positively regulated (**Supplementary Table 2**). Among the repressed proteins, the Forkhead box protein M1 transcription factor (FOXM1), Hox family proteins, and several zinc finger proteins were identified (**Supplementary Table 1**). The presence of the fungus also induced transcription elongation factor SPT6 (SPT6), some zinc finger proteins and the FOX transcription factor family proteins (FOX) C1, C2, G1, and L1 (**Supplementary Table 2**).

### 3.6 *P. brasiliensis* regulated many proteins of the immune responses in host macrophages

Regarding the immune response, 88 proteins were negatively regulated in macrophages exposed to *P. brasiliensis* (**Table 5**), among them: protein subunits of the Arp2/3 complex, Actin-like protein 3, Arf-GAP with SH3 domain _ ANK repeat and PH domain-containing protein 2 and Cofilin-2, which are relevant for cell adhesion and phagocytosis. Protein as Toll-like receptor (TLR)-9 that recognizes pathogen DNA was also decreased in the proteoma. Proteins of antigen processing were also identified such as Cathepsin B and S, Endoplasmic reticulum chaperone BiP, Heat shock-related 70 kDa protein 2, and Ras-related protein Rab-3C. Thirty-five histones participating in the formation of extracellular traps (ETs) were identified. Other proteins are related to the inflammatory process (Annexin, Macrophage migration inhibitory factor), the complement system (Complement component 1 Q subcomponent-binding protein_mitochondrial), leukocyte adhesion, and transmigration (Integrin beta-2, Lymphocyte-specific protein 1), differentiation of lymphocytes [Chitinase-3-like protein 1, Transforming growth factor beta (TGFβ-1), Retinoic acid receptor alpha, beta, and gamma], among other processes (**Table 5**).

**Table 5:** Some proteins decreased in macrophages infected vwith *Paracoccidioides brasiliensis* yeast cells involved in immune response.

Accession number^1^	Protein description and biological process^2^	Score^3^	Fold change^4^
	Immune response		
O15143	Actin-related protein 2/3 complex subunit 1B	8740.81	0.631
O15144	Actin-related protein 2/3 complex subunit 2	3617.00	*
O15145	Actin-related protein 2/3 complex subunit 3	17431.37	*
R4GN08	Actin-related protein 2/3 complex subunit 4 (Fragment)	1106.88	*
O15511	Actin-related protein 2/3 complex subunit 5	3627.45	*
F8WE84	Actin-related protein 3	9712.19	*
D6RBL5	Annexin	13850.32	0.650
P04083	Annexin A1	18072.62	*
P08758	Annexin A5	30602.12	0.644
O43150	Arf-GAP with SH3 domain_ ANK repeat and PH domain-containing protein 2	594.14	*
G5E9J0	Arp2/3 complex 34 kDa subunit	475.38	*
P61769	Beta-2-microglobulin	3926.66	*
A0A7I2V440	Cathepsin B	6042.11	*
P25774	Cathepsin S	1155.57	*
P36222	Chitinase-3-like protein 1	1597.86	*
Q07021	Complement component 1 Q subcomponent-binding protein_ mitochondrial	7230.48	*
A0A7P0TB36	Endoplasmic reticulum chaperone BiP	21012.03	0.582
P54652	Heat shock-related 70 kDa protein 2	11903.81	0.001
P09429	High mobility group protein B1	1668.83	*
C9J0D1	Histone H2A	7828.83	*
P0C0S8	Histone H2A type 1	8902.71	*
Q96QV6	Histone H2A type 1-A	3271.32	*
P04908	Histone H2A type 1-B/E	8902.71	*
Q93077	Histone H2A type 1-C	8902.71	*
P20671	Histone H2A type 1-D	8902.71	*
Q96KK5	Histone H2A type 1-H	8902.71	*
Q99878	Histone H2A type 1-J	8902.71	*
Q6FI13	Histone H2A type 2-A	8902.71	*
Q8IUE6	Histone H2A type 2-B	2747.75	*
Q16777	Histone H2A type 2-C	8902.71	*
Q9BTM1	Histone H2A.J	8902.71	*
Q71UI9	Histone H2A.V	8676.91	*
P0C0S5	Histone H2A.Z	8676.91	*
P16104	Histone H2AX	3273.08	*
U3KQK0	Histone H2B	16807.41	*
Q96A08	Histone H2B type 1-A	7130.46	*
P33778	Histone H2B type 1-B	17593.68	*
P62807	Histone H2B type 1-C/E/F/G/I	16807.41	*
P58876	Histone H2B type 1-D	16807.41	*
Q93079	Histone H2B type 1-H	16807.41	*
P06899	Histone H2B type 1-J	17593.68	*
O60814	Histone H2B type 1-K	16807.41	*
Q99880	Histone H2B type 1-L	16807.41	*
Q99879	Histone H2B type 1-M	16807.41	*
Q99877	Histone H2B type 1-N	16807.41	*
P23527	Histone H2B type 1-O	17593.68	*
Q16778	Histone H2B type 2-E	17593.68	*
A0A2R8Y619	Histone H2B type 2-E1	311.58	*
Q5QNW6	Histone H2B type 2-F	16807.41	*
Q8N257	Histone H2B type 3-B	17593.68	*
P57053	Histone H2B type F-S	16800.03	*
Q6NXT2	Histone H3.3C	3853.37	*
P62805	Histone H4	7844.59	*
P05107	Integrin beta-2	2518.87	*
P33241	Lymphocyte-specific protein 1	820.58	*
P14174	Macrophage migration inhibitory factor	4042.46	*
Q96E17	Ras-related protein Rab-3C	1115.57	*
P19793	Retinoic acid receptor RXR-alpha	654.98	*
P28702	Retinoic acid receptor RXR-beta	1177.33	*
P48443	Retinoic acid receptor RXR-gamma	630.23	*
H0Y858	Toll-like receptor 9 (Fragment)	624.89	*
A0A499FJK2	Transforming growth factor beta	618.77	*

**^1^** Accession number of matched protein from Homo sapiens’ macrophages Uniprot database (https://www.uniprot.org/).

**^2^** Proteins annotation from Homo sapiens’ database or by homology in NCBI database (http://www.ncbi.nlm.nih.gov/) and biological process according to the classification of KEGG (https://www.genome.jp/kegg/), UniProt database (https://www.uniprot.org/), NCBI database (http://www.ncbi.nlm.nih.gov/) and CORUM database (http://mips.helmholtz-muenchen.de/corum/).

**^3^** PLGS score is the result of different mathematical models for peptide and fragment assign prediction.

**^4^** Fold-change values were obtained by dividing the values of protein abundance (in fmol) from macrophages during infection by live PB by the abundance in the uninfected macrophages. Proteins with a minimum fold-change of 50% (≤ 0.67) were considered to be dowregulated.

^*^ Proteins detected only in uninfected macrophages.

A fold equal to or less than 0.67 was considered.

**Figure 5:**
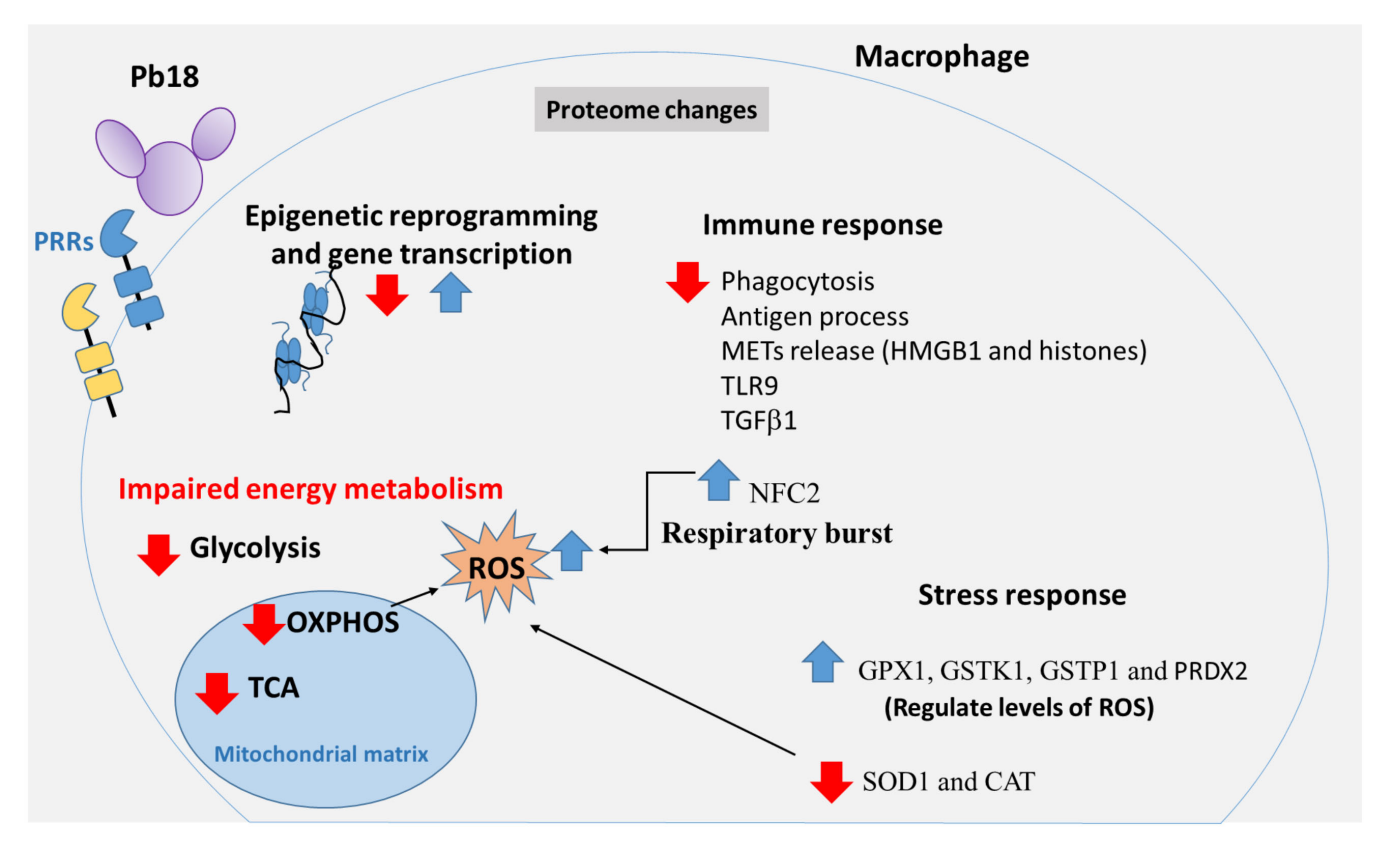
Graphical summary of proteomic changes in human THP-1 macrophages co-cultured with *Paracoccidioides brasiliensis*. *Pb*18 yeast cells induce changes in the macrophage proteome. The interactions between Pb18 yeast cells and macrophages include recognition of the fungal PAMPs by macrophage PRRs as well as other fungal and host factors which induce changes in the proteome of macrophages. Co-cultures of THP-1 macrophages and *P. brasiliensis* yeasts caused increase and decrease of macrophage proteins participating in epigenetic reprogramming, and gene transcription processes such as several histones, L3MBTL3, KDM5A, SMARCAD1, FOXM1, SPT6, FOXC1 and ZN140. *P. brasiliensis*-exposed macrophages showed impaired glycolysis, TCA and OXPHOS pathways, and increased ROS production. In addition, *P. brasiliensis* repressed several proteins of the immune system, relevant for phagocytosis, antigen processing and presentation, besides components of METs, innate immune receptors, cytokines, among others. Macrophage interaction with *P. brasiliensis* also increases NCF2, a functional subunit of NADPH oxidase complex, which may contribute to the ROS production. Some enzymes involved with oxidative stress were also increased (GPX1, GSTK1, GSTP1, and PRDX2). Other proteins related to the reaction to oxidative stress were repressed: SOD1 and catalase CAT. Pattern-recognizing receptors (PRRs); Tricarboxylic acid cycle (TCA); Phosphorylative oxidation (OXPHOS); Reactive oxygen species (ROS); Extracellular traps of macrophages (METs); Neutrophil cytosol factor 2 (NCF2); Glutathione peroxidase 1 (GPX1); Glutathione S-transferase kappa (GSTK1); Glutathione S-transferase P (GSTP1); Peroxiredoxin-2 (PRDX2); Superoxide dismutase (SOD1); Catalase (CAT).

Our analysis identified 14 proteins related to immune response that were increased in macrophages exposed to *P. brasiliensis* (**Table 6**). Among them we highlight proteins associated to inflammation (Caspase recruitment domain family_ member 14_ isoform CRA_d), proteins involved in the adhesion process, related to leukocyte transmigration and phagocytosis (Actin_cytoplasmic 2, Ezrin, Moesin, Stabilin-2). Protein involved in the antigen processing (Heat shock cognate 71 kDa protein, Heat shock 70 kDa protein 1A, Heat shock 70 kDa protein 1B, Heat shock 70 kDa protein 6) and respiratory burst (Neutrophil cytosol factor 2 - NCF2), were also increased in the macrophage during infection with *P. brasiliensis.* **Figure 5** shows an overview of the proteome changes observed in THP-1 macrophages exposed to *P. brasiliensis*.

**Table 6:** Increased proteins in macrophages infected with *Paracoccidioides brasiliensis* yeast cells involved in immune response.

Accession number^1^	Protein description and biological process^2^	Score^3^	Fold change^4^
	Immune response		
A0A7P0TBL1	Actin_ cytoplasmic 2	1198.38	314.190
P61160	Actin-related protein 2	5478.23	2.013
Q7Z6M3	Allergin-1	326.88	*
I3L414	Caspase recruitment domain family_ member 14_ isoform CRA_d	406.41	37.712
P15311	Ezrin	1969.29	2.293
V9GZ37	Heat shock 70 kDa protein 1A	129.76	*
A0A0G2JIW1	Heat shock 70 kDa protein 1B	2588.57	5.365
P17066	Heat shock 70 kDa protein 6	2359.95	196.369
E9PQQ4	Heat shock cognate 71 kDa protein (Fragment)	21826.13	5.312
A0A1B0GU45	Inositol 1_4_5-triphosphate receptor-associated 2	514.40	*
P26038	Moesin	11172.13	2.534
P19878	Neutrophil cytosol factor 2	334.05	*
Q06330	Recombining binding protein suppressor of hairless	338.53	*
Q8WWQ8	Stabilin-2	238.15	*

^1^ Accession number of matched protein from Homo sapiens’ macrophages Uniprot database (https://www.uniprot.org/).

^2^ Proteins annotation from Homo sapiens’ database or by homology in NCBI database (http://www.ncbi.nlm.nih.gov/) and biological process according to the classification of KEGG (https://www.genome.jp/kegg/), UniProt database (https://www.uniprot.org/), NCBI database (http://www.ncbi.nlm.nih.gov/) and CORUM database (http://mips.helmholtz-muenchen.de/corum/).

^3^ PLGS score is the result of different mathematical models for peptide and fragment assign prediction.

^4^ Fold-change values were obtained by dividing the values of protein abundance (in fmol) from macrophages during infection by live PB by the abundance in the uninfected macrophages. Proteins with a minimum fold-change of 50% (≥ 1.5) were considered to be upregulated.

^*^ Proteins detected only in infected macrophages.

A fold equal to or higher than 1.5 was considered.

## 4 Discussion

In the present study, we have performed a proteomic analysis of the THP-1 macrophage in response to interaction with *P. brasiliensis* yeast cells. Since cells and fungi were co-cultured, the macrophage proteome was a result of the fungus effects on infected and bystander non-infected macrophages for 24 h of incubation. Although a survival assay was not performed to evaluate fungal replication within 24 h, we have shown that macrophages were efficiently infected by yeast cells. There is no information in the literature about the growth of *P. brasiliensis* in THP-1 macrophages; however, we have demonstrated that the number of viable fungal cells (assessed by the number of colony-forming units) increases during murine alveolar macrophages infection (AMJ2-C11) from 6 hours and continues to increase up to 12 hours after interaction (Chaves et al., 2019). Thus, probably the fungi proliferated during the incubation time (24 h) in THP-1 macrophages.

We showed that *P. brasiliensis* infection regulates proteins of a variety of biological processes. To respond to an infection, immune cells undergo a metabolic adaptation, which is coupled to the effector mechanisms of the immune response, which require energy and/or specific metabolites (Domínguez-Andrés et al., 2017; Tucey et al., 2018). On the other hand, the pathogen also undergoes changes and tries to escape the immune system (De Arruda Grossklaus et al., 2013; Parente-Rocha et al., 2015; Lacerda Pigosso et al., 2017; Weerasinghe and Traven, 2020). In recent years, some studies in the field of immunometabolism have been carried out, with the aim of understanding the role of metabolic events in regulating the response of immune cells to pathogens (O’Neill et al., 2016; Domínguez-Andrés et al., 2017; Tucey et al., 2018; Weerasinghe and Traven, 2020).

Of relevance, in this study, proteins of the cellular processes for energy production decreased in macrophages interacting with *P. brasiliensis*. In these cells, it was detected a decreased glycolysis, TCA, and oxidative phosphorylation. Proteomic data were confirmed by assessing the mitochondrial activity, which was decreased as tested by staining with Rhodamine 123. Studies have shown that recognition of some PAMPs by monocytes and macrophages induces aerobic glycolysis, also called as Warburg effect. This effect consists in increased glucose consumption, repression of the TCA and OXPHOS as well as increased lactate production. These metabolic effect are triggered, for example, in monocytes and macrophages after recognition of β-glucans from *Candida albicans* (Cheng et al., 2014; Domínguez-Andrés et al., 2017) or melanin in *Aspergillus fumigatus* (Gonçalves et al., 2020). However, this change in metabolism is not the rule for all pathogens and is probably due to differences in PRRs triggered by PAMPs and other intrinsic factors produced by each microorganism. Similar to our result, Shin et al. (2005) observed in a proteomic analysis a decrease in energy metabolism enzymes in murine macrophages infected with *C. albicans* hyphae and this contributes along with other factors to host cell death. However, in murine alveolar epithelial cells infected with *A. fumigatus*, enrichment of phosphorylative oxidation proteins was observed, indicating increased energy production (Seddigh et al., 2017). Also according to our findings, it has already been demonstrated that *Salmonella typhimurium* compromises glycolysis, impairing phagosome acidification and, consequently, decreasing bacterial clearance in murine macrophages (Gutierrez et al., 2020). Additionally, McGettrick et al. (2016) showed that the metabolite indolepyruvate from *Trypanosoma brucei* decreases glycolysis and hypoxia-inducible transcription factor-1α (HIF-1α) in murine macrophages as an evasion mechanism. Therefore, we can suggest that *P. brasiliensis* somehow affects energy production pathways in the host cells in an attempt to benefit itself.

In the present study, proteins of cellular stress response have been identified. Among the increased proteins are three enzymes of glutathione metabolism (GPX1, GSTK1 and GSTP1) and PRDX2, important cellular antioxidants, which acting to regulate necessary levels of ROS (Hayes and McLellan, 1999; Rhee, 2016). On the contrary, the enzymes SOD1, and catalase were decreased. SOD1 catalyzes the dismutation of superoxide anion (O2^·-^) into oxygen and hydrogen peroxide (H_2_O_2_) whereas catalase does the decomposition of H_2_O_2_ into water and oxygen. It has been demonstrated that H_2_O_2_ and O_2_^·-^ are important effector molecules to kill *P. brasiliensis* in human macrophages and neutrophils activated by cytokines (Carmo et al., 2006; Rodrigues et al., 2007). The lack of these enzymes can lead to a significant increase in ROS and, consequently, cause exacerbated tissue damage. Nevertheless, in some cases, it can be important to increase ROS at toxic levels to eliminate pathogens (Gebicka and Krych-Madej, 2019). In our study, we demonstrated that there is an increase in ROS production by macrophages interacting with *P. brasiliensis*, thus suggesting that *P. brasiliensis*-infected macrophages can be regulated by ROS. We need to check whether the levels of ROS produced during the first 24 h of macrophage and fungus interaction are able to directly kill the fungus or are used to regulate immune responses, for example by activating inflammasome (Tavares et al., 2013).

Proteomic analysis identified regulation of several proteins that play important roles in DNA replication and transcription processes. Some histones, enzymes and transcription factors that contribute to epigenetic modifications have changed in macrophages after interaction with *P. brasiliensis*. This has implications for chromatin accessibility by the transcription machinery. Epigenetic remodeling is related to metabolic changes, since some metabolites act as cofactors for enzymes that regulate chromatin modifications and, consequently, alter gene transcription and cell proteome (Netea et al., 2016; Sun et al., 2022). The increased proteins L3MBTL3 and KDM5A, participate in demethylation of lysine 4 of histone 3 (H3K4) (Christensen et al., 2007; Xu et al., 2017), thus causing gene repression, since H3K4 methylation is associated with decondensed chromatin (Ciccone and Chen, 2009). Another increased protein was SMARCAD1, which mediates the deacetylation of histones 3 and 4, which can be related to gene repression. Deletion of SMARCAD1 in mice with colitis leads to increased expression of several genes, including those related to innate immune response (Kazakevych et al., 2020). Nevertheless, the SPT6 also increased here and participates in the demethylation of lysine 27 of histone 3 (H3K27) (Wang et al., 2013) and the methylation of lysine 36 of the same histone (Gopalakrishnan et al., 2019). Thus, SPT6 helps the opening of chromatin and activation of transcription. Therefore, the increase of the SMARCAD1 protein as well as L3MBTL3 and KDM5A in the present study, probably contributed to repress several genes. The induction of SPT6 could have induced the expression of other genes, which is consistent with changes found in the proteome of macrophages interacting with *P. brasiliensis*.

Among the increased proteins that have functions in transcription process, Forkhead box protein C1 (FOXC1), and Zinc finger protein 140 (ZN140) also called our attention. Zhang et al. (2019) have demonstrated that FOXC1 increases the expression of TLR3, and TLR4, and is related to pro-inflammatory effects in a murine model of cardiac ischemia. One study suggested that TLR3 is a negative regulator of host response against *P. brasiliensis*, what was associated with a decrease of the proinflammatory response, NO production, and activation of CD8^+^ T lymphocytes (Jannuzzi et al., 2019). It has also been demonstrated that TLR4 is relevant to the production of IFNγ in human neutrophils infected with *P. brasiliensis* (Rodrigues et al., 2014). Besides, TLR4 cooperates with dectin-1 (for β-glucan) and mannose receptor in the differentiation of Th17 cells stimulated by murine dendritic cells (Loures et al., 2015). Although the cytokine IFNγ and Th17 cells are important for fungal control, studies have associated TLR4 with the pathogenesis of PCM (Acorci-Valério et al., 2010; Loures et al., 2010). We cannot identify TLR4 in this study, but we can speculate that by contributing to the production of IFNγ and the differentiation of Th17 cells, TLR4 may lead to a mix of Th1/Th17 response that is associated with the chronic form of PCM, and crucial for the control of the infection (De Castro et al., 2013). The ZN140 protein was associated with gene repression of the Fc gamma (γ) RIIB receptor (Nishimura et al., 2001). This receptor inhibits phagocytosis and release of some pro-inflammatory cytokines in macrophages, and is important for a balance between mechanisms of pathogen elimination and exacerbated inflammation (Clynes et al., 1999; Clatworthy and Smith, 2004). There are no studies with this receptor in PCM, however, in a murine model, FcγRIIB deficiency was associated with severe cryptococcosis, increased phagocytosis, and cytokine production (Surawut et al., 2017). Although FcγRIIB, TLR3 and TLR4 were not identified in our proteomic analysis, it can be suggested that ZN140 increase leads to inhibition of FcγRIIB expression, and that FOXC1 increases the expression of these TLRs in the context of PCM and that this has some relevance for the control of *P. brasiliensis.*

Among the repressed proteins, it is relevant to mention the nuclear phosphoprotein nucleolin, which is important to the processes of synthesis and maturation of ribosomes (Ginisty et al., 1999). Therefore, repression of this protein must have impaired ribosome formation and protein synthesis in macrophages co-cultured with *P. brasiliensis*. In line with this, our analysis of the proteome from those macrophages identified 83 ribosomal proteins that were decreased (**Supplementary Table 1**). Another repressed protein was Forkhead box protein M1 (FOXM1). Inhibition of this transcription factor has already been shown to increase production of ROS in diabetic wounds (Sawaya et al., 2022). Similarly, in our study we have also observed an increase of ROS in macrophages exposed to *P. brasiliensis.*

Recognition of the pathogen initiates changes in defense cells that work for controlling the infection; however, these changes may also provide opportunities for the fungus to evade the immune system (Weerasinghe and Traven, 2020). Here, proteomic analysis of host defense cells co-cultured with *P. brasiliensis* showed that the fungus regulates the expression of different proteins of the immune system. Previously, Silva et al. (2008) evaluated a group of genes in murine macrophages infected with opsonized yeasts of *P. brasiliensis.* They described alterations in the transcription of several genes of the immune response. After 24 h of infection, 59 genes were differentially expressed, 54 of which were induced and five were repressed. Genes related to inflammation, regulation of transcription, and signal transduction pathways, phagocytosis and the process of apoptosis were increased (Silva et al., 2008). Unlike the results by Silva et al. (2008), our proteomic analysis showed that human THP-1 macrophages co-cultured with *P. brasiliensis* showed predominantly the repression of several proteins for PAMP recognition in endosome (TLR9), adhesion, and phagocytosis as well as a decrease of some proteins that participate in the inflammatory process. The differences are probably due to the models and experimental procedures used in each study as well as to different levels of gene expression and protein production. It is important to consider that not everything that is transcribed is translated into protein.

We also observed a decrease of protein processing and antigen presentation pathways. A result consistent with this is the positive regulation of the KDM5A enzyme mentioned above. A study has shown that this demethylase silences genes in the antigen processing pathway during antitumor immune responses (Liu et al., 2022). In the current study, the analysis also identified repression of 34 histones related to the formation of ETs. It has already been demonstrated that *P. brasiliensis* induces the release of neutrophil ETs (NETs) that contribute to the fungus control in human neutrophils (Bachiega et al., 2016). To our knowledge up to now, there are no studies showing the release of ETs by macrophages (METs) infected with *P. brasiliensis*; however the release of METs by THP-1 macrophages infected with *Trichomonas vaginalis* has already been demonstrated (Fei et al., 2019). Another repressed protein, high mobility group protein B1 (HMGB1) is also associated with the NET formation. HMGB1 is a nuclear protein that acts as a molecular pattern associated with damage (DAMP) and its interaction with the TLR4 triggers increased NET release by murine neutrophils (Tadie et al., 2013). Therefore, our data suggest that *P. brasiliensis* may impair the release of ETs by THP-1 macrophages in a HMGB1-dependent manner.

Among the decreased proteins in macrophages co-cultured with *P. brasiliensis*, it is also relevant to discuss TLR9 in detail. The TLR9 is an endosome receptor mainly present in innate immunity cells that recognizes unmethylated CpG dinucleotides. Studies demonstrated that TLR9 is important for the survival of mice during the first 48 hours of infection by *P. brasiliensis* (Menino et al., 2013). Additionally, Morais et al. (2016) confirmed the role of TLR9 in the initial protection against *P. brasiliensis*, showing that immunization of mice with the recombinant protein rPb27 associated with CPG oligodeoxynucleotide motifs provides high protection against the fungus, 30 days after infection. Another important protein, which was decreased in the macrophage proteome of the present study is TGFβ-1, a multifunctional cytokine that regulates several processes of the immune responses including the differentiation of Th17 cells (Mangan et al., 2006), and the regulation of the healing process (Lodyga and Hinz, 2020). Restrepo et al. (1998) showed, in a murine model, that pulmonary fibrosis induced by *P. brasiliensis* conidia was associated with an increased TGFβ-1 production. Another study showed that peripheral blood monocytes from patients with PCM, evaluated before and 20 days after treatment, produced elevated endogenous levels of TGFβ-1 compared to healthy controls, and this cytokine was present in fibrotic areas of the patients’ lesions (Parise-Fortes et al., 2006). Therefore, it appears that this cytokine plays an important role in tissue repair as well as in fibrosis during PCM. In the current study, TGFβ-1 was repressed during the first 24 h of macrophage-*P. brasiliensis* interaction probably to avoid early suppression of inflammatory process or innate immunity.

Among the increased proteins, neutrophil cytosol factor 2 (NFC2) was identified; this protein is a functional subunit of NADPH oxidase complex, which is very important for the production of ROS during the process of respiratory burst in phagocytes (Nauseef, 2004). Besides the role of NADPH oxidase complex to ROS production, which are important microbicidal molecules, this enzyme complex may play other roles in the immune response (Hogan and Wheeler, 2014) such as induction of NETs, and inflammasome activation (Tavares et al., 2013; Mejía et al., 2015). Consistent with the increased NFC2 level, we have observed an increase in ROS production by macrophages co-cultured with *P. brasiliensis*.

In conclusion, we demonstrated that interaction of human macrophages with *P. brasiliensis* yeast cells resulted in modification of the macrophage proteome, which presented impaired production of proteins participating in energy metabolism, regulation of epigenetic modifications, and gene transcription. Most importantly, data showed repression of many proteins crucial for antifungal immune responses. Although some conclusions are limited and experimental validations are necessary to elucidate the role of the main regulated proteins in macrophages exposed to *P. brasiliensis*, our study contributes to the understanding of the host-pathogen interface, and may provide important information for future studies aiming to develop new therapeutic approaches for PCM.

## Data availability statement

The datasets presented in this study can be found in online repositories. The names of the repository/repositories and accession number(s) can be found in the article/**Supplementary Material**. Data is available at: http://www.ebi.ac.uk/pride/archive/projects/PXD043727.

## Author contributions

AF: Conceptualization, Writing – review & editing, Formal Analysis, Methodology, Validation, Writing – original draft. DM: Methodology, Writing – original draft, Data curation, Software. AB: Conceptualization, Supervision, Writing – review & editing. OR: Writing – review & editing, Methodology. LS: Writing – original draft. FR-D: Conceptualization, Funding acquisition, Project administration, Resources, Supervision, Writing – review & editing. CS: Conceptualization, Supervision, Writing – review & editing, Funding acquisition, Project administration, Resources.

## Supplementary material

Supplementary Spreadsheet S1 All proteins identified in the proteomic analysis.
