# Supplementary material for: Proteomic analysis reveals changes in the proteome of human THP-1 macrophages infected with Paracoccidioides brasiliensis
Source: Front Cell Infect Microbiol. 2023 Nov 16;13:1275954. doi: 10.3389/fcimb.2023.1275954 (PMC10693345; doi:10.3389/fcimb.2023.1275954)
Supplement: Supplementary file 2 [file Table_1.docx]

**Supplementary Table S1 -** Decreased proteins in macrophages co-cultured with *Paracoccidioides*

*brasiliensis* yeast cells.

| Accession number^1^ | Protein description and biological process^2^ | | | Score^3^ | Fold change^4^ |
| --- | --- | --- | --- | --- | --- |
|  | METABOLISM | | |  |  |
|  | **Amino acid metabolism** | | |  |  |
| P23526 | Adenosylhomocysteinase | | | 1908.61 | * |
| P15144 | Aminopeptidase N | | | 6257.54 | * |
| H0YMC1 | Aminopeptidase N (Fragment) | | | 2871.51 | * |
| H0YKT6 | Aminopeptidase N (Fragment) | | | 2445.09 | * |
| Q9NWL6 | Asparagine synthetase domain-containing protein 1 | | | 487.28 | * |
| U3KQK8 | Asparagine synthetase domain-containing protein 1 | | | 487.28 | * |
| C9IYZ1 | Asparagine synthetase domain-containing protein 1 (Fragment) | | | 487.28 | * |
| P54687 | Branched-chain-amino-acid aminotransferase_ cytosolic | | | 1263.97 | * |
| F5H2F2 | Branched-chain-amino-acid aminotransferase_ cytosolic (Fragment) | | | 1238.59 | * |
| A0A087WYF2 | Branched-chain-amino-acid aminotransferase_ cytosolic (Fragment) | | | 299.53 | * |
| G3V4N7 | Creatine kinase (Fragment) | | | 2876.20 | * |
| G3V461 | Creatine kinase (Fragment) | | | 2272.68 | * |
| H0YJG0 | Creatine kinase (Fragment) | | | 279.57 | * |
| P12277 | Creatine kinase B-type | | | 3155.77 | * |
| P28838 | Cytosol aminopeptidase | | | 2459.56 | * |
| H0Y9Q1 | Cytosol aminopeptidase (Fragment) | | | 478.08 | * |
| H0Y983 | Cytosol aminopeptidase (Fragment) | | | 1911.57 | * |
| A0A2R8Y648 | Glutamate--cysteine ligase | | | 396.31 | * |
| G3V540 | Glycine hydroxymethyltransferase | | | 2784.19 | * |
| G3V2E4 | Glycine hydroxymethyltransferase | | | 2623.27 | * |
| G3V2D2 | Glycine hydroxymethyltransferase | | | 354.42 | * |
| G3V5L0 | Glycine hydroxymethyltransferase (Fragment) | | | 3975.53 | * |
| G3V4X0 | Glycine hydroxymethyltransferase (Fragment) | | | 2623.27 | * |
| G3V4W5 | Glycine hydroxymethyltransferase (Fragment) | | | 2784.19 | * |
| G3V4T0 | Glycine hydroxymethyltransferase (Fragment) | | | 2623.27 | * |
| G3V3Y8 | Glycine hydroxymethyltransferase (Fragment) | | | 2623.27 | * |
| G3V2Y4 | Glycine hydroxymethyltransferase (Fragment) | | | 1421.81 | * |
| G3V2W0 | Glycine hydroxymethyltransferase (Fragment) | | | 2784.19 | * |
| H0YIZ0 | Glycine hydroxymethyltransferase (Fragment) | | | 1896.92 | * |
| Q96L73 | Histone-lysine N-methyltransferase_ H3 lysine-36 specific | | | 2554.52 | * |
| D6RE14 | Histone-lysine N-methyltransferase_ H3 lysine-36-specific (Fragment) | | | 2503.04 | * |
| D6RBP3 | Histone-lysine N-methyltransferase_ H3 lysine-36-specific (Fragment) | | | 2503.04 | * |
| D6RA90 | Histone-lysine N-methyltransferase_ H3 lysine-36-specific (Fragment) | | | 2503.04 | * |
| Q6YP21 | Kynurenine--oxoglutarate transaminase 3 | | | 614.25 | * |
| A0A0A0MQU4 | Selenocysteine lyase | | | 1178.25 | * |
| Q96I15 | Selenocysteine lyase | | | 1178.25 | * |
| H7C1N7 | Selenocysteine lyase (Fragment) | | | 480.10 | * |
| H7C4A1 | Selenocysteine lyase (Fragment) | | | 946.82 | * |
| H7C3V9 | Selenocysteine lyase (Fragment) | | | 611.33 | * |
| H7C277 | Selenocysteine lyase (Fragment) | | | 528.47 | * |
| G3V3C6 | Serine hydroxymethyltransferase_ mitochondrial | | | 2068.27 | * |
| G3V2Y1 | Serine hydroxymethyltransferase_ mitochondrial | | | 2068.27 | * |
| P34897 | Serine hydroxymethyltransferase_ mitochondrial | | | 4684.60 | * |
|  | **Nitrogen, sulfur and selenium metabolism** | | |  |  |
| E5RK37 | Carbonic anhydrase 2 | | | 8023.25 | 0.048 |
| P00918 | Carbonic anhydrase 2 | | | 11571.31 | 0.055 |
| E5RID5 | Carbonic anhydrase 2 | | | 8023.25 | 0.063 |
| Q86SX6 | Glutaredoxin-related protein 5_ mitochondrial | | | 1046.90 | * |
| M0QY80 | Persulfide dioxygenase ETHE1_ mitochondrial | | | 5300.99 | * |
| M0QXB5 | Persulfide dioxygenase ETHE1_ mitochondrial | | | 5339.69 | * |
| M0QX80 | Persulfide dioxygenase ETHE1_ mitochondrial | | | 499.82 | * |
| O95571 | Persulfide dioxygenase ETHE1_ mitochondrial | | | 5339.69 | * |
|  | **Nucleotide/nucleoside/nucleobase metabolism** | | |  |  |
| P30520 | Adenylosuccinate synthetase isozyme 2 | | | 1033.35 | * |
| A6NJU6 | ADP-sugar pyrophosphatase | | | 1017.64 | * |
| Q9UKK9 | ADP-sugar pyrophosphatase | | | 1364.46 | * |
| A6NFX8 | ADP-sugar pyrophosphatase | | | 1364.46 | * |
| A6NCQ0 | ADP-sugar pyrophosphatase | | | 916.59 | * |
| H0YEY4 | ADP-sugar pyrophosphatase (Fragment) | | | 586.47 | * |
| F8WEF0 | AICAR transformylase/inosine monophosphate cyclohydrolase | | | 4858.63 | * |
| C9JLK0 | AICAR transformylase/inosine monophosphate cyclohydrolase (Fragment) | | | 4427.27 | * |
| D6RF62 | AIR carboxylase | | | 1750.27 | * |
| E9PBS1 | AIR carboxylase (Fragment) | | | 1769.69 | * |
| P31939 | Bifunctional purine biosynthesis protein ATIC | | | 13173.63 | * |
| H7C1S2 | Bifunctional purine biosynthesis protein ATIC (Fragment) | | | 2634.33 | * |
| H0YNW5 | Deoxyuridine 5'-triphosphate nucleotidohydrolase | | | 5566.70 | * |
| H0YNJ9 | Deoxyuridine 5'-triphosphate nucleotidohydrolase | | | 238.68 | * |
| H0YMM5 | Deoxyuridine 5'-triphosphate nucleotidohydrolase | | | 238.68 | * |
| H0YKI0 | Deoxyuridine 5'-triphosphate nucleotidohydrolase | | | 270.24 | * |
| A0A0C4DGL3 | Deoxyuridine 5'-triphosphate nucleotidohydrolase | | | 5506.38 | * |
| H0YMP1 | Deoxyuridine 5'-triphosphate nucleotidohydrolase (Fragment) | | | 447.40 | * |
| H0YKC5 | Deoxyuridine 5'-triphosphate nucleotidohydrolase (Fragment) | | | 5566.70 | * |
| P33316 | Deoxyuridine 5'-triphosphate nucleotidohydrolase_ mitochondrial | | | 5598.26 | * |
| F6XY72 | HCG2001850_ isoform CRA_c | | | 9908.49 | * |
| A0A6Q8PGQ8 | Histidine triad nucleotide-binding protein 1 | | | 2775.44 | * |
| D6REP8 | Histidine triad nucleotide-binding protein 1 | | | 2775.44 | * |
| D6RE99 | Histidine triad nucleotide-binding protein 1 | | | 2775.44 | * |
| D6RD60 | Histidine triad nucleotide-binding protein 1 | | | 2775.44 | * |
| D6RC06 | Histidine triad nucleotide-binding protein 1 | | | 974.47 | * |
| H0YC49 | Histidine triad nucleotide-binding protein 1 | | | 2775.44 | * |
| P49773 | Histidine triad nucleotide-binding protein 1 | | | 2950.50 | * |
| P00492 | Hypoxanthine-guanine phosphoribosyltransferase | | | 735.80 | * |
| A0A7I2YQK5 | Inosine-5'-monophosphate dehydrogenase | | | 1242.72 | * |
| E7ETK5 | Inosine-5'-monophosphate dehydrogenase | | | 1206.09 | * |
| H0Y4R1 | Inosine-5'-monophosphate dehydrogenase | | | 1242.72 | * |
| A0A7I2V2T3 | Inosine-5'-monophosphate dehydrogenase | | | 1206.09 | * |
| P12268 | Inosine-5'-monophosphate dehydrogenase 2 | | | 1206.09 | * |
| G3V5M2 | Inosine-guanosine phosphorylase (Fragment) | | | 11039.80 | * |
| G3V393 | Inosine-guanosine phosphorylase (Fragment) | | | 447.44 | * |
| P22234 | Multifunctional protein ADE2 | | | 1769.69 | * |
| J3KPD9 | NME1-NME2 readthrough | | | 10461.85 | * |
| E7ERL0 | Nucleoside diphosphate kinase A | | | 10461.85 | * |
| P15531 | Nucleoside diphosphate kinase A | | | 10461.85 | * |
| E5RHP0 | Nucleoside diphosphate kinase A | | | 9914.65 | * |
| C9K028 | Nucleoside diphosphate kinase A (Fragment) | | | 9908.49 | * |
| P00491 | Purine nucleoside phosphorylase | | | 16212.25 | * |
| G3V2H3 | Purine nucleoside phosphorylase (Fragment) | | | 8696.56 | * |
|  | **C-compound and carbohydrate metabolism** | | |  |  |
| E9PEF9 | Aldo-keto reductase family 1 member B1 | | | 230.12 | * |
| P11766 | Alcohol dehydrogenase class-3 | | | 5335.49 | * |
| D6RFE4 | Alcohol dehydrogenase class-3 | | | 4615.11 | * |
| D6RAY0 | Alcohol dehydrogenase class-3 | | | 1246.48 | * |
| D6R9G2 | Alcohol dehydrogenase class-3 | | | 1246.48 | * |
| H0YAG8 | Alcohol dehydrogenase class-3 (Fragment) | | | 1633.85 | * |
| E9PK47 | Alpha-1_4 glucan phosphorylase | | | 1890.55 | * |
| H0Y4Z6 | Alpha-1_4 glucan phosphorylase (Fragment) | | | 1299.96 | * |
| P54802 | Alpha-N-acetylglucosaminidase | | | 531.40 | * |
| H3BS10 | Beta-hexosaminidase | | | 329.90 | * |
| H3BP20 | Beta-hexosaminidase | | | 364.53 | * |
| P06865 | Beta-hexosaminidase subunit alpha | | | 364.53 | * |
| P07686 | Beta-hexosaminidase subunit beta | | | 1363.17 | * |
| H3BTD4 | Beta-N-acetylhexosaminidase | | | 303.71 | * |
| Q5URX0 | Beta-N-acetylhexosaminidase | | | 1359.97 | * |
| H0Y9B6 | Beta-N-acetylhexosaminidase (Fragment) | | | 902.04 | * |
| H3BU85 | Beta-N-acetylhexosaminidase (Fragment) | | | 277.32 | * |
| E9PKL9 | GDP-4-keto-6-deoxy-D-mannose-3_5-epimerase-4-reductase (Fragment) | | | 2024.21 | * |
| E9PP60 | GDP-L-fucose synthase | | | 1518.13 | * |
| Q13630 | GDP-L-fucose synthase | | | 2037.99 | * |
| A0A0J9YX13 | GDP-L-fucose synthase (Fragment) | | | 1830.02 | * |
| E9PP14 | GDP-L-fucose synthase (Fragment) | | | 1830.02 | * |
| E9PLH9 | GDP-L-fucose synthase (Fragment) | | | 1751.40 | * |
| P11413 | Glucose-6-phosphate 1-dehydrogenase | | | 1506.53 | * |
| E7EUI8 | Glucose-6-phosphate 1-dehydrogenase (Fragment) | | | 1402.54 | * |
| E9PD92 | Glucose-6-phosphate 1-dehydrogenase (Fragment) | | | 1421.07 | * |
| E7EM57 | Glucose-6-phosphate 1-dehydrogenase (Fragment) | | | 1400.11 | * |
| P11216 | Glycogen phosphorylase_ brain form | | | 2313.76 | 0.051 |
| P06737 | Glycogen phosphorylase_ liver form | | | 1890.55 | * |
| P11217 | Glycogen phosphorylase_ muscle form | | | 8381.66 | 0.477 |
| Q9UBQ7 | Glyoxylate reductase/hydroxypyruvate reductase | | | 1690.10 | * |
| U3KQ56 | Glyoxylate reductase/hydroxypyruvate reductase | | | 1499.36 | * |
| Q04760 | Lactoylglutathione lyase | | | 13189.76 | * |
| F5H245 | L-lactate dehydrogenase | | | 748.34 | * |
| F5H155 | L-lactate dehydrogenase | | | 716.46 | * |
| A0A3B3IS95 | L-lactate dehydrogenase | | | 37915.12 | * |
| G3XAP5 | L-lactate dehydrogenase | | | 716.46 | * |
| A0A5F9ZHM4 | L-lactate dehydrogenase | | | 41051.41 | * |
| A0A087WUM2 | L-lactate dehydrogenase | | | 696.97 | * |
| F5H5G7 | L-lactate dehydrogenase | | | 716.46 | * |
| A8MW50 | L-lactate dehydrogenase (Fragment) | | | 37537.77 | * |
| P00338 | L-lactate dehydrogenase A chain | | | 13796.04 | * |
| F5GXU1 | L-lactate dehydrogenase A chain | | | 5368.27 | * |
| F5GXC7 | L-lactate dehydrogenase A chain | | | 5368.27 | * |
| F5GWW2 | L-lactate dehydrogenase A chain | | | 5368.27 | * |
| F5H8H6 | L-lactate dehydrogenase A chain | | | 5368.27 | * |
| F5H6W8 | L-lactate dehydrogenase A chain | | | 8552.99 | * |
| F5H5J4 | L-lactate dehydrogenase A chain | | | 8552.99 | * |
| F5GZQ4 | L-lactate dehydrogenase A chain (Fragment) | | | 7824.08 | * |
| F5GYU2 | L-lactate dehydrogenase A chain (Fragment) | | | 11111.23 | * |
| F5GXY2 | L-lactate dehydrogenase A chain (Fragment) | | | 11111.23 | * |
| F5GXH2 | L-lactate dehydrogenase A chain (Fragment) | | | 10241.03 | * |
| Q6ZMR3 | L-lactate dehydrogenase A-like 6A | | | 703.09 | * |
| Q9BYZ2 | L-lactate dehydrogenase A-like 6B | | | 914.21 | * |
| P07195 | L-lactate dehydrogenase B chain | | | 41051.41 | * |
| F5H793 | L-lactate dehydrogenase B chain (Fragment) | | | 35403.22 | * |
| C9J7H8 | L-lactate dehydrogenase B chain (Fragment) | | | 36840.80 | * |
| P07864 | L-lactate dehydrogenase C chain | | | 748.34 | * |
| O00754 | Lysosomal alpha-mannosidase | | | 7741.12 | * |
| M0R174 | Lysosomal alpha-mannosidase (Fragment) | | | 270.58 | * |
| H0YFA9 | N-acetylglucosamine-6-sulfatase (Fragment) | | | 461.73 | * |
| F5H4C6 | N-acetylglucosamine-6-sulfatase (Fragment) | | | 362.01 | * |
| Q9NR45 | Sialic acid synthase | | | 1300.14 | * |
| Q5TBR1 | Sialic acid synthase (Fragment) | | | 903.99 | * |
| Q5TBR0 | Sialic acid synthase (Fragment) | | | 903.99 | * |
| P37837 | Transaldolase | | | 4074.58 | * |
| F2Z393 | Transaldolase | | | 4074.58 | * |
| E9PM01 | Transaldolase | | | 511.68 | * |
| E9PKI8 | Transaldolase | | | 511.68 | * |
| A0A0B4J1R6 | Transketolase | | | 7867.23 | * |
| P29401 | Transketolase | | | 7913.89 | * |
| E9PFF2 | Transketolase | | | 5784.93 | * |
| F8W888 | Transketolase | | | 1493.76 | * |
|  | **Lipid,fatty acid and isoprenoid metabolism** | | |  |  |
| P42765 | 3-ketoacyl-CoA thiolase_ mitochondrial | | | 3771.81 | * |
| K7EME0 | 3-ketoacyl-CoA thiolase_ mitochondrial | | | 3617.26 | * |
| A0A0B4J2A4 | 3-ketoacyl-CoA thiolase_ mitochondrial | | | 3678.89 | * |
| A0A5F9ZHL7 | Acetyl-CoA acetyltransferase_ mitochondrial | | | 1367.16 | * |
| E9PRQ6 | Acetyl-CoA acetyltransferase_ mitochondrial | | | 1367.16 | * |
| A0A1B0GUE3 | Acid ceramidase | | | 1092.41 | * |
| A0A1B0GTP7 | Acid ceramidase | | | 1092.41 | * |
| Q13510 | Acid ceramidase | | | 1092.41 | * |
| A0A1B0GUW4 | Acid ceramidase (Fragment) | | | 1092.41 | * |
| A0A1B0GV06 | Acid ceramidase (Fragment) | | | 1092.41 | * |
| A0A0A0MTI5 | Acyl-CoA-binding protein | | | 9198.68 | * |
| P07108 | Acyl-CoA-binding protein | | | 9198.68 | * |
| B8ZWD1 | Acyl-CoA-binding protein | | | 9198.68 | * |
| G3V4F2 | Acyl-coenzyme A thioesterase 1 | | | 765.06 | * |
| Q86TX2 | Acyl-coenzyme A thioesterase 1 | | | 773.74 | * |
| A0A087X0W7 | Acyl-coenzyme A thioesterase 2_ mitochondrial | | | 773.74 | * |
| P49753 | Acyl-coenzyme A thioesterase 2_ mitochondrial | | | 773.74 | * |
| A0A087WT95 | Acyl-coenzyme A thioesterase 2_ mitochondrial | | | 773.74 | * |
| P16152 | Carbonyl reductase [NADPH] 1 | | | 675.19 | * |
| E7EMM4 | Ceramidase | | | 1092.41 | * |
| A0A1B0GW68 | Ceramidase | | | 1092.41 | * |
| A0A1B0GUH5 | Ceramidase | | | 1092.41 | * |
| A0A1B0GUG1 | Ceramidase | | | 1092.41 | * |
| A0A1B0GUA4 | Ceramidase | | | 1092.41 | * |
| A0A1B0GTZ5 | Ceramidase | | | 1092.41 | * |
| A0A1B0GTM3 | Ceramidase | | | 1092.41 | * |
| I6L8B7 | Fatty acid-binding protein 5 | | | 7791.91 | * |
| Q01469 | Fatty acid-binding protein 5 | | | 9854.93 | * |
| Q16836 | Hydroxyacyl-coenzyme A dehydrogenase_ mitochondrial | | | 1448.48 | * |
| A0A1W2PQV5 | Hydroxyacyl-coenzyme A dehydrogenase_ mitochondrial | | | 1405.19 | * |
| A0A1W2PQ78 | Hydroxyacyl-coenzyme A dehydrogenase_ mitochondrial | | | 1448.48 | * |
| E9PF18 | Hydroxyacyl-coenzyme A dehydrogenase_ mitochondrial | | | 1449.22 | * |
| A0A0A0MSE2 | Hydroxyacyl-coenzyme A dehydrogenase_ mitochondrial | | | 1448.48 | * |
| A0A0D9SFP2 | Hydroxyacyl-coenzyme A dehydrogenase_ mitochondrial | | | 1448.48 | * |
| A0A1W2PRT2 | Hydroxyacyl-coenzyme A dehydrogenase_ mitochondrial (Fragment) | | | 1123.42 | * |
| A0A1W2PQC2 | Hydroxyacyl-coenzyme A dehydrogenase_ mitochondrial (Fragment) | | | 1123.42 | * |
| A0A1W2PNM1 | Hydroxyacyl-coenzyme A dehydrogenase_ mitochondrial (Fragment) | | | 1448.48 | * |
| Q13907 | Isopentenyl-diphosphate Delta-isomerase 1 | | | 1200.99 | * |
| Q9BXS1 | Isopentenyl-diphosphate delta-isomerase 2 | | | 197.42 | * |
| P09960 | Leukotriene A-4 hydrolase | | | 1585.24 | * |
| B4DEH5 | Leukotriene A-4 hydrolase | | | 593.71 | * |
| A0A7P0TB55 | Medium-chain-specific acyl-CoA dehydrogenase_ mitochondrial | | | 5740.52 | * |
| A0A7P0T8G6 | Medium-chain-specific acyl-CoA dehydrogenase_ mitochondrial | | | 6113.67 | * |
| H0YDT5 | Medium-chain-specific acyl-CoA dehydrogenase_ mitochondrial (Fragment) | | | 3954.60 | * |
| A0A286YFF7 | Palmitoyl-protein hydrolase 1 | | | 1865.90 | * |
| A0A286YFE3 | Palmitoyl-protein hydrolase 1 | | | 1279.54 | * |
| E9PSE5 | Palmitoyl-protein hydrolase 1 | | | 586.37 | * |
| Q5T0S4 | Palmitoyl-protein hydrolase 1 | | | 1279.54 | * |
| A0A2C9F2P4 | Palmitoyl-protein hydrolase 1 | | | 1865.90 | * |
| E9PIA8 | Palmitoyl-protein hydrolase 1 (Fragment) | | | 658.28 | * |
| A0A286YFL8 | Palmitoyl-protein hydrolase 1 (Fragment) | | | 1279.54 | * |
| P50897 | Palmitoyl-protein thioesterase 1 | | | 1865.90 | * |
| A0A286YFL6 | Palmitoyl-protein thioesterase 1 (Fragment) | | | 610.74 | * |
| C9JIZ6 | Prosaposin | | | 6066.00 | * |
| P07602 | Prosaposin | | | 6066.00 | * |
| A0A0J9YXB8 | Prosaposin (Fragment) | | | 2984.44 | * |
| B4DDC6 | Prostaglandin E synthase 3 | | | 1087.05 | * |
| Q15185 | Prostaglandin E synthase 3 | | | 1656.95 | * |
| A0A087WYT3 | Prostaglandin E synthase 3 | | | 1656.95 | * |
| Q5BJH1 | PSAP protein | | | 2895.48 | * |
| P22307 | Sterol carrier protein 2 | | | 1405.82 | * |
| E9PLD1 | Sterol carrier protein 2 | | | 539.28 | * |
| H0YF61 | Sterol carrier protein 2 (Fragment) | | | 752.84 | * |
| H0YD06 | Sterol carrier protein 2 (Fragment) | | | 309.13 | * |
| H0YCB0 | Sterol carrier protein 2 (Fragment) | | | 508.54 | * |
| Metabolism of vitamins, cofactors, and prosthetic groups | | | | | |
| F5H2F4 | C-1-tetrahydrofolate synthase_ cytoplasmic | | | 700.59 | * |
| P11586 | C-1-tetrahydrofolate synthase_ cytoplasmic | | | 694.05 | * |
| V9GYY3 | C-1-tetrahydrofolate synthase_ cytoplasmic | | | 694.05 | * |
| V9GZ78 | C-1-tetrahydrofolate synthase_ cytoplasmic | | | 662.73 | * |
| A0A494C1T2 | C-1-tetrahydrofolate synthase_ cytoplasmic (Fragment) | | | 578.33 | * |
| Q16719 | Kynureninase | | | 721.29 | * |
| A0A087WYQ7 | Kynureninase (Fragment) | | | 578.00 | * |
| A0A087WYM2 | Kynureninase (Fragment) | | | 578.00 | * |
| A0A087X297 | Kynureninase (Fragment) | | | 604.01 | * |
| H3BRK3 | NAD(P)H dehydrogenase [quinone] 1 | | | 664.12 | * |
| B4DLR8 | NAD(P)H dehydrogenase [quinone] 1 | | | 1058.66 | * |
| H3BNV2 | NAD(P)H dehydrogenase [quinone] 1 | | | 757.96 | * |
| P15559 | NAD(P)H dehydrogenase [quinone] 1 | | | 1058.66 | * |
| Q15274 | Nicotinate-nucleotide pyrophosphorylase [carboxylating] | | | 610.12 | * |
| Q9BZ23 | Pantothenate kinase 2_ mitochondrial | | | 999.07 | * |
| V9GYZ0 | Pantothenate kinase 2_ mitochondrial | | | 963.73 | * |
| J3QQZ9 | Pyridoxal 5'-phosphate synthase (Fragment) | | | 782.37 | * |
| A0A286YFL3 | Pyridoxal 5'-phosphate synthase (Fragment) | | | 350.61 | * |
| F2Z2Y4 | Pyridoxal kinase | | | 2141.53 | * |
| O00764 | Pyridoxal kinase | | | 2186.82 | * |
| Q9NVS9 | Pyridoxine-5'-phosphate oxidase | | | 1028.84 | * |
|  | ENERGY | | |  |  |
|  | **Glycolysis and Gluconeogenesis** | | |  |  |
| E5RGZ4 | 2-phospho-D-glycerate hydro-lyase | | | 5340.21 | * |
| F5H0C8 | 2-phospho-D-glycerate hydro-lyase | | | 5297.14 | * |
| A0A2R8Y6G6 | 2-phospho-D-glycerate hydro-lyase | | | 23270.41 | * |
| K7EPM1 | 2-phospho-D-glycerate hydro-lyase (Fragment) | | | 4580.19 | * |
| K7EM90 | 2-phospho-D-glycerate hydro-lyase (Fragment) | | | 52315.97 | * |
| K7EKN2 | 2-phospho-D-glycerate hydro-lyase (Fragment) | | | 4580.19 | * |
| U3KQP4 | 2-phospho-D-glycerate hydro-lyase (Fragment) | | | 760.02 | * |
| A0A2R8YEM5 | 2-phospho-D-glycerate hydro-lyase (Fragment) | | | 52029.39 | * |
| A0A2R8Y798 | Alpha-enolase | | | 4990.68 | * |
| P06733 | Alpha-enolase | | | 63037.82 | * |
| A0A2R8YEG5 | Alpha-enolase | | | 4990.68 | * |
| K7ERS8 | Alpha-enolase (Fragment) | | | 44758.10 | * |
| A0A2R8Y879 | Alpha-enolase (Fragment) | | | 51173.03 | * |
| A0A2R8Y6I8 | Alpha-enolase (Fragment) | | | 46182.34 | * |
| A0A7I2V3Z0 | ATP-dependent 6-phosphofructokinase | | | 6534.87 | * |
| P17858 | ATP-dependent 6-phosphofructokinase_ liver type | | | 670.40 | * |
| F8VZI0 | ATP-dependent 6-phosphofructokinase_ muscle type | | | 416.68 | * |
| A0A7I2YQB6 | ATP-dependent 6-phosphofructokinase_ platelet type | | | 367.55 | * |
| B1APP6 | ATP-dependent 6-phosphofructokinase_ platelet type | | | 3879.28 | * |
| A0A7I2V5S1 | ATP-dependent 6-phosphofructokinase_ platelet type | | | 2639.54 | * |
| Q01813 | ATP-dependent 6-phosphofructokinase_ platelet type | | | 6601.00 | * |
| H0Y757 | ATP-dependent 6-phosphofructokinase_ platelet type (Fragment) | | | 2008.59 | * |
| H0Y3Y3 | ATP-dependent 6-phosphofructokinase_ platelet type (Fragment) | | | 523.35 | * |
| V9GYV7 | ATP-dependent 6-phosphofructokinase_ platelet type (Fragment) | | | 2639.54 | * |
| V9GY25 | ATP-dependent 6-phosphofructokinase_ platelet type (Fragment) | | | 989.68 | * |
| Q5VSR5 | ATP-dependent 6-phosphofructokinase_ platelet type (Fragment) | | | 2655.59 | * |
| P13929 | Beta-enolase | | | 5425.63 | * |
| E5RG95 | Beta-enolase (Fragment) | | | 4580.19 | * |
| E5RI09 | Beta-enolase (Fragment) | | | 4580.19 | * |
| A0A3B3IS80 | Fructose-bisphosphate aldolase | | | 1668.83 | * |
| H3BQN4 | Fructose-bisphosphate aldolase | | | 37895.93 | * |
| J3KPS3 | Fructose-bisphosphate aldolase | | | 31785.80 | * |
| A8MVZ9 | Fructose-bisphosphate aldolase | | | 2940.52 | * |
| A0A087WXX2 | Fructose-bisphosphate aldolase | | | 1648.60 | * |
| H3BUH7 | Fructose-bisphosphate aldolase (Fragment) | | | 20789.33 | * |
| H3BU78 | Fructose-bisphosphate aldolase (Fragment) | | | 23463.60 | * |
| J3KSV6 | Fructose-bisphosphate aldolase (Fragment) | | | 2901.11 | * |
| H3BR68 | Fructose-bisphosphate aldolase (Fragment) | | | 7206.79 | * |
| J3QKP5 | Fructose-bisphosphate aldolase (Fragment) | | | 2901.11 | * |
| H3BR04 | Fructose-bisphosphate aldolase (Fragment) | | | 22886.86 | * |
| J3QKK1 | Fructose-bisphosphate aldolase (Fragment) | | | 939.71 | * |
| H3BPS8 | Fructose-bisphosphate aldolase (Fragment) | | | 31511.15 | * |
| H3BMQ8 | Fructose-bisphosphate aldolase (Fragment) | | | 20783.38 | * |
| K7EKH5 | Fructose-bisphosphate aldolase (Fragment) | | | 2580.11 | * |
| C9J8F3 | Fructose-bisphosphate aldolase (Fragment) | | | 2864.43 | * |
| P04075 | Fructose-bisphosphate aldolase A | | | 39329.30 | * |
| P05062 | Fructose-bisphosphate aldolase B | | | 1668.83 | * |
| P09972 | Fructose-bisphosphate aldolase C | | | 12130.18 | * |
| P09104 | Gamma-enolase | | | 5319.81 | * |
| F5H1C3 | Gamma-enolase (Fragment) | | | 4469.53 | * |
| A0A2U3TZU2 | Glucose-6-phosphate isomerase | | | 3765.44 | 0.394 |
| A0A2R8Y6C7 | Glucose-6-phosphate isomerase | | | 3663.13 | 0.427 |
| P06744 | Glucose-6-phosphate isomerase | | | 3765.44 | 0.402 |
| K7EPY4 | Glucose-6-phosphate isomerase (Fragment) | | | 1624.04 | 0.427 |
| K7EQ48 | Glucose-6-phosphate isomerase (Fragment) | | | 3765.44 | 0.032 |
| K7EP41 | Glucose-6-phosphate isomerase (Fragment) | | | 1286.43 | 0.543 |
| K7ENA0 | Glucose-6-phosphate isomerase (Fragment) | | | 348.13 | 0.172 |
| A0A0J9YYH3 | Glucose-6-phosphate isomerase (Fragment) | | | 3663.13 | 0.310 |
| A0A0J9YXP8 | Glucose-6-phosphate isomerase (Fragment) | | | 3663.13 | 0.382 |
| A0A0J9YX90 | Glucose-6-phosphate isomerase (Fragment) | | | 3663.13 | 0.371 |
| E7EUT5 | Glyceraldehyde-3-phosphate dehydrogenase | | | 17109.46 | * |
| P04406 | Glyceraldehyde-3-phosphate dehydrogenase | | | 21535.55 | * |
| O14556 | Glyceraldehyde-3-phosphate dehydrogenase_ testis-specific | | | 399.52 | * |
| P00558 | Phosphoglycerate kinase 1 | | | 23595.51 | * |
| P07205 | Phosphoglycerate kinase 2 | | | 1115.35 | * |
| P18669 | Phosphoglycerate mutase 1 | | | 26342.06 | * |
| P15259 | Phosphoglycerate mutase 2 | | | 17441.32 | * |
| Q8N0Y7 | Probable phosphoglycerate mutase 4 | | | 13757.52 | * |
| H3BQ34 | Pyruvate kinase | | | 28439.04 | * |
| H3BUW1 | Pyruvate kinase (Fragment) | | | 22386.56 | * |
| H3BU13 | Pyruvate kinase (Fragment) | | | 14905.15 | * |
| H3BTN5 | Pyruvate kinase (Fragment) | | | 42200.94 | * |
| H3BTJ2 | Pyruvate kinase (Fragment) | | | 22386.56 | * |
| H3BT25 | Pyruvate kinase (Fragment) | | | 22386.56 | * |
| P30613 | Pyruvate kinase PKLR | | | 1599.20 | * |
| H3BQZ3 | Pyruvate kinase PKM | | | 14882.46 | * |
| P14618 | Pyruvate kinase PKM | | | 47876.48 | 0.132 |
| H3BN34 | Pyruvate kinase PKM (Fragment) | | | 14882.46 | * |
| P60174 | Triosephosphate isomerase | | | 35540.98 | 0.379 |
| U3KQF3 | Triosephosphate isomerase (Fragment) | | | 22350.00 | * |
| U3KPZ0 | Triosephosphate isomerase (Fragment) | | | 29255.70 | 0.166 |
| U3KPS5 | Triosephosphate isomerase (Fragment) | | | 15227.33 | * |
|  | **Tricarboxylic-acid cycle** | | |  |  |
| A2A274 | Aconitate hydratase_ mitochondrial | | | 916.17 | * |
| A0A7I2V5W7 | Aconitate hydratase_ mitochondrial | | | 654.06 | * |
| A0A7I2V5T4 | Aconitate hydratase_ mitochondrial | | | 910.17 | * |
| A0A7I2V614 | Aconitate hydratase_ mitochondrial | | | 883.83 | * |
| A0A7I2V5A1 | Aconitate hydratase_ mitochondrial | | | 605.06 | * |
| A0A7I2V586 | Aconitate hydratase_ mitochondrial | | | 916.17 | * |
| A0A7I2V538 | Aconitate hydratase_ mitochondrial | | | 390.66 | * |
| A0A7I2V4I8 | Aconitate hydratase_ mitochondrial | | | 654.06 | * |
| A0A7I2V3U0 | Aconitate hydratase_ mitochondrial | | | 916.17 | * |
| A0A7I2V3F1 | Aconitate hydratase_ mitochondrial | | | 916.17 | * |
| A0A7I2V3C8 | Aconitate hydratase_ mitochondrial | | | 654.06 | * |
| Q99798 | Aconitate hydratase_ mitochondrial | | | 916.17 | * |
| A0A7I2V3U7 | Aconitate hydratase_ mitochondrial (Fragment) | | | 574.36 | * |
| P53396 | ATP-citrate synthase | | | 1520.56 | * |
| K7ESG8 | ATP-citrate synthase (Fragment) | | | 1353.50 | * |
| B4DJV2 | Citrate synthase | | | 12200.29 | * |
| A0A0C4DGI3 | Citrate synthase | | | 11319.46 | * |
| H0YIC4 | Citrate synthase (Fragment) | | | 374.11 | * |
| O75390 | Citrate synthase_ mitochondrial | | | 12200.29 | * |
| F8W0J2 | Citrate synthase_ mitochondrial | | | 880.83 | * |
| F8VWQ5 | Citrate synthase_ mitochondrial | | | 880.83 | * |
| F8VRP1 | Citrate synthase_ mitochondrial (Fragment) | | | 9896.84 | * |
| F8VRI6 | Citrate synthase_ mitochondrial (Fragment) | | | 239.02 | * |
| F8VR34 | Citrate synthase_ mitochondrial (Fragment) | | | 898.15 | * |
| F8W1S4 | Citrate synthase_ mitochondrial (Fragment) | | | 9896.84 | * |
| F8VPF9 | Citrate synthase_ mitochondrial (Fragment) | | | 9896.84 | * |
| F8VPA1 | Citrate synthase_ mitochondrial (Fragment) | | | 9914.16 | * |
| H0YH82 | Citrate synthase_ mitochondrial (Fragment) | | | 9896.84 | * |
| F8VZK9 | Citrate synthase_ mitochondrial (Fragment) | | | 9896.84 | * |
| F8VX68 | Citrate synthase_ mitochondrial (Fragment) | | | 9896.84 | * |
| F8VX07 | Citrate synthase_ mitochondrial (Fragment) | | | 9896.84 | * |
| F8VTT8 | Citrate synthase_ mitochondrial (Fragment) | | | 10777.66 | * |
| F8W642 | Citrate synthase_ mitochondrial (Fragment) | | | 9896.84 | * |
| F8VU34 | Citrate synthase_ mitochondrial (Fragment) | | | 880.83 | * |
| F8W4S1 | Citrate synthase_ mitochondrial (Fragment) | | | 10777.66 | * |
| C9JLV6 | Cytosolic malate dehydrogenase (Fragment) | | | 414.12 | * |
| C9JRL4 | Cytosolic malate dehydrogenase (Fragment) | | | 13862.17 | * |
| E9PEX6 | Dihydrolipoyl dehydrogenase | | | 1623.05 | * |
| P09622 | Dihydrolipoyl dehydrogenase_ mitochondrial | | | 1686.78 | * |
| A0A1W2PR83 | Dihydrolipoyl dehydrogenase_ mitochondrial | | | 1018.21 | * |
| F8WDM5 | Dihydrolipoyl dehydrogenase_ mitochondrial | | | 1018.21 | * |
| F2Z2E3 | Dihydrolipoyl dehydrogenase_ mitochondrial | | | 1018.21 | * |
| P07954 | Fumarate hydratase_ mitochondrial | | | 2669.02 | * |
| H0YNF5 | Isocitrate dehydrogenase [NAD] subunit alpha_ mitochondrial | | | 992.73 | * |
| P50213 | Isocitrate dehydrogenase [NAD] subunit alpha_ mitochondrial | | | 1918.82 | * |
| H0YL72 | Isocitrate dehydrogenase [NAD] subunit alpha_ mitochondrial | | | 1918.82 | * |
| H0YMU3 | Isocitrate dehydrogenase [NAD] subunit alpha_ mitochondrial (Fragment) | | | 891.62 | * |
| H0YM64 | Isocitrate dehydrogenase [NAD] subunit alpha_ mitochondrial (Fragment) | | | 671.34 | * |
| H0YM46 | Isocitrate dehydrogenase [NAD] subunit alpha_ mitochondrial (Fragment) | | | 992.73 | * |
| H0YLI6 | Isocitrate dehydrogenase [NAD] subunit alpha_ mitochondrial (Fragment) | | | 891.62 | * |
| H0YKD0 | Isocitrate dehydrogenase [NAD] subunit alpha_ mitochondrial (Fragment) | | | 809.32 | * |
| O75874 | Isocitrate dehydrogenase [NADP] cytoplasmic | | | 975.87 | * |
| C9J4N6 | Isocitrate dehydrogenase [NADP] cytoplasmic (Fragment) | | | 830.55 | * |
| A0A5K1VW95 | Malate dehydrogenase | | | 15614.68 | * |
| C9JF79 | Malate dehydrogenase (Fragment) | | | 13862.17 | * |
| B9A041 | Malate dehydrogenase_ cytoplasmic | | | 18794.44 | * |
| P40925 | Malate dehydrogenase_ cytoplasmic | | | 19208.56 | * |
| B8ZZ51 | Malate dehydrogenase_ cytoplasmic | | | 5346.39 | * |
| P53597 | Succinate--CoA ligase [ADP/GDP-forming] subunit alpha_ mitochondrial | | | 651.86 | * |
| A0A494C0D1 | Succinate--CoA ligase [ADP/GDP-forming] subunit alpha_ mitochondrial | | | 613.11 | * |
| Electron transport and membrane-associated energy conservation | | | | | |
| K7EQH4 | ATP synthase subunit alpha_ mitochondrial (Fragment) | | | 17155.48 | * |
| K7EJP1 | ATP synthase subunit alpha_ mitochondrial (Fragment) | | | 17166.89 | * |
| H0YH81 | ATP synthase subunit beta (Fragment) | | | 18020.49 | * |
| P06576 | ATP synthase subunit beta_ mitochondrial | | | 18491.35 | * |
| F8W0P7 | ATP synthase subunit beta_ mitochondrial (Fragment) | | | 7800.63 | * |
| F8W079 | ATP synthase subunit beta_ mitochondrial (Fragment) | | | 8822.12 | * |
| H0YI37 | ATP synthase subunit beta_ mitochondrial (Fragment) | | | 6106.11 | * |
| P36542 | ATP synthase subunit gamma_ mitochondrial | | | 1169.11 | * |
| A8MUH2 | ATP synthase-coupling factor 6_ mitochondrial | | | 1566.69 | * |
| P18859 | ATP synthase-coupling factor 6_ mitochondrial | | | 1629.49 | * |
| Q14061 | Cytochrome c oxidase copper chaperone | | | 7210.97 | * |
| C9J8T6 | Cytochrome c oxidase copper chaperone | | | 7210.97 | * |
| H0YK49 | Electron transfer flavoprotein subunit alpha | | | 1256.19 | * |
| H0YL83 | Electron transfer flavoprotein subunit alpha_ mitochondrial | | | 629.47 | * |
| P13804 | Electron transfer flavoprotein subunit alpha_ mitochondrial | | | 1834.68 | * |
| H0YNX6 | Electron transfer flavoprotein subunit alpha_ mitochondrial (Fragment) | | | 812.76 | * |
| H0YLU7 | Electron transfer flavoprotein subunit alpha_ mitochondrial (Fragment) | | | 1382.31 | * |
| H0YL12 | Electron transfer flavoprotein subunit alpha_ mitochondrial (Fragment) | | | 967.74 | * |
| H0YKF0 | Electron transfer flavoprotein subunit alpha_ mitochondrial (Fragment) | | | 834.82 | * |
| Q5SQT6 | Inorganic diphosphatase | | | 403.07 | * |
| H0Y9D8 | Inorganic diphosphatase (Fragment) | | | 1522.39 | * |
| D6RGV9 | Inorganic diphosphatase (Fragment) | | | 982.85 | * |
| D6RGI1 | Inorganic diphosphatase (Fragment) | | | 397.24 | * |
| H0YAK2 | Inorganic diphosphatase (Fragment) | | | 350.28 | * |
| D6R967 | Inorganic diphosphatase (Fragment) | | | 1183.94 | * |
| Q15181 | Inorganic pyrophosphatase | | | 721.87 | * |
| Q9H2U2 | Inorganic pyrophosphatase 2_ mitochondrial | | | 1668.87 | * |
| O75323 | Protein NipSnap homolog 2 | | | 2351.76 | * |
| F8WBI5 | Protein NipSnap homolog 2 | | | 634.52 | * |
| C9K068 | Protein NipSnap homolog 2 (Fragment) | | | 1663.53 | * |
| H7C333 | Protein NipSnap homolog 2 (Fragment) | | | 1663.53 | * |
| C9J7B1 | Protein NipSnap homolog 2 (Fragment) | | | 1663.53 | * |
| Q93050 | V-type proton ATPase 116 kDa subunit a1 | | | 2050.49 | * |
| K7EPG4 | V-type proton ATPase subunit a (Fragment) | | | 2018.01 | * |
| K7EN36 | V-type proton ATPase subunit a (Fragment) | | | 1994.40 | * |
| K7EM24 | V-type proton ATPase subunit a (Fragment) | | | 2007.44 | * |
|  | CELL CYCLE AND DNA PROCESSING | | |  |  |
|  | **DNA processing** | | |  |  |
| B1AHC9 | ATP-dependent DNA helicase 2 subunit 1 | | | 420.34 | * |
| A0A7P0TB30 | DNA damage-binding protein 1 | | | 512.18 | * |
| A0A7P0TAK7 | DNA damage-binding protein 1 | | | 788.10 | * |
| Q16531 | DNA damage-binding protein 1 | | | 793.70 | * |
| A0A7P0Z4B9 | DNA damage-binding protein 1 | | | 788.10 | * |
| A0A7P0T965 | DNA damage-binding protein 1 | | | 788.10 | * |
| A0A7P0T870 | DNA damage-binding protein 1 | | | 523.60 | * |
| F5GY55 | DNA damage-binding protein 1 | | | 788.10 | * |
| F5GWI0 | DNA damage-binding protein 1 | | | 470.32 | * |
| P27695 | DNA-(apurinic or apyrimidinic site) endonuclease | | | 2759.22 | * |
| G3V5Q1 | DNA-(apurinic or apyrimidinic site) endonuclease (Fragment) | | | 2759.22 | * |
| G3V5M0 | DNA-(apurinic or apyrimidinic site) endonuclease (Fragment) | | | 2491.73 | * |
| G3V5D9 | DNA-(apurinic or apyrimidinic site) endonuclease (Fragment) | | | 2759.22 | * |
| G3V3Y6 | DNA-(apurinic or apyrimidinic site) endonuclease (Fragment) | | | 1729.16 | * |
| G3V3M6 | DNA-(apurinic or apyrimidinic site) endonuclease (Fragment) | | | 2759.22 | * |
| G3V3C7 | DNA-(apurinic or apyrimidinic site) endonuclease (Fragment) | | | 2759.22 | * |
| G3V359 | DNA-(apurinic or apyrimidinic site) endonuclease (Fragment) | | | 2491.73 | * |
| A0A0C4DGK8 | DNA-(apurinic or apyrimidinic site) endonuclease (Fragment) | | | 2759.22 | * |
| H7C4A8 | DNA-(apurinic or apyrimidinic site) endonuclease (Fragment) | | | 762.57 | * |
| Q96AE4 | Far upstream element-binding protein 1 | | | 476.72 | * |
| E9PEB5 | Far upstream element-binding protein 1 | | | 476.72 | * |
| O60812 | Heterogeneous nuclear ribonucleoprotein C-like 1 | | | 2334.81 | * |
| P26583 | High mobility group protein B2 | | | 584.65 | * |
| P16403 | Histone H1.2 | | | 707.39 | * |
| P16402 | Histone H1.3 | | | 707.39 | * |
| P10412 | Histone H1.4 | | | 707.39 | * |
| Q7L7L0 | Histone H2A type 3 | | | 8902.71 | * |
| Q5TEC6 | Histone HIST2H3PS2 | | | 3853.37 | * |
| A0A590UJL8 | Histone HIST2H3PS2 (Fragment) | | | 308.97 | * |
| B7Z9C2 | Nucleosome assembly protein 1-like 1 | | | 12126.71 | * |
| F8VV59 | Nucleosome assembly protein 1-like 1 | | | 12569.11 | * |
| F8W543 | Nucleosome assembly protein 1-like 1 | | | 6364.52 | * |
| F8VRJ2 | Nucleosome assembly protein 1-like 1 (Fragment) | | | 7046.12 | * |
| F8W118 | Nucleosome assembly protein 1-like 1 (Fragment) | | | 12569.11 | * |
| F8W020 | Nucleosome assembly protein 1-like 1 (Fragment) | | | 13048.41 | 0.006 |
| H0YHC3 | Nucleosome assembly protein 1-like 1 (Fragment) | | | 12126.71 | * |
| H0YH88 | Nucleosome assembly protein 1-like 1 (Fragment) | | | 6364.52 | * |
| Q86W56 | Poly(ADP-ribose) glycohydrolase | | | 535.17 | * |
| P12004 | Proliferating cell nuclear antigen | | | 1822.08 | * |
| B2RPK0 | Putative high mobility group protein B1-like 1 | | | 686.66 | * |
| Q6DN03 | Putative histone H2B type 2-C | | | 1916.46 | * |
| Q6DRA6 | Putative histone H2B type 2-D | | | 1916.46 | * |
| Q96T23 | Remodeling and spacing factor 1 | | | 819.16 | 0.346 |
| H0YER1 | Remodeling and spacing factor 1 (Fragment) | | | 763.29 | 0.371 |
| H7C306 | Remodeling and spacing factor 1 (Fragment) | | | 686.40 | * |
| B3KRP1 | Serine/threonine-protein kinase tousled-like 1 | | | 256.77 | * |
| Q9UKI8 | Serine/threonine-protein kinase tousled-like 1 | | | 304.32 | * |
| Q04837 | Single-stranded DNA-binding protein_ mitochondrial | | | 2874.49 | * |
| C9K0U8 | Single-stranded DNA-binding protein_ mitochondrial (Fragment) | | | 2874.49 | * |
| E7EUY5 | Single-stranded DNA-binding protein_ mitochondrial (Fragment) | | | 2874.49 | * |
| A0A0G2JLD8 | Single-stranded DNA-binding protein_ mitochondrial (Fragment) | | | 2874.49 | * |
| P12956 | X-ray repair cross-complementing protein 6 | | | 466.87 | * |
|  | **Cell Cycle** | | |  |  |
| Q0VDD7 | Break repair meiotic recombinase recruitment factor 1 | | | 714.30 | * |
| K7EIN8 | Break repair meiotic recombinase recruitment factor 1 (Fragment) | | | 495.12 | * |
| E5RIH6 | Centrosomal protein of 170 kDa (Fragment) | | | 2617.76 | * |
| A0A7I2YQH9 | Centrosome and spindle pole-associated protein 1 | | | 740.16 | * |
| Q1MSJ5 | Centrosome and spindle pole-associated protein 1 | | | 789.80 | * |
| A0A6Q8PHN8 | Centrosome and spindle pole-associated protein 1 | | | 789.80 | * |
| A0A6Q8PGS3 | Centrosome and spindle pole-associated protein 1 | | | 678.36 | * |
| A0A7I2PHE7 | Centrosome and spindle pole-associated protein 1 | | | 789.80 | * |
| A0A6Q8PF96 | Centrosome and spindle pole-associated protein 1 | | | 678.36 | * |
| A0A6Q8PF61 | Centrosome and spindle pole-associated protein 1 | | | 789.80 | * |
| A0A7I2V5Y8 | Centrosome and spindle pole-associated protein 1 | | | 638.98 | * |
| A0A7I2V5W3 | Centrosome and spindle pole-associated protein 1 | | | 789.80 | * |
| A0A7I2V5N5 | Centrosome and spindle pole-associated protein 1 | | | 740.16 | * |
| A0A7I2V5L8 | Centrosome and spindle pole-associated protein 1 | | | 628.71 | * |
| A0A7I2V5J9 | Centrosome and spindle pole-associated protein 1 | | | 628.71 | * |
| A0A7I2V5J7 | Centrosome and spindle pole-associated protein 1 | | | 638.98 | * |
| A0A7I2V5G7 | Centrosome and spindle pole-associated protein 1 | | | 628.71 | * |
| A0A7I2V5A7 | Centrosome and spindle pole-associated protein 1 | | | 661.59 | * |
| A0A7I2V4R5 | Centrosome and spindle pole-associated protein 1 | | | 789.80 | * |
| A0A7I2V4L2 | Centrosome and spindle pole-associated protein 1 | | | 678.36 | * |
| A0A7I2V3Z9 | Centrosome and spindle pole-associated protein 1 | | | 628.71 | * |
| A0A7I2V3V5 | Centrosome and spindle pole-associated protein 1 | | | 684.22 | * |
| A0A7I2V425 | Centrosome and spindle pole-associated protein 1 | | | 601.43 | * |
| A0A7I2V3Q0 | Centrosome and spindle pole-associated protein 1 | | | 649.84 | * |
| A0A7I2V3M9 | Centrosome and spindle pole-associated protein 1 | | | 678.36 | * |
| A0A7I2V3F6 | Centrosome and spindle pole-associated protein 1 | | | 589.33 | * |
| A0A7I2V3F0 | Centrosome and spindle pole-associated protein 1 | | | 740.16 | * |
| A0A7I2V398 | Centrosome and spindle pole-associated protein 1 | | | 678.36 | * |
| A0A7I2V372 | Centrosome and spindle pole-associated protein 1 | | | 678.36 | * |
| A0A7I2V2I3 | Centrosome and spindle pole-associated protein 1 | | | 611.94 | * |
| F2Z2M5 | Centrosome and spindle pole-associated protein 1 | | | 750.42 | * |
| A0A7I2V571 | Centrosome and spindle pole-associated protein 1 (Fragment) | | | 225.61 | * |
| A0A7I2V4C3 | Centrosome and spindle pole-associated protein 1 (Fragment) | | | 213.52 | * |
| A0A7I2V450 | Centrosome and spindle pole-associated protein 1 (Fragment) | | | 611.94 | * |
| A0A7I2V3G6 | Centrosome and spindle pole-associated protein 1 (Fragment) | | | 225.61 | * |
| A0A7I2V2P7 | Centrosome and spindle pole-associated protein 1 (Fragment) | | | 236.13 | * |
| A0A7I2YQX1 | Centrosome and spindle pole-associated protein 1 (Fragment) | | | 236.13 | * |
| H0YGG8 | Citron Rho-interacting kinase (Fragment) | | | 1006.92 | * |
| O14976 | Cyclin-G-associated kinase | | | 1051.12 | * |
| A8K8J9 | Dynactin 2 (P50)_ isoform CRA_b | | | 774.22 | * |
| F8W1I6 | Dynactin subunit 2 | | | 1564.54 | * |
| A0A7I2V4Y9 | Dynactin subunit 2 | | | 1062.45 | * |
| A0A7I2V4H4 | Dynactin subunit 2 | | | 1612.79 | * |
| A0A7I2V390 | Dynactin subunit 2 | | | 1172.11 | * |
| Q13561 | Dynactin subunit 2 | | | 1612.79 | * |
| H0YI98 | Dynactin subunit 2 (Fragment) | | | 381.79 | * |
| H0YHL1 | Dynactin subunit 2 (Fragment) | | | 680.67 | * |
| F8VW18 | Dynactin subunit 2 (Fragment) | | | 1172.11 | * |
| H0YAG6 | Intraflagellar transport protein 122 homolog (Fragment) | | | 896.37 | * |
| Q9UK76 | Jupiter microtubule associated homolog 1 | | | 2055.54 | * |
| Q5H907 | Melanoma antigen family D_ 2_ isoform CRA_d | | | 544.41 | * |
| Q9UNF1 | Melanoma-associated antigen D2 | | | 566.39 | * |
| Q5H909 | Melanoma-associated antigen D2 | | | 563.41 | * |
| P49321 | Nuclear autoantigenic sperm protein | | | 1835.18 | * |
| E9PPR5 | Nuclear autoantigenic sperm protein (Fragment) | | | 1067.82 | * |
| E9PNB5 | Nuclear autoantigenic sperm protein (Fragment) | | | 914.64 | * |
| Q8IVD9 | NudC domain-containing protein 3 | | | 219.12 | * |
| P31949 | Protein S100-A11 | | | 5876.83 | * |
| A8K8P3 | Protein SFI1 homolog | | | 319.72 | * |
| E5RJU6 | Protein SFI1 homolog | | | 390.22 | * |
| H7BZF3 | Protein SFI1 homolog (Fragment) | | | 317.07 | * |
| Q9H4X1 | Regulator of cell cycle RGCC | | | 1409.05 | * |
| Q5TZA2 | Rootletin | | | 331.84 | * |
| B1AKD8 | Rootletin (Fragment) | | | 296.88 | * |
| P67775 | Serine/threonine-protein phosphatase 2A catalytic subunit alpha isoform | | | 3874.14 | * |
| O00743 | Serine/threonine-protein phosphatase 6 catalytic subunit | | | 206.63 | * |
|  | TRANSCRIPTION | | |  |  |
|  | **RNA synthesis** | | |  |  |
| P53999 | Activated RNA polymerase II transcriptional coactivator p15 | | | 2191.92 | * |
| Q6UB99 | Ankyrin repeat domain-containing protein 11 | | | 753.53 | * |
| A0A087WTN8 | Ankyrin repeat domain-containing protein 11 | | | 677.74 | * |
| A0A2R8Y438 | Ankyrin repeat domain-containing protein 11 (Fragment) | | | 536.15 | * |
| Q6UB98 | Ankyrin repeat domain-containing protein 12 | | | 613.19 | 0.201 |
| B5MEA4 | Ankyrin repeat domain-containing protein 12 (Fragment) | | | 504.51 | 0.154 |
| F5GYX2 | Ankyrin repeat domain-containing protein 12 (Fragment) | | | 543.55 | 0.163 |
| A5YKK6 | CCR4-NOT transcription complex subunit 1 | | | 1366.58 | 0.199 |
| H3BMZ2 | CCR4-NOT transcription complex subunit 1 (Fragment) | | | 1242.84 | * |
| J3KS23 | Core-binding factor subunit beta | | | 2947.62 | * |
| Q13951 | Core-binding factor subunit beta | | | 3016.25 | * |
| A0A494C0A9 | Core-binding factor subunit beta | | | 3016.25 | * |
| Q96I24 | Far upstream element-binding protein 3 | | | 1466.75 | * |
| A0A0D9SFF0 | Forkhead box protein M1 | | | 1009.05 | * |
| Q08050 | Forkhead box protein M1 | | | 1009.05 | * |
| E7EVW7 | Hematopoietic lineage cell-specific protein | | | 2338.72 | * |
| P14317 | Hematopoietic lineage cell-specific protein | | | 2363.28 | * |
| A0A7I2V4G0 | Heterogeneous nuclear ribonucleoprotein A3 | | | 4774.56 | 0.650 |
| O14979 | Heterogeneous nuclear ribonucleoprotein D-like | | | 6526.85 | * |
| A0A087WUK2 | Heterogeneous nuclear ribonucleoprotein D-like | | | 6526.85 | * |
| O14529 | Homeobox protein cut-like 2 | | | 607.94 | * |
| F5GWR6 | Homeobox protein cut-like 2 (Fragment) | | | 523.75 | * |
| P31275 | Homeobox protein Hox-C12 | | | 598.53 | * |
| A6NJ46 | Homeobox protein Nkx-6.3 | | | 506.09 | * |
| P23497 | Nuclear autoantigen Sp-100 | | | 768.29 | * |
| Q9Y618 | Nuclear receptor corepressor 2 | | | 282.72 | * |
| A0A384DVL6 | Nuclear receptor corepressor 2 | | | 254.93 | * |
| C9J0Q5 | Nuclear receptor corepressor 2 | | | 267.14 | * |
| C9JE98 | Nuclear receptor corepressor 2 | | | 261.92 | * |
| C9J7T7 | Nuclear receptor corepressor 2 | | | 254.93 | * |
| C9JQE8 | Nuclear receptor corepressor 2 (Fragment) | | | 242.88 | * |
| C9JLB1 | Nucleolin | | | 2040.65 | * |
| P19338 | Nucleolin | | | 2040.65 | * |
| H7BY16 | Nucleolin | | | 2040.65 | * |
| A0A7I2V699 | Nucleolin | | | 1931.73 | * |
| A0A7I2V5M5 | Nucleolin | | | 2033.98 | * |
| A0A7I2V506 | Nucleolin | | | 1848.52 | * |
| A0A7I2V428 | Nucleolin | | | 2040.65 | * |
| A0A7I2V3F3 | Nucleolin | | | 2040.65 | * |
| A0A7I2V2U7 | Nucleolin | | | 1903.32 | * |
| A0A7I2V349 | Nucleolin | | | 1848.52 | * |
| A0A7I2V2S8 | Nucleolin | | | 1533.59 | * |
| Q99453 | Paired mesoderm homeobox protein 2B | | | 561.83 | * |
| K7ELG8 | Putative Polycomb group protein ASXL3 | | | 1879.09 | * |
| Q08117 | TLE family member 5 | | | 488.40 | * |
| K7ELE3 | TLE family member 5 (Fragment) | | | 488.40 | * |
| P20290 | Transcription factor BTF3 | | | 1350.75 | * |
| A0A7I2V5Y3 | Transcription factor BTF3 | | | 1350.75 | * |
| C9J5W6 | Transgelin | | | 632.47 | * |
| Q9UI15 | Transgelin-3 | | | 632.47 | * |
| A0A087X0H8 | TSC22 domain family protein 1 | | | 983.13 | * |
| Q15714 | TSC22 domain family protein 1 | | | 983.83 | * |
| O75157 | TSC22 domain family protein 2 | | | 1403.87 | * |
| H0Y865 | TSC22 domain family protein 2 (Fragment) | | | 987.67 | * |
| I3L0I7 | Zinc finger protein 140 (Fragment) | | | 569.14 | * |
| A0A0A0MR11 | Zinc finger protein 33A | | | 394.49 | * |
| Q06730 | Zinc finger protein 33A | | | 394.49 | * |
| G3V4H7 | Zinc finger protein 410 | | | 176.50 | * |
| J3KNQ6 | Zinc finger protein 410 | | | 176.50 | * |
| A0A0A0MTT2 | Zinc finger protein 559 | | | 735.49 | * |
| S4R3U0 | Zinc finger protein 559 (Fragment) | | | 735.49 | * |
| S4R3F2 | Zinc finger protein 559 (Fragment) | | | 632.53 | * |
| Q5VV50 | Zinc finger protein 691 | | | 961.36 | * |
| Q5VV51 | Zinc finger protein 691 (Fragment) | | | 781.67 | * |
| A8MTY0 | Zinc finger protein 724 | | | 243.39 | * |
| A0A087WUU8 | Zinc finger protein 724 (Fragment) | | | 243.39 | * |
| M0R3J2 | Zinc finger protein 724 (Fragment) | | | 243.39 | * |
| P17029 | Zinc finger protein with KRAB and SCAN domains 1 | | | 878.64 | * |
| C9JRM9 | Zinc finger protein with KRAB and SCAN domains 1 (Fragment) | | | 865.16 | * |
| E7ET52 | Zinc finger protein ZFPM2 | | | 4580.40 | * |
| E5RJX0 | Zinc finger protein ZFPM2 (Fragment) | | | 4502.44 | * |
| Q96EF9 | Zinc fingers and homeoboxes protein 1_ isoform 2 | | | 547.01 | * |
| A0A0X1KG80 | Zinc fingers and homeoboxes protein 1_ isoform 2 (Fragment) | | | 508.64 | * |
|  | **RNA processing** | | |  |  |
| Q15029 | 116 kDa U5 small nuclear ribonucleoprotein component | | | 1328.13 | * |
| K7EP67 | 116 kDa U5 small nuclear ribonucleoprotein component (Fragment) | | | 1247.04 | * |
| K7EJ74 | 116 kDa U5 small nuclear ribonucleoprotein component (Fragment) | | | 1227.27 | * |
| Q9P2I0 | Cleavage and polyadenylation specificity factor subunit 2 | | | 421.05 | * |
| H0YJF4 | Cleavage and polyadenylation specificity factor subunit 2 (Fragment) | | | 351.65 | * |
| A0A024R214 | Cytoplasmic polyadenylation element binding protein 1_ isoform CRA_c | | | 244.31 | * |
| A0A087X171 | Cytoplasmic polyadenylation element-binding protein 1 | | | 248.08 | * |
| Q9BZB8 | Cytoplasmic polyadenylation element-binding protein 1 | | | 248.08 | * |
| A0A087WXG7 | Cytoplasmic polyadenylation element-binding protein 1 | | | 244.31 | * |
| A0A087WVR7 | Cytoplasmic polyadenylation element-binding protein 1 | | | 234.58 | * |
| A0A0J9YXP0 | Cytoplasmic polyadenylation element-binding protein 1 (Fragment) | | | 244.31 | * |
| M0QYH3 | Far upstream element-binding protein 2 (Fragment) | | | 1736.32 | * |
| M0QXW7 | Far upstream element-binding protein 2 (Fragment) | | | 1010.89 | * |
| A0A7I2V3W0 | Helix-destabilizing protein | | | 18480.21 | * |
| A0A7I2V3R8 | Helix-destabilizing protein | | | 19114.03 | * |
| A0A7I2V2L6 | Helix-destabilizing protein | | | 14426.35 | * |
| F8W6I7 | Helix-destabilizing protein | | | 19114.03 | * |
| F8VZ49 | Helix-destabilizing protein (Fragment) | | | 12237.15 | * |
| F8W646 | Helix-destabilizing protein (Fragment) | | | 2333.80 | * |
| D6RD18 | Heterogeneous nuclear ribonucleoprotein A/B | | | 7818.30 | * |
| D6RBZ0 | Heterogeneous nuclear ribonucleoprotein A/B | | | 7818.30 | * |
| Q99729 | Heterogeneous nuclear ribonucleoprotein A/B | | | 5766.73 | * |
| D6R9P3 | Heterogeneous nuclear ribonucleoprotein A/B | | | 7818.30 | * |
| A0A7I2YQ85 | Heterogeneous nuclear ribonucleoprotein A1 | | | 2233.59 | * |
| P09651 | Heterogeneous nuclear ribonucleoprotein A1 | | | 19114.03 | * |
| A0A7I2YQY2 | Heterogeneous nuclear ribonucleoprotein A1 | | | 4687.68 | * |
| A0A7I2YQX9 | Heterogeneous nuclear ribonucleoprotein A1 | | | 16780.22 | * |
| H0YH80 | Heterogeneous nuclear ribonucleoprotein A1 (Fragment) | | | 11869.20 | * |
| A0A7I2V5U9 | Heterogeneous nuclear ribonucleoprotein A1 (Fragment) | | | 10332.49 | * |
| A0A7I2V666 | Heterogeneous nuclear ribonucleoprotein A1 (Fragment) | | | 4053.87 | * |
| A0A7I2V661 | Heterogeneous nuclear ribonucleoprotein A1 (Fragment) | | | 4053.87 | * |
| A0A7I2V5T1 | Heterogeneous nuclear ribonucleoprotein A1 (Fragment) | | | 1760.06 | * |
| A0A7I2V5P1 | Heterogeneous nuclear ribonucleoprotein A1 (Fragment) | | | 4053.87 | * |
| A0A7I2V5N7 | Heterogeneous nuclear ribonucleoprotein A1 (Fragment) | | | 4053.87 | * |
| A0A7I2V5H0 | Heterogeneous nuclear ribonucleoprotein A1 (Fragment) | | | 1760.06 | * |
| A0A7I2V520 | Heterogeneous nuclear ribonucleoprotein A1 (Fragment) | | | 4053.87 | * |
| A0A7I2V4P7 | Heterogeneous nuclear ribonucleoprotein A1 (Fragment) | | | 4053.87 | * |
| A0A7I2V4F8 | Heterogeneous nuclear ribonucleoprotein A1 (Fragment) | | | 4053.87 | * |
| A0A7I2V4E2 | Heterogeneous nuclear ribonucleoprotein A1 (Fragment) | | | 4053.87 | * |
| A0A7I2V497 | Heterogeneous nuclear ribonucleoprotein A1 (Fragment) | | | 3863.20 | * |
| A0A7I2V3U8 | Heterogeneous nuclear ribonucleoprotein A1 (Fragment) | | | 4053.87 | * |
| A0A7I2V459 | Heterogeneous nuclear ribonucleoprotein A1 (Fragment) | | | 4053.87 | * |
| A0A7I2V453 | Heterogeneous nuclear ribonucleoprotein A1 (Fragment) | | | 4053.87 | * |
| A0A7I2V3K5 | Heterogeneous nuclear ribonucleoprotein A1 (Fragment) | | | 4053.87 | * |
| A0A7I2V2X1 | Heterogeneous nuclear ribonucleoprotein A1 (Fragment) | | | 4053.87 | * |
| A0A7I2V360 | Heterogeneous nuclear ribonucleoprotein A1 (Fragment) | | | 4053.87 | * |
| A0A7I2V2R4 | Heterogeneous nuclear ribonucleoprotein A1 (Fragment) | | | 4053.87 | * |
| A0A7I2V334 | Heterogeneous nuclear ribonucleoprotein A1 (Fragment) | | | 5813.92 | * |
| A0A7I2V2Q7 | Heterogeneous nuclear ribonucleoprotein A1 (Fragment) | | | 4053.87 | * |
| A0A7I2V2M7 | Heterogeneous nuclear ribonucleoprotein A1 (Fragment) | | | 4053.87 | * |
| A0A7I2V2F1 | Heterogeneous nuclear ribonucleoprotein A1 (Fragment) | | | 4053.87 | * |
| F8VTQ5 | Heterogeneous nuclear ribonucleoprotein A1 (Fragment) | | | 14326.14 | * |
| A0A2R8Y4L2 | Heterogeneous nuclear ribonucleoprotein A1 pseudogene 48 | | | 12726.36 | * |
| Q32P51 | Heterogeneous nuclear ribonucleoprotein A1-like 2 | | | 10745.17 | * |
| A0A024RA28 | Heterogeneous nuclear ribonucleoprotein A2/B1_ isoform CRA_d | | | 10708.17 | * |
| A0A7I2V5D8 | Heterogeneous nuclear ribonucleoprotein A3 | | | 4680.31 | 0.650 |
| A0A7I2V402 | Heterogeneous nuclear ribonucleoprotein A3 | | | 4870.98 | 0.644 |
| H7C1J8 | Heterogeneous nuclear ribonucleoprotein A3 (Fragment) | | | 1838.55 | * |
| Q14103 | Heterogeneous nuclear ribonucleoprotein D0 | | | 17502.25 | * |
| H0Y8G5 | Heterogeneous nuclear ribonucleoprotein D0 (Fragment) | | | 17533.58 | * |
| D6RF44 | Heterogeneous nuclear ribonucleoprotein D0 (Fragment) | | | 14595.07 | * |
| D6RD83 | Heterogeneous nuclear ribonucleoprotein D0 (Fragment) | | | 5895.65 | * |
| D6RBQ9 | Heterogeneous nuclear ribonucleoprotein D0 (Fragment) | | | 6222.55 | * |
| D6RAF8 | Heterogeneous nuclear ribonucleoprotein D0 (Fragment) | | | 16947.77 | * |
| H0YA96 | Heterogeneous nuclear ribonucleoprotein D0 (Fragment) | | | 17465.69 | * |
| P52597 | Heterogeneous nuclear ribonucleoprotein F | | | 2620.66 | * |
| A0A1B0GW42 | Heterogeneous nuclear ribonucleoprotein F (Fragment) | | | 1759.78 | * |
| E9PCY7 | Heterogeneous nuclear ribonucleoprotein H | | | 7171.31 | * |
| D6RIH9 | Heterogeneous nuclear ribonucleoprotein H | | | 3100.12 | * |
| G8JLB6 | Heterogeneous nuclear ribonucleoprotein H | | | 7171.31 | * |
| D6R9D3 | Heterogeneous nuclear ribonucleoprotein H | | | 614.05 | * |
| P31943 | Heterogeneous nuclear ribonucleoprotein H | | | 7171.31 | * |
| E5RGV0 | Heterogeneous nuclear ribonucleoprotein H (Fragment) | | | 4209.80 | * |
| E5RGH4 | Heterogeneous nuclear ribonucleoprotein H (Fragment) | | | 4266.48 | * |
| E7EQJ0 | Heterogeneous nuclear ribonucleoprotein H (Fragment) | | | 2368.26 | * |
| D6RIU0 | Heterogeneous nuclear ribonucleoprotein H (Fragment) | | | 5885.35 | * |
| D6RIT2 | Heterogeneous nuclear ribonucleoprotein H (Fragment) | | | 3100.12 | * |
| D6RJ04 | Heterogeneous nuclear ribonucleoprotein H (Fragment) | | | 3100.12 | * |
| E7EN40 | Heterogeneous nuclear ribonucleoprotein H (Fragment) | | | 4431.68 | * |
| D6RFM3 | Heterogeneous nuclear ribonucleoprotein H (Fragment) | | | 3100.12 | * |
| D6RF17 | Heterogeneous nuclear ribonucleoprotein H (Fragment) | | | 1618.88 | * |
| D6RDU3 | Heterogeneous nuclear ribonucleoprotein H (Fragment) | | | 3100.12 | * |
| D6RDL0 | Heterogeneous nuclear ribonucleoprotein H (Fragment) | | | 1618.88 | * |
| D6RBM0 | Heterogeneous nuclear ribonucleoprotein H (Fragment) | | | 5912.93 | * |
| D6RAM1 | Heterogeneous nuclear ribonucleoprotein H (Fragment) | | | 3100.12 | * |
| H0YBG7 | Heterogeneous nuclear ribonucleoprotein H (Fragment) | | | 1258.38 | * |
| H0YBD7 | Heterogeneous nuclear ribonucleoprotein H (Fragment) | | | 1258.38 | * |
| H0YB39 | Heterogeneous nuclear ribonucleoprotein H (Fragment) | | | 4071.19 | * |
| H0YAQ2 | Heterogeneous nuclear ribonucleoprotein H (Fragment) | | | 1539.15 | * |
| E5RJ94 | Heterogeneous nuclear ribonucleoprotein H (Fragment) | | | 381.49 | * |
| D6R9T0 | Heterogeneous nuclear ribonucleoprotein H (Fragment) | | | 3100.12 | * |
| P55795 | Heterogeneous nuclear ribonucleoprotein H2 | | | 5801.78 | * |
| P31942 | Heterogeneous nuclear ribonucleoprotein H3 | | | 1086.41 | * |
| P61978 | Heterogeneous nuclear ribonucleoprotein K | | | 874.16 | * |
| Q5T6W2 | Heterogeneous nuclear ribonucleoprotein K (Fragment) | | | 396.35 | * |
| O60506 | Heterogeneous nuclear ribonucleoprotein Q | | | 6209.24 | * |
| A0A7I2V5Q6 | Heterogeneous nuclear ribonucleoprotein Q | | | 4892.21 | * |
| A0A7I2V4Z0 | Heterogeneous nuclear ribonucleoprotein Q | | | 6079.06 | * |
| A0A7I2V4J0 | Heterogeneous nuclear ribonucleoprotein Q | | | 2092.73 | * |
| B7Z645 | Heterogeneous nuclear ribonucleoprotein Q | | | 5627.16 | * |
| A0A7I2V346 | Heterogeneous nuclear ribonucleoprotein Q | | | 4438.24 | * |
| A0A7I2V309 | Heterogeneous nuclear ribonucleoprotein Q | | | 5757.34 | * |
| A0A7I2V2F2 | Heterogeneous nuclear ribonucleoprotein Q | | | 4697.06 | * |
| A0A7I2V2F0 | Heterogeneous nuclear ribonucleoprotein Q | | | 1640.82 | * |
| A0A7I2YQV8 | Heterogeneous nuclear ribonucleoprotein Q | | | 3699.96 | * |
| A0A7I2YQN2 | Heterogeneous nuclear ribonucleoprotein Q | | | 6079.06 | * |
| F6UXX1 | Heterogeneous nuclear ribonucleoprotein Q (Fragment) | | | 2381.99 | * |
| B4DT28 | Heterogeneous nuclear ribonucleoprotein R | | | 5023.27 | * |
| O43390 | Heterogeneous nuclear ribonucleoprotein R | | | 6664.09 | * |
| A0A6Q8PH35 | Heterogeneous nuclear ribonucleoprotein R | | | 5023.27 | * |
| A0A6Q8PHG0 | Heterogeneous nuclear ribonucleoprotein R (Fragment) | | | 3401.39 | * |
| A0A6Q8PH31 | Heterogeneous nuclear ribonucleoprotein R (Fragment) | | | 5023.27 | * |
| A0A6Q8PEX7 | Heterogeneous nuclear ribonucleoprotein R (Fragment) | | | 4903.52 | * |
| Q00839 | Heterogeneous nuclear ribonucleoprotein U | | | 926.43 | * |
| A0A1W2PQ74 | Heterogeneous nuclear ribonucleoprotein U | | | 549.74 | * |
| A0A1W2PPS1 | Heterogeneous nuclear ribonucleoprotein U | | | 920.42 | * |
| A0A1W2PPL4 | Heterogeneous nuclear ribonucleoprotein U | | | 555.09 | * |
| A0A1W2PPH7 | Heterogeneous nuclear ribonucleoprotein U | | | 555.09 | * |
| Q5RI18 | Heterogeneous nuclear ribonucleoprotein U | | | 555.09 | * |
| A0A1W2PP34 | Heterogeneous nuclear ribonucleoprotein U | | | 555.09 | * |
| A0A1X7SBS1 | Heterogeneous nuclear ribonucleoprotein U | | | 920.42 | * |
| A0A1W2PRZ7 | Heterogeneous nuclear ribonucleoprotein U (Fragment) | | | 549.74 | * |
| A0A1W2PQL0 | Heterogeneous nuclear ribonucleoprotein U (Fragment) | | | 555.09 | * |
| A0A1W2PQD4 | Heterogeneous nuclear ribonucleoprotein U (Fragment) | | | 449.30 | * |
| A0A1W2PPE9 | Heterogeneous nuclear ribonucleoprotein U (Fragment) | | | 1240.31 | * |
| A0A1W2PP35 | Heterogeneous nuclear ribonucleoprotein U (Fragment) | | | 849.71 | * |
| P22626 | Heterogeneous nuclear ribonucleoproteins A2/B1 | | | 12844.01 | * |
| A0A7I2V4S4 | Heterogeneous nuclear ribonucleoproteins A2/B1 | | | 11551.40 | * |
| A0A7I2V4N1 | Heterogeneous nuclear ribonucleoproteins A2/B1 | | | 12844.01 | * |
| A0A7I2V4N0 | Heterogeneous nuclear ribonucleoproteins A2/B1 | | | 11551.40 | * |
| A0A7I2V4I6 | Heterogeneous nuclear ribonucleoproteins A2/B1 | | | 12844.01 | * |
| A0A7I2V3P7 | Heterogeneous nuclear ribonucleoproteins A2/B1 | | | 11551.40 | * |
| A0A7I2V3P1 | Heterogeneous nuclear ribonucleoproteins A2/B1 | | | 12844.01 | * |
| A0A087WUI2 | Heterogeneous nuclear ribonucleoproteins A2/B1 | | | 11551.40 | * |
| A0A7I2V323 | Heterogeneous nuclear ribonucleoproteins A2/B1 | | | 12844.01 | * |
| A0A7I2YQN4 | Heterogeneous nuclear ribonucleoproteins A2/B1 | | | 11551.40 | * |
| B4DSU6 | Heterogeneous nuclear ribonucleoproteins C1/C2 | | | 7082.29 | * |
| G3V576 | Heterogeneous nuclear ribonucleoproteins C1/C2 | | | 9063.53 | * |
| G3V4C1 | Heterogeneous nuclear ribonucleoproteins C1/C2 | | | 10704.34 | * |
| G3V2Q1 | Heterogeneous nuclear ribonucleoproteins C1/C2 | | | 9120.55 | * |
| B2R5W2 | Heterogeneous nuclear ribonucleoproteins C1/C2 | | | 10659.92 | * |
| P07910 | Heterogeneous nuclear ribonucleoproteins C1/C2 | | | 9120.55 | * |
| B4DY08 | Heterogeneous nuclear ribonucleoproteins C1/C2 | | | 10659.92 | * |
| G3V5X6 | Heterogeneous nuclear ribonucleoproteins C1/C2 (Fragment) | | | 8640.26 | * |
| G3V5V7 | Heterogeneous nuclear ribonucleoproteins C1/C2 (Fragment) | | | 2019.67 | * |
| G3V4W0 | Heterogeneous nuclear ribonucleoproteins C1/C2 (Fragment) | | | 10659.92 | * |
| G3V575 | Heterogeneous nuclear ribonucleoproteins C1/C2 (Fragment) | | | 7170.01 | * |
| G3V555 | Heterogeneous nuclear ribonucleoproteins C1/C2 (Fragment) | | | 7170.01 | * |
| G3V4M8 | Heterogeneous nuclear ribonucleoproteins C1/C2 (Fragment) | | | 2978.43 | * |
| G3V3K6 | Heterogeneous nuclear ribonucleoproteins C1/C2 (Fragment) | | | 8640.26 | * |
| G3V2H6 | Heterogeneous nuclear ribonucleoproteins C1/C2 (Fragment) | | | 1659.91 | * |
| G3V2D6 | Heterogeneous nuclear ribonucleoproteins C1/C2 (Fragment) | | | 3647.88 | * |
| G3V251 | Heterogeneous nuclear ribonucleoproteins C1/C2 (Fragment) | | | 8640.26 | * |
| H3BR27 | RNA-binding motif protein_ X chromosome | | | 5965.55 | * |
| H3BNC1 | RNA-binding motif protein_ X chromosome | | | 3539.15 | * |
| A0A1B0GUK8 | RNA-binding motif protein_ X-linked-like-1 (Fragment) | | | 2724.18 | * |
| O75526 | RNA-binding motif protein_ X-linked-like-2 | | | 2025.16 | * |
| Q8N7X1 | RNA-binding motif protein_ X-linked-like-3 | | | 2040.27 | * |
| J3KTL2 | Serine/arginine-rich splicing factor 1 | | | 3153.55 | * |
| Q07955 | Serine/arginine-rich splicing factor 1 | | | 3153.55 | * |
| P84103 | Serine/arginine-rich splicing factor 3 | | | 3277.37 | * |
| Q16629 | Serine/arginine-rich splicing factor 7 | | | 5331.46 | * |
| Q9BRL6 | Serine/arginine-rich splicing factor 8 | | | 383.87 | * |
| J3QQV5 | Serine/arginine-rich-splicing factor 1 | | | 374.71 | * |
| J3KSW7 | Serine/arginine-rich-splicing factor 1 | | | 374.71 | * |
| J3KSR8 | Serine/arginine-rich-splicing factor 1 (Fragment) | | | 1147.47 | * |
| A0A087X2D0 | Serine/arginine-rich-splicing factor 3 | | | 3277.37 | * |
| C9JAB2 | Serine/arginine-rich-splicing factor 7 | | | 5331.46 | * |
| A0A0B4J1Z1 | Serine/arginine-rich-splicing factor 7 | | | 5331.46 | * |
| Q15393 | Splicing factor 3B subunit 3 | | | 804.17 | * |
| O43818 | U3 small nucleolar RNA-interacting protein 2 | | | 611.96 | * |
|  | PROTEIN SYNTHESIS | | |  |  |
|  | **Ribosome biogenesis** | | |  |  |
| P82909 | 28S ribosomal protein S36_ mitochondrial | | | 1349.11 | * |
| P08865 | 40S ribosomal protein AS | | | 19459.28 | * |
| A0A0C4DG17 | 40S ribosomal protein AS | | | 19459.28 | * |
| P25398 | 40S ribosomal protein S12 | | | 1902.52 | * |
| H3BNC9 | 40S ribosomal protein S17 | | | 5118.45 | * |
| P63220 | 40S ribosomal protein S21 | | | 2756.95 | * |
| Q9BYK1 | 40S ribosomal protein S21 | | | 2756.95 | * |
| Q8WVC2 | 40S ribosomal protein S21 | | | 2756.95 | * |
| P62854 | 40S ribosomal protein S26 | | | 1372.69 | * |
| A0A2R8Y422 | 40S ribosomal protein S27a | | | 11135.14 | * |
| P62857 | 40S ribosomal protein S28 | | | 23919.48 | * |
| A2A3R7 | 40S ribosomal protein S6 | | | 3065.53 | * |
| A2A3R5 | 40S ribosomal protein S6 | | | 3083.80 | * |
| P62753 | 40S ribosomal protein S6 | | | 3083.80 | * |
| P62241 | 40S ribosomal protein S8 | | | 2868.26 | * |
| Q5JR95 | 40S ribosomal protein S8 | | | 2564.85 | * |
| F8WD59 | 40S ribosomal protein SA (Fragment) | | | 15119.12 | * |
| C9J9K3 | 40S ribosomal protein SA (Fragment) | | | 19459.28 | * |
| P05386 | 60S acidic ribosomal protein P1 | | | 14795.57 | * |
| P05387 | 60S acidic ribosomal protein P2 | | | 23705.96 | * |
| H0YDD8 | 60S acidic ribosomal protein P2 (Fragment) | | | 23705.96 | * |
| H3BTH3 | 60S ribosomal protein L13 | | | 937.39 | * |
| P26373 | 60S ribosomal protein L13 | | | 3064.09 | * |
| J3QSB4 | 60S ribosomal protein L13 (Fragment) | | | 2150.21 | * |
| P50914 | 60S ribosomal protein L14 | | | 1319.45 | * |
| E7EPB3 | 60S ribosomal protein L14 | | | 1319.45 | * |
| P61313 | 60S ribosomal protein L15 | | | 3376.47 | * |
| G3V203 | 60S ribosomal protein L18 | | | 4051.41 | * |
| A0A075B7A0 | 60S ribosomal protein L18 | | | 2493.50 | * |
| F8VYV2 | 60S ribosomal protein L18 | | | 2493.50 | * |
| Q07020 | 60S ribosomal protein L18 | | | 4051.41 | * |
| J3QQ67 | 60S ribosomal protein L18 (Fragment) | | | 4051.41 | * |
| H0YHA7 | 60S ribosomal protein L18 (Fragment) | | | 4051.41 | * |
| F8VUA6 | 60S ribosomal protein L18 (Fragment) | | | 4051.41 | * |
| P84098 | 60S ribosomal protein L19 | | | 9445.06 | * |
| K7ERI7 | 60S ribosomal protein L22 | | | 2481.15 | * |
| P35268 | 60S ribosomal protein L22 | | | 2481.15 | * |
| K7EP65 | 60S ribosomal protein L22 (Fragment) | | | 2447.44 | * |
| K7EMH1 | 60S ribosomal protein L22 (Fragment) | | | 2447.44 | * |
| K7ELC4 | 60S ribosomal protein L22 (Fragment) | | | 2447.44 | * |
| K7EJT5 | 60S ribosomal protein L22 (Fragment) | | | 2447.44 | * |
| P61353 | 60S ribosomal protein L27 | | | 2754.63 | * |
| K7ELC7 | 60S ribosomal protein L27 (Fragment) | | | 2754.63 | * |
| E9PJD9 | 60S ribosomal protein L27a | | | 710.31 | * |
| P46776 | 60S ribosomal protein L27a | | | 710.31 | * |
| E9PLX7 | 60S ribosomal protein L27a | | | 710.31 | * |
| E9PLL6 | 60S ribosomal protein L27a | | | 702.52 | * |
| P62888 | 60S ribosomal protein L30 | | | 58965.91 | * |
| A0A0B4J213 | 60S ribosomal protein L30 | | | 54573.12 | * |
| A0A0C4DH44 | 60S ribosomal protein L30 | | | 31424.61 | * |
| E5RJH3 | 60S ribosomal protein L30 | | | 4392.80 | * |
| E5RI99 | 60S ribosomal protein L30 (Fragment) | | | 58965.91 | * |
| P42766 | 60S ribosomal protein L35 | | | 1437.73 | * |
| P36578 | 60S ribosomal protein L4 | | | 1456.06 | * |
| H3BM89 | 60S ribosomal protein L4 | | | 1379.98 | * |
| P46777 | 60S ribosomal protein L5 | | | 6229.08 | * |
| A0A2R8Y4A2 | 60S ribosomal protein L5 | | | 2122.60 | * |
| Q5T7N0 | 60S ribosomal protein L5 (Fragment) | | | 5214.33 | * |
| A0A2R8Y6J3 | 60S ribosomal protein L5 (Fragment) | | | 6229.08 | * |
| Q02878 | 60S ribosomal protein L6 | | | 8902.75 | * |
| U3KQR5 | 60S ribosomal protein L6 (Fragment) | | | 8706.78 | * |
| F8VZA3 | 60S ribosomal protein L6 (Fragment) | | | 8693.49 | * |
| F8VZ45 | 60S ribosomal protein L6 (Fragment) | | | 8706.78 | * |
| F8VWR1 | 60S ribosomal protein L6 (Fragment) | | | 8693.49 | * |
| F8VU16 | 60S ribosomal protein L6 (Fragment) | | | 8693.49 | * |
| A8MUD9 | 60S ribosomal protein L7 | | | 1059.46 | * |
| P18124 | 60S ribosomal protein L7 | | | 2313.08 | * |
| P62424 | 60S ribosomal protein L7a | | | 2301.70 | * |
| Q5T8U2 | 60S ribosomal protein L7a | | | 2301.70 | * |
| Q5T8U3 | 60S ribosomal protein L7a (Fragment) | | | 1864.91 | * |
| E7ERA2 | Ribosomal protein L15 | | | 696.78 | * |
| E7EQV9 | Ribosomal protein L15 (Fragment) | | | 2720.64 | * |
| E7ENU7 | Ribosomal protein L15 (Fragment) | | | 2706.42 | * |
| A0A2R8Y738 | Ribosomal protein L15 (Fragment) | | | 2679.69 | * |
| A0A2R8YEM3 | Ribosomal protein L15 (Fragment) | | | 2720.64 | * |
| E7EX53 | Ribosomal protein L15 (Fragment) | | | 2679.69 | * |
| J3QR09 | Ribosomal protein L19 | | | 9445.06 | * |
| J3KTE4 | Ribosomal protein L19 | | | 9445.06 | * |
| A0A7I2V638 | Ribosomal protein L19 | | | 8468.71 | * |
| A0A7I2V4U6 | Ribosomal protein L19 | | | 8468.71 | * |
| A0A7I2V512 | Ribosomal protein L19 | | | 8468.71 | * |
| A0A7I2V2L0 | Ribosomal protein L19 | | | 8468.71 | * |
| A0A7I2YQG2 | Ribosomal protein L19 | | | 9445.06 | * |
|  | **Translation** | | |  |  |
| A0A6Q8PGN5 | Alanine--tRNA ligase | | | 778.36 | * |
| A0A6Q8PGR9 | Alanine--tRNA ligase | | | 770.98 | * |
| A0A6Q8PGE8 | Alanine--tRNA ligase | | | 779.07 | * |
| A0A6Q8PGB5 | Alanine--tRNA ligase | | | 493.64 | * |
| A0A6Q8PFY2 | Alanine--tRNA ligase | | | 713.32 | * |
| A0A6Q8PFK3 | Alanine--tRNA ligase | | | 551.30 | * |
| H3BPK7 | Alanine--tRNA ligase | | | 779.07 | * |
| A0A6Q8PF33 | Alanine--tRNA ligase | | | 779.07 | * |
| A0A6Q8PHP7 | Alanine--tRNA ligase_ cytoplasmic | | | 465.64 | * |
| A0A6Q8PHP3 | Alanine--tRNA ligase_ cytoplasmic | | | 454.89 | * |
| A0A6Q8PHJ2 | Alanine--tRNA ligase_ cytoplasmic | | | 551.30 | * |
| A0A6Q8PH44 | Alanine--tRNA ligase_ cytoplasmic | | | 485.54 | * |
| A0A6Q8PF77 | Alanine--tRNA ligase_ cytoplasmic | | | 454.89 | * |
| P49588 | | Alanine--tRNA ligase_ cytoplasmic | | 779.07 | * |
| K7EJ19 | | Asparagine--tRNA ligase_ cytoplasmic | | 347.77 | * |
| K7EQ35 | Asparagine--tRNA ligase_ cytoplasmic (Fragment) | | | 1397.05 | * |
| K7EPK2 | Asparagine--tRNA ligase_ cytoplasmic (Fragment) | | | 1380.81 | * |
| K7EMQ6 | | Asparagine--tRNA ligase_ cytoplasmic (Fragment) | | 1049.28 | * |
| K7EIU7 | | Asparagine--tRNA ligase_ cytoplasmic (Fragment) | | 1397.05 | * |
| A0A3B3ITS3 | Aspartate--tRNA ligase_ mitochondrial | | | 849.54 | * |
| A0A3B3IT01 | Aspartate--tRNA ligase_ mitochondrial | | | 753.54 | * |
| A0A3B3ISK7 | Aspartate--tRNA ligase_ mitochondrial | | | 1016.12 | * |
| Q6PI48 | Aspartate--tRNA ligase_ mitochondrial | | | 1016.12 | * |
| A0A3B3IS54 | Aspartate--tRNA ligase_ mitochondrial | | | 586.97 | * |
| A0A3B3IS01 | Aspartate--tRNA ligase_ mitochondrial | | | 753.54 | * |
| A0A7I2V383 | | BCL2L2-PABPN1 readthrough | | 796.73 | * |
| F8VVL1 | | Density-regulated protein | | 682.74 | * |
| A0A6Q8PHI7 | Diadenosine tetraphosphate synthetase | | | 820.50 | * |
| A0A6Q8PGZ8 | Diadenosine tetraphosphate synthetase | | | 963.05 | * |
| A0A6Q8PGW4 | Diadenosine tetraphosphate synthetase | | | 953.71 | * |
| A0A6Q8PGI6 | Diadenosine tetraphosphate synthetase | | | 960.23 | * |
| A0A6Q8PGA8 | Diadenosine tetraphosphate synthetase | | | 960.23 | * |
| A0A6Q8PFZ6 | Diadenosine tetraphosphate synthetase | | | 171.29 | * |
| H7C443 | | Diadenosine tetraphosphate synthetase | | 357.89 | * |
| A0A2U3TZH3 | Elongation factor 1-alpha | | | 499.72 | * |
| A0A7I2V659 | | Elongation factor 1-alpha | | 526.46 | * |
| A0A7I2V5N4 | | Elongation factor 1-alpha | | 422.53 | * |
| A0A087WVQ9 | | Elongation factor 1-alpha | | 526.46 | * |
| A0A7I2V3H3 | | Elongation factor 1-alpha | | 422.53 | * |
| P68104 | Elongation factor 1-alpha 1 | | | 526.46 | * |
| Q05639 | Elongation factor 1-alpha 2 | | | 499.72 | * |
| F8WF65 | Elongation factor 1-beta | | | 2048.86 | * |
| F2Z2G2 | | Elongation factor 1-beta | | 2048.86 | * |
| C9JZW3 | | Elongation factor 1-beta (Fragment) | | 2048.86 | * |
| E9PRY8 | | Elongation factor 1-delta | | 4515.09 | * |
| E9PQZ1 | | Elongation factor 1-delta | | 2624.18 | * |
| E9PN91 | | Elongation factor 1-delta | | 3032.73 | * |
| E9PMW7 | | Elongation factor 1-delta | | 3437.51 | * |
| P29692 | | Elongation factor 1-delta | | 4458.64 | * |
| A0A087X1X7 | | Elongation factor 1-delta | | 4488.87 | * |
| E9PIZ1 | Elongation factor 1-delta (Fragment) | | | 3437.51 | * |
| E9PK06 | Elongation factor 1-delta (Fragment) | | | 2175.12 | * |
| E9PK01 | Elongation factor 1-delta (Fragment) | | | 4458.64 | * |
| E9PI39 | Elongation factor 1-delta (Fragment) | | | 4400.13 | * |
| E9PK72 | Elongation factor 1-delta (Fragment) | | | 1611.86 | * |
| A0A0J9YXU2 | | Elongation factor 1-delta (Fragment) | | 263.81 | * |
| H0YE72 | | Elongation factor 1-delta (Fragment) | | 457.86 | * |
| H0YE58 | | Elongation factor 1-delta (Fragment) | | 86.45 | * |
| H0YCK7 | | Elongation factor 1-delta (Fragment) | | 3437.51 | * |
| E9PQ49 | | Elongation factor 1-delta (Fragment) | | 4400.13 | * |
| E9PPR1 | | Elongation factor 1-delta (Fragment) | | 4000.78 | * |
| E9PL71 | | Elongation factor 1-delta (Fragment) | | 3995.34 | * |
| E9PL12 | | Elongation factor 1-delta (Fragment) | | 4000.78 | * |
| E9PKK3 | | Elongation factor 1-delta (Fragment) | | 1603.06 | * |
| H3BNU3 | | Elongation factor Tu_ mitochondrial | | 807.86 | * |
| P49411 | | Elongation factor Tu_ mitochondrial | | 3280.35 | * |
| K7EM18 | | Eukaryotic translation initiation factor 1 | | 1658.76 | * |
| P41567 | | Eukaryotic translation initiation factor 1 | | 1658.76 | * |
| O60739 | | Eukaryotic translation initiation factor 1b | | 1746.20 | * |
| Q13542 | | Eukaryotic translation initiation factor 4E-binding protein 2 | | 947.45 | * |
| Q15056 | | Eukaryotic translation initiation factor 4H | | 1584.79 | * |
| A0A7I2V4E4 | | Eukaryotic translation initiation factor 4H | | 1584.79 | * |
| A0A7I2V385 | | Eukaryotic translation initiation factor 4H | | 368.08 | * |
| P56537 | | Eukaryotic translation initiation factor 6 | | 1126.28 | * |
| B7ZBH1 | Eukaryotic translation initiation factor 6 (Fragment) | | | 1079.47 | * |
| A0A6Q8PGZ9 | Glycine--tRNA ligase | | | 345.05 | * |
| A0A6Q8PGN7 | Glycine--tRNA ligase | | | 345.05 | * |
| A0A6Q8PH49 | Glycine--tRNA ligase | | | 345.05 | * |
| A0A6Q8PFV5 | Glycine--tRNA ligase | | | 211.46 | * |
| A0A6Q8PFU7 | Glycine--tRNA ligase | | | 211.46 | * |
| A0A6Q8PF45 | Glycine--tRNA ligase | | | 345.05 | * |
| F8WCK4 | | Glycine--tRNA ligase | | 345.05 | * |
| P41250 | | Glycine--tRNA ligase | | 963.05 | * |
| G3XAH6 | Poly(A) polymerase | | | 950.80 | * |
| P51003 | Poly(A) polymerase alpha | | | 950.80 | * |
| H0YJL4 | | Poly(A) polymerase alpha (Fragment) | | 570.24 | * |
| A0A7I2YQ88 | Polyadenylate-binding protein | | | 1793.79 | * |
| A0A7I2YQE4 | Polyadenylate-binding protein | | | 792.25 | * |
| E7ERJ7 | Polyadenylate-binding protein | | | 1634.12 | * |
| E7EQV3 | Polyadenylate-binding protein | | | 1731.19 | * |
| B1ANR0 | | Polyadenylate-binding protein | | 1439.08 | * |
| A0A7I2V598 | | Polyadenylate-binding protein | | 467.54 | * |
| H0YAR2 | | Polyadenylate-binding protein | | 1793.79 | * |
| A0A7I2V4N4 | | Polyadenylate-binding protein | | 792.25 | * |
| A0A7I2V4L7 | | Polyadenylate-binding protein | | 1439.08 | * |
| A0A087WTT1 | | Polyadenylate-binding protein | | 1677.31 | * |
| H0Y5F5 | Polyadenylate-binding protein (Fragment) | | | 467.54 | * |
| A0A7I2V5W9 | | Polyadenylate-binding protein (Fragment) | | 467.54 | * |
| A0A7I2YQ90 | Polyadenylate-binding protein 1 | | | 1702.04 | * |
| P11940 | Polyadenylate-binding protein 1 | | | 1793.79 | * |
| A0A7I2V649 | | Polyadenylate-binding protein 1 | | 1793.79 | * |
| E5RH24 | Polyadenylate-binding protein 1 (Fragment) | | | 1001.53 | * |
| E5RGH3 | Polyadenylate-binding protein 1 (Fragment) | | | 1206.69 | * |
| H0YC10 | | Polyadenylate-binding protein 1 (Fragment) | | 395.48 | * |
| H0YBN4 | | Polyadenylate-binding protein 1 (Fragment) | | 1156.75 | * |
| H0YB86 | | Polyadenylate-binding protein 1 (Fragment) | | 520.56 | * |
| H0YAW6 | | Polyadenylate-binding protein 1 (Fragment) | | 201.25 | * |
| H0YB75 | | Polyadenylate-binding protein 1 (Fragment) | | 395.48 | * |
| H0YAS7 | | Polyadenylate-binding protein 1 (Fragment) | | 395.48 | * |
| H0YAS6 | | Polyadenylate-binding protein 1 (Fragment) | | 395.48 | * |
| E5RJB9 | | Polyadenylate-binding protein 1 (Fragment) | | 1161.20 | * |
| E5RHG7 | | Polyadenylate-binding protein 1 (Fragment) | | 1001.53 | * |
| G3V4T2 | | Polyadenylate-binding protein 2 | | 796.73 | * |
| B4DEH8 | | Polyadenylate-binding protein 2 | | 796.73 | * |
| H0YJH9 | | Polyadenylate-binding protein 2 (Fragment) | | 436.50 | * |
| Q9H361 | | Polyadenylate-binding protein 3 | | 340.13 | * |
| Q13310 | | Polyadenylate-binding protein 4 | | 1439.08 | * |
| B1ANR1 | | Polyadenylate-binding protein 4 (Fragment) | | 1131.21 | * |
| H0YCC8 | | Polyadenylate-binding protein 4 (Fragment) | | 305.29 | * |
| Q5JNZ5 | | Putative 40S ribosomal protein S26-like 1 | | 880.36 | * |
| Q5VTE0 | | Putative elongation factor 1-alpha-like 3 | | 526.46 | * |
| P49591 | | Serine--tRNA ligase_ cytoplasmic | | 1482.51 | * |
| Q5T5C7 | | Seryl-tRNA synthetase | | 1529.13 | * |
| A0A3B3ISA5 | Translation initiation factor eIF-2B subunit epsilon (Fragment) | | | 1867.55 | * |
| PROTEIN FATE (FOLDING, MODIFICATION, DESTINATION) | | | | | |
|  | | **Protein folding and stabilization** | |  |  |
| P61604 | | 10 kDa heat shock protein_ mitochondrial | | 32015.31 | 0.192 |
| B8ZZ54 | | 10 kDa heat shock protein_ mitochondrial | | 1661.05 | * |
| A0A7I2YQK6 | | 60 kDa chaperonin | | 1405.54 | * |
| A0A7I2YQ71 | | 60 kDa chaperonin | | 2071.42 | * |
| A0A7I2V5M1 | | 60 kDa chaperonin | | 2206.71 | * |
| A0A7I2V5K3 | | 60 kDa chaperonin | | 2071.42 | * |
| A0A7I2V599 | | 60 kDa chaperonin | | 2905.24 | * |
| A0A7I2V2X6 | | 60 kDa chaperonin | | 2882.11 | * |
| A0A7I2V369 | | 60 kDa chaperonin | | 2071.42 | * |
| P10809 | | 60 kDa heat shock protein_ mitochondrial | | 2908.74 | * |
| E7ESH4 | | 60 kDa heat shock protein_ mitochondrial (Fragment) | | 2071.42 | * |
| C9JL25 | | 60 kDa heat shock protein_ mitochondrial (Fragment) | | 575.32 | * |
| C9JL19 | | 60 kDa heat shock protein_ mitochondrial (Fragment) | | 282.54 | * |
| C9JCQ4 | | 60 kDa heat shock protein_ mitochondrial (Fragment) | | 282.54 | * |
| E7EXB4 | | 60 kDa heat shock protein_ mitochondrial (Fragment) | | 1939.41 | * |
| E7ERF2 | | CCT-alpha | | 1008.71 | * |
| A0A7P0T8R3 | | Endoplasmin | | 1563.03 | 0.612 |
| A0A7P0T885 | | Endoplasmin | | 1563.03 | 0.582 |
| Q14696 | | LRP chaperone MESD | | 1053.85 | * |
| A0A7I2YQC0 | | Nucleophosmin | | 25659.65 | * |
| A0A7I2V5S2 | | Nucleophosmin | | 25659.65 | * |
| A0A7I2V5J8 | | Nucleophosmin | | 5497.98 | * |
| P06748 | | Nucleophosmin | | 25659.65 | * |
| A0A7I2V579 | | Nucleophosmin | | 21643.61 | * |
| A0A7I2V4G8 | | Nucleophosmin | | 22906.58 | * |
| A0A7I2V3U2 | | Nucleophosmin | | 23676.41 | * |
| A0A7I2V433 | | Nucleophosmin | | 23676.41 | * |
| A0A7I2V3G5 | | Nucleophosmin | | 21643.61 | * |
| A0A7I2V2X9 | | Nucleophosmin | | 23676.41 | * |
| E5RI98 | | Nucleophosmin | | 5497.98 | * |
| E5RGW4 | | Nucleophosmin (Fragment) | | 6791.40 | * |
| R4GN99 | | Peptidyl-prolyl cis-trans isomerase | | 434.52 | * |
| H0Y548 | | Peptidyl-prolyl cis-trans isomerase (Fragment) | | 220.33 | * |
| Q9Y536 | | Peptidyl-prolyl cis-trans isomerase A-like 4A | | 1197.58 | * |
| P0DN37 | | Peptidyl-prolyl cis-trans isomerase A-like 4G | | 240.88 | * |
| A0A075B767 | | Peptidyl-prolyl cis-trans isomerase A-like 4H | | 330.06 | * |
| A0A7P0TB45 | | Peptidyl-prolyl cis-trans isomerase B | | 11289.02 | * |
| A0A7P0Z497 | | Peptidyl-prolyl cis-trans isomerase B | | 17971.45 | * |
| P23284 | | Peptidyl-prolyl cis-trans isomerase B | | 17971.45 | * |
| A0A7P0T7U3 | | Peptidyl-prolyl cis-trans isomerase B | | 9711.12 | * |
| Q9UHV9 | | Prefoldin subunit 2 | | 728.82 | * |
| P61758 | | Prefoldin subunit 3 | | 737.05 | * |
| C9JMN9 | | Protein disulfide-isomerase A4 (Fragment) | | 438.22 | * |
| Q8IZP2 | | Putative protein FAM10A4 | | 4766.44 | * |
| P50454 | | Serpin H1 | | 835.84 | * |
| E9PPV6 | | Serpin H1 | | 779.44 | * |
| E9PK86 | | Serpin H1 (Fragment) | | 769.77 | * |
| E9PJH8 | | Serpin H1 (Fragment) | | 835.84 | * |
| E9PR70 | | Serpin H1 (Fragment) | | 779.44 | * |
| E9PQ34 | | Serpin H1 (Fragment) | | 729.87 | * |
| E9PNX1 | | Serpin H1 (Fragment) | | 769.77 | * |
| E9PMI5 | | Serpin H1 (Fragment) | | 769.77 | * |
| F5GZI8 | | T-complex protein 1 subunit alpha | | 557.90 | * |
| P17987 | | T-complex protein 1 subunit alpha | | 1164.19 | * |
| F5H136 | | T-complex protein 1 subunit alpha (Fragment) | | 946.25 | * |
| F5H726 | | T-complex protein 1 subunit alpha (Fragment) | | 557.90 | * |
| F5H676 | | T-complex protein 1 subunit alpha (Fragment) | | 917.09 | * |
| A0A494C144 | | Tubulin-specific chaperone A | | 276.92 | * |
| E5RIX8 | | Tubulin-specific chaperone A | | 799.51 | * |
|  | | **Protein targeting, sorting and translocation** | |  |  |
| A0A590UKC4 | | AP-3 complex subunit beta | | 522.54 | * |
| F8VZJ2 | | Nascent polypeptide-associated complex subunit alpha | | 2657.93 | * |
| Q13765 | | Nascent polypeptide-associated complex subunit alpha | | 2657.93 | * |
| A0A7I2V2K5 | | Nascent polypeptide-associated complex subunit alpha | | 2657.93 | * |
| F8W0W4 | | Nascent polypeptide-associated complex subunit alpha (Fragment) | | 2657.93 | * |
| F8VNW4 | | Nascent polypeptide-associated complex subunit alpha (Fragment) | | 2657.93 | * |
| E9PE20 | | Signal recognition particle 9 kDa protein | | 1127.45 | * |
| Q659G3 | | Signal recognition particle 9 kDa protein | | 1127.45 | * |
| P49458 | | Signal recognition particle 9 kDa protein | | 4347.21 | * |
| Q6P2S0 | | Signal recognition particle 9 kDa protein | | 1127.45 | * |
|  | | **Protein modification** | |  |  |
| P30040 | | Endoplasmic reticulum resident protein 29 | | 1949.14 | * |
| F8VY02 | | Endoplasmic reticulum resident protein 29 | | 1584.98 | * |
| F8W1G0 | | Endoplasmic reticulum resident protein 29 (Fragment) | | 319.17 | * |
| A0A0C4DGP4 | | Glucosidase 2 subunit beta (Fragment) | | 883.48 | * |
| H7C0B8 | | Multifunctional procollagen lysine hydroxylase and glycosyltransferase LH3 (Fragment) | | 753.34 | * |
| O60568 | | Multifunctional procollagen lysine hydroxylase and glycosyltransferase LH3 | | 1671.01 | * |
| C9JU11 | | Multifunctional procollagen lysine hydroxylase and glycosyltransferase LH3 (Fragment) | | 310.01 | * |
| H7C2V1 | | Multifunctional procollagen lysine hydroxylase and glycosyltransferase LH3 (Fragment) | | 1127.06 | * |
| H7C2S8 | | Multifunctional procollagen lysine hydroxylase and glycosyltransferase LH3 (Fragment) | | 1193.73 | * |
| H7C2A8 | | Multifunctional procollagen lysine hydroxylase and glycosyltransferase LH3 (Fragment) | | 310.01 | * |
| F5H6X6 | | Neutral alpha-glucosidase AB | | 1711.07 | 0.663 |
| Q9UMZ3 | | Phosphatidylinositol phosphatase PTPRQ | | 2689.15 | * |
| Q99873 | | Protein arginine N-methyltransferase 1 | | 1400.45 | * |
| E9PKG1 | | Protein arginine N-methyltransferase 1 | | 1400.45 | * |
| E9PIX6 | | Protein arginine N-methyltransferase 1 (Fragment) | | 570.10 | * |
| H0YDE4 | | Protein arginine N-methyltransferase 1 (Fragment) | | 330.95 | * |
| E9PQ98 | | Protein arginine N-methyltransferase 1 (Fragment) | | 570.10 | * |
| E9PNR9 | | Protein arginine N-methyltransferase 1 (Fragment) | | 330.95 | * |
| G3V507 | | Protein arginine N-methyltransferase 5 | | 1995.44 | * |
| G3V2F5 | | Protein arginine N-methyltransferase 5 | | 1995.44 | * |
| G3V5L5 | | Protein arginine N-methyltransferase 5 (Fragment) | | 1005.55 | * |
| G3V580 | | Protein arginine N-methyltransferase 5 (Fragment) | | 3000.99 | * |
| G3V2X6 | | Protein arginine N-methyltransferase 5 (Fragment) | | 3000.99 | * |
| G3V2L6 | | Protein arginine N-methyltransferase 5 (Fragment) | | 1995.44 | * |
| H0YJX6 | | Protein arginine N-methyltransferase 5 (Fragment) | | 1056.86 | * |
| C9JSX3 | | Protein arginine N-methyltransferase 5 (Fragment) | | 2016.59 | * |
| A0A7P0TBA3 | | Protein disulfide-isomerase | | 2518.97 | * |
| A0A7P0Z4F8 | | Protein disulfide-isomerase | | 2518.97 | * |
| A0A7P0Z4B2 | | Protein disulfide-isomerase | | 2518.97 | * |
| A0A7P0T9E3 | | Protein disulfide-isomerase | | 2518.97 | * |
| A0A7P0T940 | | Protein disulfide-isomerase | | 2518.97 | * |
| I3L1Y5 | | Protein disulfide-isomerase | | 2518.97 | * |
| Q5TCY1 | | Tau-tubulin kinase 1 | | 714.31 | * |
|  | | **Protein/peptide degradation** | |  |  |
| P55036 | | 26S proteasome non-ATPase regulatory subunit 4 | | 863.58 | * |
| Q5VWC4 | | 26S proteasome non-ATPase regulatory subunit 4 | | 799.75 | * |
| A6PVX3 | | 26S proteasome non-ATPase regulatory subunit 4 (Fragment) | | 799.75 | * |
| P62333 | | 26S proteasome regulatory subunit 10B | | 1209.24 | * |
| A0A087X2I1 | | 26S proteasome regulatory subunit 10B | | 1209.24 | * |
| H0YJS8 | | 26S proteasome regulatory subunit 10B (Fragment) | | 1198.54 | * |
| H0YJC0 | | 26S proteasome regulatory subunit 10B (Fragment) | | 1198.54 | * |
| P17980 | | 26S proteasome regulatory subunit 6A | | 969.61 | * |
| E9PM69 | | 26S proteasome regulatory subunit 6A | | 969.61 | * |
| R4GNH3 | | 26S proteasome regulatory subunit 6A | | 969.61 | * |
| E9PN50 | | 26S proteasome regulatory subunit 6A (Fragment) | | 705.44 | * |
| E9PMD8 | | 26S proteasome regulatory subunit 6A (Fragment) | | 982.74 | * |
| E9PLG2 | | 26S proteasome regulatory subunit 6A (Fragment) | | 787.41 | * |
| E9PKD5 | | 26S proteasome regulatory subunit 6A (Fragment) | | 925.04 | * |
| M0R2S1 | | 60S ribosomal protein L40 (Fragment) | | 8192.47 | * |
| M0R1M6 | | 60S ribosomal protein L40 (Fragment) | | 8192.47 | * |
| F8WEH5 | | Acylamino-acid-releasing enzyme | | 1723.56 | * |
| P13798 | | Acylamino-acid-releasing enzyme | | 2100.95 | * |
| C9JLK2 | | Acylamino-acid-releasing enzyme (Fragment) | | 315.40 | * |
| H7C393 | | Acylamino-acid-releasing enzyme (Fragment) | | 2069.42 | * |
| H7C1U0 | | Acylamino-acid-releasing enzyme (Fragment) | | 315.40 | * |
| C9JIF9 | | Acyl-peptide hydrolase | | 2100.95 | * |
| A6NKB8 | | Aminopeptidase B | | 1226.52 | * |
| Q9H4A4 | | Aminopeptidase B | | 1244.50 | * |
| C9JMZ3 | | Aminopeptidase B (Fragment) | | 401.60 | * |
| A0A087WUS4 | | Aminopeptidase B (Fragment) | | 931.26 | * |
| A0A087WU27 | | Aminopeptidase B (Fragment) | | 931.26 | * |
| H7C2T3 | | Aminopeptidase B (Fragment) | | 222.53 | * |
| J3KS79 | | Bleomycin hydrolase | | 2634.80 | * |
| K7ENH5 | | Bleomycin hydrolase | | 2634.80 | * |
| Q13867 | | Bleomycin hydrolase | | 2892.37 | * |
| J3KSD8 | | Bleomycin hydrolase (Fragment) | | 231.78 | * |
| K7ES02 | | Bleomycin hydrolase (Fragment) | | 2807.96 | * |
| Q6ZSI9 | | Calpain-12 | | 1256.55 | * |
| M0R0X4 | | Calpain-12 | | 1215.10 | * |
| M0QZ20 | | Calpain-12 (Fragment) | | 1227.82 | * |
| A0A2U3TZY2 | | Caseinolytic peptidase B protein homolog | | 521.33 | * |
| F8W787 | | Cathepsin D | | 12305.64 | 0.423 |
| A0A7P0TBM7 | | Cathepsin Z | | 1063.67 | * |
| A0A7P0TBB5 | | Cathepsin Z | | 1063.67 | * |
| A0A7P0TAT6 | | Cathepsin Z | | 1138.65 | * |
| A0A7P0TB41 | | Cathepsin Z | | 1063.67 | * |
| A0A7P0TAD4 | | Cathepsin Z | | 1063.67 | * |
| A0A7P0TA25 | | Cathepsin Z | | 1063.67 | * |
| A0A7P0Z4R9 | | Cathepsin Z | | 1138.65 | * |
| A0A7P0Z4L7 | | Cathepsin Z | | 966.35 | * |
| A0A7P0Z469 | | Cathepsin Z | | 1110.93 | * |
| A0A7P0T9U4 | | Cathepsin Z | | 966.35 | * |
| A0A7P0T9U1 | | Cathepsin Z | | 1063.67 | * |
| A0A7P0T9G9 | | Cathepsin Z | | 1063.67 | * |
| A0A7P0T8X2 | | Cathepsin Z | | 1063.67 | * |
| A0A7P0T989 | | Cathepsin Z | | 1369.95 | * |
| A0A7P0T926 | | Cathepsin Z | | 1063.67 | * |
| A0A7P0T900 | | Cathepsin Z | | 1063.67 | * |
| A0A7P0T8I6 | | Cathepsin Z | | 1369.95 | * |
| Q9UBR2 | | Cathepsin Z | | 1369.95 | * |
| A0A7P0T954 | | Cullin-4B | | 554.54 | * |
| G5E979 | | Cysteine protease | | 592.73 | * |
| A0A0A0MT61 | | Cysteine protease (Fragment) | | 666.27 | * |
| H7BYM2 | | Cysteine protease (Fragment) | | 666.27 | * |
| Q8WYN0 | | Cysteine protease ATG4A | | 666.27 | * |
| Q9UHL4 | | Dipeptidyl peptidase 2 | | 13940.82 | * |
| R4GNE8 | | Dipeptidyl peptidase 2 (Fragment) | | 550.66 | * |
| R4GMV4 | | Dipeptidyl peptidase 2 (Fragment) | | 287.30 | * |
| R4GMU5 | | Dipeptidyl peptidase 2 (Fragment) | | 12485.41 | * |
| R4GMR2 | | Dipeptidyl peptidase 2 (Fragment) | | 736.32 | * |
| R4GN05 | | Dipeptidyl peptidase 2 (Fragment) | | 235.39 | * |
| Q9NY33 | | Dipeptidyl peptidase 3 | | 1293.66 | * |
| G3V1D3 | | Dipeptidyl peptidase 3 | | 1293.66 | * |
| G3V180 | | Dipeptidyl peptidase 3 | | 1293.66 | * |
| *E9PQ14* | | Dipeptidyl peptidase 3 (Fragment) | | 1155.63 | * |
| E9PPK9 | | Dipeptidyl peptidase 3 (Fragment) | | 970.01 | * |
| E9PNX5 | | Dipeptidyl peptidase 3 (Fragment) | | 970.01 | * |
| E9PKK8 | | Dipeptidyl peptidase 3 (Fragment) | | 970.01 | * |
| A0A7P0Z4C6 | | Transitional endoplasmic reticulum ATPase | | 14866.47 | 0.048 |
| E7ET84 | | E3 ubiquitin-protein ligase UBR5 | | 391.33 | * |
| E9PDE8 | | Heat shock 70 kDa protein 4L | | 1038.79 | * |
| O95757 | | Heat shock 70 kDa protein 4L | | 1044.54 | * |
| D6RJ96 | | Heat shock 70 kDa protein 4L (Fragment) | | 1041.67 | * |
| R4GN69 | | Heat shock protein 105 kDa | | 4765.29 | * |
| Q5TBM3 | | Heat shock protein 105 kDa (Fragment) | | 211.32 | * |
| H0Y6I4 | | Matrix metalloproteinase-23 (Fragment) | | 487.28 | * |
| H0YAE5 | | Matrix metalloproteinase-23 (Fragment) | | 545.33 | * |
| H0YA69 | | Matrix metalloproteinase-23 (Fragment) | | 487.28 | * |
| O75086 | | MIFR-2 | | 487.28 | * |
| E9PCB6 | | Neurolysin_ mitochondrial | | 601.15 | * |
| Q9BYT8 | | Neurolysin_ mitochondrial | | 609.49 | * |
| P0CG47 | | Polyubiquitin-B | | 17048.91 | * |
| B4DV12 | | Polyubiquitin-B | | 17048.91 | * |
| J3QS39 | | Polyubiquitin-B (Fragment) | | 17048.91 | * |
| J3QKN0 | | Polyubiquitin-B (Fragment) | | 17048.91 | * |
| J3QSA3 | | Polyubiquitin-B (Fragment) | | 12945.03 | * |
| Q5PY61 | | Polyubiquitin-C | | 17048.91 | * |
| Q96C32 | | Polyubiquitin-C | | 17048.91 | * |
| P0CG48 | | Polyubiquitin-C | | 17048.91 | * |
| F5H265 | | Polyubiquitin-C (Fragment) | | 17048.91 | * |
| F5GYU3 | | Polyubiquitin-C (Fragment) | | 17048.91 | * |
| F5GZ39 | | Polyubiquitin-C (Fragment) | | 12980.21 | * |
| F5GXK7 | | Polyubiquitin-C (Fragment) | | 17048.91 | * |
| F5H747 | | Polyubiquitin-C (Fragment) | | 17048.91 | * |
| F5H6Q2 | | Polyubiquitin-C (Fragment) | | 17048.91 | * |
| F5H2Z3 | | Polyubiquitin-C (Fragment) | | 17048.91 | * |
| F5H388 | | Polyubiquitin-C (Fragment) | | 17048.91 | * |
| P48147 | | Prolyl endopeptidase | | 1068.28 | * |
| A0A499FJL1 | | Prolyl endopeptidase | | 1037.54 | * |
| A0A087WYS6 | | Proteasome (Prosome_ macropain) subunit_ alpha type_ 8_ isoform CRA_b | | 5496.38 | * |
| J3QQN1 | | Proteasome endopeptidase complex (Fragment) | | 2031.88 | * |
| G3V5Z7 | | Proteasome subunit alpha type | | 1950.44 | * |
| G3V3U4 | | Proteasome subunit alpha type | | 495.38 | * |
| G3V3I1 | | Proteasome subunit alpha type | | 1775.72 | * |
| G3V295 | | Proteasome subunit alpha type | | 2168.87 | * |
| H0YLS6 | | Proteasome subunit alpha type | | 3349.64 | * |
| H0YLC2 | | Proteasome subunit alpha type | | 3349.64 | * |
| B4DEV8 | | Proteasome subunit alpha type | | 722.58 | * |
| H0YMZ1 | | Proteasome subunit alpha type (Fragment) | | 3679.69 | * |
| H0YMA1 | | Proteasome subunit alpha type (Fragment) | | 3349.64 | * |
| H0YL69 | | Proteasome subunit alpha type (Fragment) | | 3679.69 | * |
| F5GX11 | | Proteasome subunit alpha type-1 | | 463.54 | * |
| P25786 | | Proteasome subunit alpha type-1 | | 760.78 | * |
| H0YN18 | | Proteasome subunit alpha type-4 | | 3863.11 | * |
| P25789 | | Proteasome subunit alpha type-4 | | 3679.69 | * |
| P60900 | | Proteasome subunit alpha type-6 | | 1950.44 | * |
| H0YJC4 | | Proteasome subunit alpha type-6 (Fragment) | | 602.58 | * |
| O14818 | | Proteasome subunit alpha type-7 | | 7291.47 | * |
| H0Y586 | | Proteasome subunit alpha type-7 (Fragment) | | 4376.81 | * |
| Q8TAA3 | | Proteasome subunit alpha-type 8 | | 5496.38 | * |
| F5GY34 | | Proteasome subunit alpha-type 8 | | 596.14 | * |
| X5D2R7 | | Proteasome subunit beta | | 1391.02 | * |
| H0YKT8 | | Proteasome subunit beta (Fragment) | | 3349.64 | * |
| P20618 | | Proteasome subunit beta type-1 | | 1684.70 | * |
| P40306 | | Proteasome subunit beta type-10 | | 2031.88 | * |
| A0A140T998 | | Proteasome subunit beta type-8 | | 1277.88 | * |
| P28062 | | Proteasome subunit beta type-8 | | 1451.13 | * |
| Q5JNW7 | | Proteasome subunit beta type-8 | | 1338.00 | * |
| H3BU93 | | PSME3-interacting protein | | 1701.55 | * |
| Q9GZU8 | | PSME3-interacting protein | | 1701.55 | * |
| H3BUL4 | | PSME3-interacting protein (Fragment) | | 1611.13 | * |
| H3BTP8 | | PSME3-interacting protein (Fragment) | | 1611.13 | * |
| H3BTI2 | | PSME3-interacting protein (Fragment) | | 1600.05 | * |
| H3BSY6 | | PSME3-interacting protein (Fragment) | | 1611.13 | * |
| H3BQQ6 | | PSME3-interacting protein (Fragment) | | 1600.05 | * |
| H3BP64 | | PSME3-interacting protein (Fragment) | | 1611.13 | * |
| H3BMX9 | | PSME3-interacting protein (Fragment) | | 1611.13 | * |
| O43791 | | Speckle-type POZ protein | | 370.58 | * |
| Q9UBT2 | | SUMO-activating enzyme subunit 2 | | 645.98 | * |
| A0A7I2YQJ0 | | Transitional endoplasmic reticulum ATPase | | 1952.49 | * |
| A0A7P0TAQ1 | | Transitional endoplasmic reticulum ATPase | | 15125.16 | 0.050 |
| P55072 | | Transitional endoplasmic reticulum ATPase | | 15793.62 | 0.259 |
| A0A7P0T8Q5 | | Transitional endoplasmic reticulum ATPase | | 14882.40 | 0.050 |
| A0A7P0T8A3 | | Transitional endoplasmic reticulum ATPase | | 13992.80 | 0.047 |
| A0A7P0TAY0 | | Transitional endoplasmic reticulum ATPase (Fragment) | | 3580.02 | * |
| A0A7P0T9X5 | | Transitional endoplasmic reticulum ATPase (Fragment) | | 4081.20 | * |
| A0A7P0T8Q4 | | Transitional endoplasmic reticulum ATPase (Fragment) | | 4081.20 | * |
| A0A7P0T8D6 | | Transitional endoplasmic reticulum ATPase (Fragment) | | 4081.20 | * |
| P62979 | | Ubiquitin-40S ribosomal protein S27a | | 18128.47 | * |
| J3QTR3 | | Ubiquitin-40S ribosomal protein S27a (Fragment) | | 17048.91 | * |
| P62987 | | Ubiquitin-60S ribosomal protein L40 | | 17048.91 | * |
| M0R1V7 | | Ubiquitin-60S ribosomal protein L40 (Fragment) | | 13066.48 | * |
| P68036 | | Ubiquitin-conjugating enzyme E2 L3 | | 1363.71 | * |
| A0A1B0GUS4 | | Ubiquitin-conjugating enzyme E2 L5 | | 718.70 | * |
| Q9NQW7 | | Xaa-Pro aminopeptidase 1 | | 1033.83 | * |
| Q5T6H7 | | Xaa-Pro aminopeptidase 1 | | 878.66 | * |
| Q5T6H3 | | Xaa-Pro aminopeptidase 1 (Fragment) | | 729.37 | * |
| Q5T6H2 | | Xaa-Pro aminopeptidase 1 (Fragment) | | 734.58 | * |
| PROTEIN WITH BINDING FUNCTION OR COFACTOR REQUIREMENT | | | | | |
|  | | **Protein binding** | |  |  |
| A0A087X0J3 | | Adenylyl cyclase-associated protein | | 251.54 | * |
| E9PDI2 | | Adenylyl cyclase-associated protein | | 251.54 | * |
| A0A087WZ15 | | Adenylyl cyclase-associated protein | | 251.54 | * |
| B7Z385 | | Adenylyl cyclase-associated protein | | 251.54 | * |
| P40123 | | Adenylyl cyclase-associated protein 2 | | 251.54 | * |
| A0A6Q8PF30 | | Ankyrin repeat domain-containing protein 26 (Fragment) | | 536.96 | * |
| J3QRN1 | | Caskin-2 | | 531.18 | * |
| A0A7I2V5B1 | | HCG2043378_ isoform CRA_b | | 1020.45 | * |
| P13796 | | Plastin-2 | | 2130.73 | * |
| Q5TBN3 | | Plastin-2 (Fragment) | | 1327.79 | * |
| O75368 | | SH3 domain-binding glutamic acid-rich-like protein | | 3274.84 | * |
| D6RG15 | | Twinfilin-2 | | 1371.79 | * |
| A0A7I2V5D3 | | Twinfilin-2 | | 1661.05 | * |
| Q6IBS0 | | Twinfilin-2 | | 1661.05 | * |
| A0A7I2V2U8 | | Twinfilin-2 | | 1661.05 | * |
|  | | **Nucleic acid binding** | |  |  |
| B2RXH8 | | Heterogeneous nuclear ribonucleoprotein C-like 2 | | 2334.81 | * |
| B7ZW38 | | Heterogeneous nuclear ribonucleoprotein C-like 3 | | 2337.08 | * |
| A0A0G2JPF8 | | Heterogeneous nuclear ribonucleoprotein C-like 4 | | 2337.08 | * |
| P0DMR1 | | Heterogeneous nuclear ribonucleoprotein C-like 4 | | 2337.08 | * |
| H3BUP3 | | Major vault protein | | 1347.87 | * |
| H3BQE7 | | Major vault protein | | 1347.87 | * |
| H3BPZ2 | | Major vault protein | | 1347.87 | * |
| Q14764 | | Major vault protein | | 3718.04 | 0.220 |
| H3BUK7 | | Major vault protein (Fragment) | | 2867.88 | * |
| H3BRL2 | | Major vault protein (Fragment) | | 2943.75 | * |
| H3BQK6 | | Major vault protein (Fragment) | | 2887.34 | 0.286 |
| H3BP76 | | Major vault protein (Fragment) | | 1836.63 | * |
| H3BNF6 | | Major vault protein (Fragment) | | 2867.88 | * |
| H3BNF2 | | Major vault protein (Fragment) | | 1347.87 | * |
| Q99811 | | Paired mesoderm homeobox protein 2 | | 649.59 | * |
|  | | **Metal binding** | |  |  |
| Q6GPI0 | | ANKMY1 protein | | 835.90 | * |
| B5MBY4 | | Ankyrin repeat and MYND domain containing 1_ isoform CRA_a | | 811.83 | * |
| Q9P2S6 | | Ankyrin repeat and MYND domain-containing protein 1 | | 932.94 | * |
| J3KPY5 | | Ankyrin repeat and MYND domain-containing protein 1 | | 835.90 | * |
| J3KQ21 | | Ankyrin repeat and MYND domain-containing protein 1 | | 902.02 | * |
| H7BYI0 | | Ankyrin repeat and MYND domain-containing protein 1 (Fragment) | | 510.61 | * |
| E9PC71 | | Hippocalcin-like protein 1 | | 3073.87 | * |
| P37235 | | Hippocalcin-like protein 1 | | 3150.76 | * |
| C9JW46 | | Hippocalcin-like protein 1 (Fragment) | | 2539.02 | * |
| Q2L696 | | Nucb2 splice variant | | 579.74 | 0.275 |
| C9JKZ2 | | Nucleobindin-1 (Fragment) | | 879.23 | 0.453 |
| A0A2R8Y6G7 | | Nucleobindin-2 | | 633.81 | * |
| P80303 | | Nucleobindin-2 | | 1193.34 | 0.644 |
| E9PJP3 | | Nucleobindin-2 (Fragment) | | 561.96 | * |
| E9PLR0 | | Nucleobindin-2 (Fragment) | | 633.81 | * |
| E9PM22 | | Nucleobindin-2 (Fragment) | | 561.96 | * |
| E9PLE9 | | Nucleobindin-2 (Fragment) | | 633.81 | * |
| Q15293 | | Reticulocalbin-1 | | 2693.27 | * |
|  | | **Nucleotide/nucleoside/nucleobase binding** | |  |  |
| G3V562 | | Ras-related protein Rab-15 | | 1061.27 | * |
| G3V196 | | Ras-related protein Rab-15 | | 2054.03 | * |
| P59190 | | Ras-related protein Rab-15 | | 2054.03 | * |
| A0A2R8Y7G7 | | Ras-related protein Rab-15 | | 1066.94 | * |
| A0A2R8YFB8 | | Ras-related protein Rab-15 | | 2054.03 | * |
| A0A2R8YDI9 | | Ras-related protein Rab-15 | | 992.76 | * |
| J3QSF4 | | Ras-related protein Rab-15 (Fragment) | | 1066.94 | * |
| Q15771 | | Ras-related protein Rab-30 | | 1115.57 | * |
| E9PNB9 | | Ras-related protein Rab-30 | | 1115.57 | * |
| E9PJQ5 | | Ras-related protein Rab-30 (Fragment) | | 1115.57 | * |
| E9PI18 | | Ras-related protein Rab-30 (Fragment) | | 1115.57 | * |
| H0YDK7 | | Ras-related protein Rab-30 (Fragment) | | 1115.57 | * |
| E9PS06 | | Ras-related protein Rab-30 (Fragment) | | 1115.57 | * |
| E9PRF7 | | Ras-related protein Rab-30 (Fragment) | | 1115.57 | * |
| E9PMJ1 | | Ras-related protein Rab-30 (Fragment) | | 1115.57 | * |
| REGULATION OF METABOLISM AND PROTEIN FUNCTION | | | | | |
|  | | **Regulation of protein activity** | |  |  |
| P27482 | | Calmodulin-like protein 3 | | 301.16 | * |
| E7EQK6 | | Calpain inhibitor (Fragment) | | 1051.67 | * |
| A0A6Q8PFR3 | | Calpain inhibitor (Fragment) | | 2011.92 | * |
| D6RAA8 | | Calpain inhibitor (Fragment) | | 329.74 | * |
| H0YA91 | | Calpain inhibitor (Fragment) | | 986.15 | * |
| F8W7E0 | | Calpain inhibitor (Fragment) | | 469.82 | * |
| Q5T0Z6 | | cAMP-dependent protein kinase inhibitor | | 339.70 | * |
| Q9C010 | | cAMP-dependent protein kinase inhibitor beta | | 339.70 | * |
| A0A1W2PS52 | | Cystatin-B | | 10643.54 | * |
| P04080 | | Cystatin-B | | 24948.13 | * |
| A0A1W2PQG6 | | Cystatin-B (Fragment) | | 1530.31 | * |
| Q96CN4 | | EVI5-like protein | | 231.45 | * |
| P04264 | | Keratin_ type II cytoskeletal 1 | | 837.09 | * |
| K7EPB7 | | Thioredoxin-like protein 1 | | 3283.50 | * |
| K7ER96 | | Thioredoxin-like protein 1 (Fragment) | | 2589.08 | 0.029 |
| K7EML9 | | Thioredoxin-like protein 1 (Fragment) | | 940.43 | * |
| K7EME7 | | Thioredoxin-like protein 1 (Fragment) | | 239.77 | * |
| K7EKG2 | | Thioredoxin-like protein 1 (Fragment) | | 3283.50 | * |
| CELLULAR TRANSPORT, TRANSPORT FACILITIES AND TRANSPORT ROUTES | | | | | |
|  | | **Transport compounds (substrates)** | |  |  |
| Q8WWZ4 | | ATP-binding cassette sub-family A member 10 | | 432.04 | * |
| P49792 | | E3 SUMO-protein ligase RanBP2 | | 266.84 | * |
| Q9BSJ8 | | Extended synaptotagmin-1 | | 859.98 | * |
| F8VZB1 | | Extended synaptotagmin-1 (Fragment) | | 1115.81 | * |
| P15090 | | Fatty acid-binding protein_ adipocyte | | 1813.27 | * |
| Q9HAV7 | | GrpE protein homolog 1_ mitochondrial | | 1288.72 | * |
| P62826 | | GTP-binding nuclear protein Ran | | 4011.87 | * |
| B5MDF5 | | GTP-binding nuclear protein Ran | | 4011.87 | * |
| B4DV51 | | GTP-binding nuclear protein Ran | | 3356.90 | * |
| F5H018 | | GTP-binding nuclear protein Ran (Fragment) | | 4011.87 | * |
| J3KQE5 | | GTP-binding nuclear protein Ran (Fragment) | | 4011.87 | * |
| H0YFC6 | | GTP-binding nuclear protein Ran (Fragment) | | 654.97 | * |
| P61970 | | Nuclear transport factor 2 | | 2699.36 | * |
| H3BRV9 | | Nuclear transport factor 2 (Fragment) | | 687.58 | * |
| A0A7I2V5U0 | | Phospholipid-transporting ATPase ABCA1 | | 395.07 | * |
| O95477 | | Phospholipid-transporting ATPase ABCA1 | | 395.07 | * |
| Q5SX90 | | Rab GDP dissociation inhibitor (Fragment) | | 6364.58 | * |
| V9GYJ7 | | Rab GDP dissociation inhibitor (Fragment) | | 6364.58 | * |
| V9GYF8 | | Rab GDP dissociation inhibitor (Fragment) | | 6364.58 | * |
| A8MTC6 | | RAB37_ member RAS oncogene family_ isoform CRA_a | | 1277.93 | * |
| A8MSP2 | | RAB37_ member RAS oncogene family_ isoform CRA_e | | 1123.17 | * |
| O60518 | | Ran-binding protein 6 | | 515.40 | * |
| P51153 | | Ras-related protein Rab-13 | | 1039.90 | * |
| Q96AX2 | | Ras-related protein Rab-37 | | 1277.93 | * |
| A8MZI4 | | Ras-related protein Rab-37 | | 1123.17 | * |
| B7Z3L0 | | Ras-related protein Rab-37 | | 1277.93 | * |
| P20337 | | Ras-related protein Rab-3B | | 1443.48 | * |
| O95716 | | Ras-related protein Rab-3D | | 1160.38 | * |
| Q8TD43 | | Transient receptor potential cation channel subfamily M member 4 | | 511.36 | * |
|  | | **Transport facilities** | |  |  |
| Q9Y696 | | Chloride intracellular channel protein 4 | | 4303.28 | * |
| M0R1J0 | | Intermediate conductance calcium-activated potassium channel protein 4 (Fragment) | | 644.16 | * |
| H0YMP8 | | Phospholipid-transporting ATPase | | 382.71 | * |
| Q8TF62 | | Probable phospholipid-transporting ATPase IM | | 413.28 | * |
| A0A0A6YY98 | | Transient receptor potential cation channel subfamily V member 5 | | 700.04 | * |
| Q9NQA5 | | Transient receptor potential cation channel subfamily V member 5 | | 700.04 | * |
| H7C2J6 | | Transient receptor potential cation channel subfamily V member 5 (Fragment) | | 700.04 | * |
|  | | **Transport routes** | |  |  |
| H0YN26 | | Acidic leucine-rich nuclear phosphoprotein 32 family member A | | 4576.88 | * |
| P39687 | | Acidic leucine-rich nuclear phosphoprotein 32 family member A | | 4576.88 | * |
| H7BZ09 | | Acidic leucine-rich nuclear phosphoprotein 32 family member A | | 320.86 | * |
| Q92688 | | Acidic leucine-rich nuclear phosphoprotein 32 family member B | | 4256.03 | * |
| O43423 | | Acidic leucine-rich nuclear phosphoprotein 32 family member C | | 195.15 | * |
| A0A1B0GVS3 | | Alpha-centractin | | 235.30 | * |
| P61163 | | Alpha-centractin | | 235.30 | * |
| R4GMT0 | | Alpha-centractin | | 235.30 | * |
| B4E1S2 | | Annexin | | 8902.37 | * |
| Q6P452 | | Annexin | | 14528.11 | 0.023 |
| E5RK69 | | Annexin | | 5423.25 | * |
| E5RI05 | | Annexin | | 2703.50 | * |
| H0YC77 | | Annexin (Fragment) | | 1565.84 | * |
| E5RK63 | | Annexin (Fragment) | | 9264.53 | * |
| E5RJR0 | | Annexin (Fragment) | | 2517.18 | * |
| E5RJF5 | | Annexin (Fragment) | | 9264.53 | * |
| P09525 | | Annexin A4 | | 15830.17 | 0.023 |
| E5RIU8 | | Annexin A6 | | 2517.18 | * |
| E5RFF0 | | Annexin A6 (Fragment) | | 4083.02 | * |
| A0A590UKD3 | | AP complex subunit beta | | 500.02 | * |
| A0A590UJW5 | | AP complex subunit beta | | 500.02 | * |
| A0A590UJ60 | | AP-3 complex subunit beta | | 522.54 | * |
| A0A5F9UJV3 | | AP-3 complex subunit beta | | 522.54 | * |
| A0A2R8Y2A8 | | AP-3 complex subunit beta | | 522.54 | * |
| A0A590UK04 | | AP-3 complex subunit beta | | 522.54 | * |
| A0A590UJ44 | | AP-3 complex subunit beta-2 | | 399.03 | * |
| F5GYB0 | | AP-3 complex subunit beta-2 | | 399.03 | * |
| Q13367 | | AP-3 complex subunit beta-2 | | 522.54 | * |
| A0A590UJ88 | | AP-3 complex subunit beta-2 | | 399.03 | * |
| A0A590UK69 | | AP-3 complex subunit beta-2 | | 399.03 | * |
| A0A590UJS5 | | AP-3 complex subunit beta-2 | | 399.03 | * |
| F5GWU4 | | AP-3 complex subunit beta-2 (Fragment) | | 445.38 | * |
| Q9NZZ3 | | Charged multivesicular body protein 5 | | 1148.01 | * |
| H7C3C7 | | Chromosome 1 open reading frame 142_ isoform CRA_a | | 589.51 | * |
| F8WF69 | | Clathrin light chain | | 293.03 | * |
| P09496 | | Clathrin light chain A | | 482.75 | * |
| Q9UJU6 | | Drebrin-like protein | | 3988.90 | * |
| F8WFE1 | | Drebrin-like protein | | 2500.08 | * |
| F8WCK3 | | Drebrin-like protein | | 658.04 | * |
| F8WC20 | | Drebrin-like protein | | 2500.08 | * |
| F8WBG8 | | Drebrin-like protein | | 3648.24 | * |
| F8WBB2 | | Drebrin-like protein | | 2500.08 | * |
| F8WB73 | | Drebrin-like protein | | 658.04 | * |
| B4DDD6 | | Drebrin-like protein | | 4018.05 | * |
| F2Z3E3 | | Drebrin-like protein | | 658.04 | * |
| F2Z2V3 | | Drebrin-like protein | | 658.04 | * |
| H0Y5J4 | | Drebrin-like protein (Fragment) | | 340.66 | * |
| G3V3E8 | | Epididymal secretory protein E1 | | 9755.63 | * |
| E7EMS2 | | Epididymal secretory protein E1 | | 9755.63 | * |
| J3KMY5 | | Epididymal secretory protein E1 | | 9755.63 | * |
| G3V3D1 | | Epididymal secretory protein E1 (Fragment) | | 9755.63 | * |
| G3V2V8 | | Epididymal secretory protein E1 (Fragment) | | 9598.93 | * |
| H0YIZ1 | | Epididymal secretory protein E1 (Fragment) | | 8094.63 | * |
| P52907 | | F-actin-capping protein subunit alpha-1 | | 1545.25 | * |
| P47755 | | F-actin-capping protein subunit alpha-2 | | 241.80 | * |
| P47756 | | F-actin-capping protein subunit beta | | 1394.96 | * |
| B1AK88 | | F-actin-capping protein subunit beta | | 1453.31 | * |
| B1AK87 | | F-actin-capping protein subunit beta | | 1377.38 | * |
| B1AK85 | | F-actin-capping protein subunit beta | | 1096.20 | * |
| A0A6I8PRV6 | | F-actin-capping protein subunit beta (Fragment) | | 902.03 | * |
| Q6PIK3 | | HCG1995540_ isoform CRA_b | | 1222.52 | * |
| H0YHE0 | | HEAT repeat-containing protein 5A (Fragment) | | 497.64 | * |
| E7EVH7 | | Kinesin light chain | | 1293.40 | * |
| G3V5R9 | | Kinesin light chain | | 1328.68 | * |
| G3V3H3 | | Kinesin light chain | | 1328.68 | * |
| G3V2E7 | | Kinesin light chain | | 1293.40 | * |
| G5E9S8 | | Kinesin light chain | | 1293.40 | * |
| F8W6L3 | | Kinesin light chain | | 1293.40 | * |
| Q07866 | | Kinesin light chain 1 | | 1293.40 | * |
| H0YJU9 | | Kinesin light chain 1 (Fragment) | | 1168.26 | * |
| H0YJT3 | | Kinesin light chain 1 (Fragment) | | 1168.26 | * |
| H0YJL0 | | Kinesin light chain 1 (Fragment) | | 1168.26 | * |
| H0YGB8 | | Kinesin light chain 1 (Fragment) | | 1113.60 | * |
| H0YG16 | | Kinesin light chain 1 (Fragment) | | 1168.26 | * |
| P61916 | | NPC intracellular cholesterol transporter 2 | | 9755.63 | * |
| Q9HCD6 | | Protein TANC2 | | 540.53 | * |
| Q92928 | | Putative Ras-related protein Rab-1C | | 3493.52 | * |
| G5E9U5 | | Rab GDP dissociation inhibitor | | 2297.63 | * |
| Q96D71 | | RalBP1-associated Eps domain-containing protein 1 | | 407.83 | * |
| E9PMG1 | | RalBP1-associated Eps domain-containing protein 1 | | 407.83 | * |
| H0YDT0 | | RalBP1-associated Eps domain-containing protein 1 (Fragment) | | 402.47 | * |
| C9JIC6 | | Ran-specific GTPase-activating protein (Fragment) | | 315.56 | * |
| C9JGV6 | | Ran-specific GTPase-activating protein (Fragment) | | 403.30 | * |
| C9JXG8 | | Ran-specific GTPase-activating protein (Fragment) | | 525.41 | * |
| P61026 | | Ras-related protein Rab-10 | | 2701.90 | * |
| A0A3B3ITT1 | | Ras-related protein Rab-12 | | 1147.69 | * |
| Q6IQ22 | | Ras-related protein Rab-12 | | 1147.69 | * |
| P62820 | | Ras-related protein Rab-1A | | 3935.31 | * |
| E7END7 | | Ras-related protein Rab-1A | | 2677.58 | * |
| E9PLD0 | | Ras-related protein Rab-1B | | 2781.04 | * |
| Q9H0U4 | | Ras-related protein Rab-1B | | 3896.61 | * |
| Q9H082 | | Ras-related protein Rab-33B | | 980.94 | * |
| A0A494C0Z5 | | Ras-related protein Rab-33B | | 980.94 | * |
| Q15286 | | Ras-related protein Rab-35 | | 2147.31 | * |
| F5H157 | | Ras-related protein Rab-35 (Fragment) | | 2147.31 | * |
| Q14964 | | Ras-related protein Rab-39A | | 1063.46 | * |
| Q96DA2 | | Ras-related protein Rab-39B | | 1115.57 | * |
| P20336 | | Ras-related protein Rab-3A | | 1115.57 | * |
| M0R257 | | Ras-related protein Rab-3A (Fragment) | | 1115.57 | * |
| Q86YS6 | | Ras-related protein Rab-43 | | 1390.44 | * |
| C9JFM7 | | Ras-related protein Rab-43 (Fragment) | | 1204.99 | * |
| P20338 | | Ras-related protein Rab-4A | | 1450.04 | * |
| P61018 | | Ras-related protein Rab-4B | | 1336.96 | * |
| M0R0X1 | | Ras-related protein Rab-4B (Fragment) | | 1336.96 | * |
| Q9NRW1 | | Ras-related protein Rab-6B | | 1330.47 | * |
| J3KR73 | | Ras-related protein Rab-6B (Fragment) | | 1123.33 | * |
| C9JB90 | | Ras-related protein Rab-6B (Fragment) | | 1004.87 | * |
| C9JU14 | | Ras-related protein Rab-6B (Fragment) | | 1123.33 | * |
| C9J4V0 | | Ras-related protein Rab-7a | | 1194.25 | * |
| C9J4S4 | | Ras-related protein Rab-7a | | 1092.27 | * |
| P51149 | | Ras-related protein Rab-7a | | 1251.43 | * |
| A0A6Q8PH84 | | Ras-related protein Rab-7a | | 1251.43 | * |
| A0A6Q8PGE6 | | Ras-related protein Rab-7a | | 1243.75 | * |
| A0A6Q8PG52 | | Ras-related protein Rab-7a | | 1251.43 | * |
| C9J8S3 | | Ras-related protein Rab-7a | | 1194.25 | * |
| P61006 | | Ras-related protein Rab-8A | | 2108.33 | * |
| H0YMN7 | | Ras-related protein Rab-8B | | 2108.33 | * |
| Q92930 | | Ras-related protein Rab-8B | | 2213.38 | * |
| H0YLJ8 | | Ras-related protein Rab-8B | | 992.76 | * |
| H0YL94 | | Ras-related protein Rab-8B | | 992.76 | * |
| H0YNE9 | | Ras-related protein Rab-8B (Fragment) | | 2213.38 | * |
| A0A087X0B7 | | Synaptosomal-associated protein 47 | | 622.18 | * |
| A0A7P0Z4P6 | | Synaptosomal-associated protein 47 | | 589.51 | * |
| A0A7P0T9T2 | | Synaptosomal-associated protein 47 | | 589.51 | * |
| A0A7P0T8U4 | | Synaptosomal-associated protein 47 | | 589.51 | * |
| A0A7P0T899 | | Synaptosomal-associated protein 47 | | 589.51 | * |
| Q5SQN1 | | Synaptosomal-associated protein 47 | | 622.18 | * |
| A0A087X2J6 | | Synaptosomal-associated protein 47 | | 589.51 | * |
| A0A7P0TBK4 | | Synaptosomal-associated protein 47 (Fragment) | | 583.13 | * |
| U3KPT7 | | Synaptosomal-associated protein 47 (Fragment) | | 454.03 | * |
| C9J3F6 | | TBC1 domain family member 5 | | 236.12 | * |
| Q9UBQ0 | | Vacuolar protein sorting-associated protein 29 | | 2030.72 | * |
| F8VXU5 | | Vacuolar protein sorting-associated protein 29 | | 2030.72 | * |
| CELLULAR COMMUNICATION/SIGNAL TRANSDUCTION MECHANISM | | | | | |
|  | | **Cellular signalling** | |  |  |
| I3L3T1 | | 14-3-3 protein epsilon | | 10312.37 | * |
| B4DJF2 | | 14-3-3 protein epsilon | | 10312.37 | * |
| K7EIT4 | | 14-3-3 protein epsilon (Fragment) | | 11357.19 | * |
| Q04917 | | 14-3-3 protein eta | | 14460.61 | * |
| A2IDB2 | | 14-3-3 protein eta (Fragment) | | 9917.66 | * |
| P61981 | | 14-3-3 protein gamma | | 18426.56 | 0.103 |
| P31947 | | 14-3-3 protein sigma | | 14078.11 | * |
| E9PG15 | | 14-3-3 protein theta (Fragment) | | 11584.39 | * |
| Q9Y2I7 | | 1-phosphatidylinositol 3-phosphate 5-kinase | | 416.10 | * |
| C9JL08 | | 1-phosphatidylinositol 3-phosphate 5-kinase (Fragment) | | 311.48 | * |
| E9PDH4 | | 1-phosphatidylinositol 3-phosphate 5-kinase (Fragment) | | 390.03 | * |
| A0A7P0TAJ1 | | A-kinase anchor protein 9 (Fragment) | | 276.68 | * |
| H0YKL9 | | Annexin (Fragment) | | 20497.41 | * |
| H0YKN4 | | Annexin A2 | | 15857.19 | * |
| H0YMD9 | | Annexin A2 (Fragment) | | 20412.83 | * |
| H0YKV8 | | Annexin A2 (Fragment) | | 20497.41 | * |
| M0QZ52 | | Calmodulin 3 (Phosphorylase kinase_ delta)_ isoform CRA_d | | 19138.35 | 0.027 |
| G3V479 | | Calmodulin-1 | | 8218.57 | * |
| F8WBR5 | | Calmodulin-2 | | 8218.57 | * |
| P67870 | | Casein kinase II subunit beta | | 1481.05 | * |
| A0A7I2YQ78 | | Casein kinase II subunit beta | | 2004.63 | * |
| A0A0G2JM58 | | Casein kinase II subunit beta | | 1392.64 | * |
| A0A0G2JM12 | | Casein kinase II subunit beta | | 1392.64 | * |
| N0E644 | | Casein kinase II subunit beta | | 1392.64 | * |
| N0E472 | | Casein kinase II subunit beta | | 1392.64 | * |
| Q5SRQ6 | | Casein kinase II subunit beta | | 2004.63 | * |
| Q5SRQ3 | | Casein kinase II subunit beta | | 1422.28 | * |
| A0A7I2V500 | | Casein kinase II subunit beta | | 1916.23 | * |
| A0A7I2YQQ2 | | Casein kinase II subunit beta | | 1916.23 | * |
| C9K0C4 | | DCC-interacting protein 13-alpha (Fragment) | | 473.89 | * |
| E9PD68 | | Dihydropyrimidinase-related protein 1 | | 2279.99 | * |
| Q14194 | | Dihydropyrimidinase-related protein 1 | | 2279.99 | * |
| Q9UI08 | | Ena/VASP-like protein | | 1132.11 | * |
| G3V314 | | Ena/VASP-like protein (Fragment) | | 1023.72 | * |
| H0YJN0 | | Ena/VASP-like protein (Fragment) | | 1101.05 | * |
| H0YJL6 | | Ena/VASP-like protein (Fragment) | | 1023.72 | * |
| O94887 | | FERM_ ARHGEF and pleckstrin domain-containing protein 2 | | 519.93 | * |
| H7C210 | | FERM_ ARHGEF and pleckstrin domain-containing protein 2 (Fragment) | | 951.39 | * |
| Q9H706 | | GRB2-associated and regulator of MAPK protein 1 | | 475.92 | * |
| F6S2S5 | | LIM and SH3 domain protein 1 | | 2123.62 | * |
| K7ESD6 | | LIM and SH3 domain protein 1 | | 1036.27 | * |
| Q14847 | | LIM and SH3 domain protein 1 | | 16423.48 | * |
| J3KSN1 | | LIM and SH3 domain protein 1 (Fragment) | | 9274.97 | * |
| C9J9W2 | | LIM and SH3 domain protein 1 (Fragment) | | 13498.93 | * |
| E5RGA9 | | Neurocalcin-delta | | 354.26 | * |
| P61601 | | Neurocalcin-delta | | 367.10 | * |
| E5RHC8 | | Neurocalcin-delta (Fragment) | | 354.26 | * |
| E5RGZ0 | | Neurocalcin-delta (Fragment) | | 354.26 | * |
| E5RFL9 | | Neurocalcin-delta (Fragment) | | 354.26 | * |
| A0A1B0GXG3 | | Neurocalcin-delta (Fragment) | | 354.26 | * |
| E5RK89 | | Neurocalcin-delta (Fragment) | | 354.26 | * |
| E5RJT1 | | Neurocalcin-delta (Fragment) | | 354.26 | * |
| E5RJJ6 | | Neurocalcin-delta (Fragment) | | 354.26 | * |
| E5RIZ1 | | Neurocalcin-delta (Fragment) | | 354.26 | * |
| E5RIG4 | | Neurocalcin-delta (Fragment) | | 354.26 | * |
| E5RIA5 | | Neurocalcin-delta (Fragment) | | 413.84 | * |
| E5RI95 | | Neurocalcin-delta (Fragment) | | 354.26 | * |
| E5RI78 | | Neurocalcin-delta (Fragment) | | 354.26 | * |
| E5RHE8 | | Neurocalcin-delta (Fragment) | | 354.26 | * |
| P84074 | | Neuron-specific calcium-binding protein hippocalcin | | 475.35 | * |
| A0A2R8Y7X6 | | Peripheral plasma membrane protein CASK (Fragment) | | 540.02 | * |
| P30086 | | Phosphatidylethanolamine-binding protein 1 | | 12786.87 | * |
| A0A1B0GW72 | | Pleckstrin homology domain-containing family G member 4B | | 208.50 | * |
| Q96PX9 | | Pleckstrin homology domain-containing family G member 4B | | 201.29 | * |
| P08567 | | Pleckstrin | | 3057.69 | * |
| Q8IX03 | | Protein KIBRA | | 577.40 | * |
| H3BLZ3 | | Protein KIBRA (Fragment) | | 568.91 | * |
| H0YBE8 | | Protein KIBRA (Fragment) | | 551.28 | * |
| H0YAU4 | | Protein KIBRA (Fragment) | | 537.35 | * |
| Q6AWC2 | | Protein WWC2 | | 645.02 | * |
| D6R9P8 | | Protein WWC2 | | 560.51 | * |
| H0YC23 | | Protein-serine/threonine phosphatase (Fragment) | | 2387.19 | * |
| A8MUU1 | | Putative fatty acid-binding protein 5-like protein 3 | | 1685.87 | * |
| P63244 | | Receptor of activated protein C kinase 1 | | 21145.68 | * |
| D6R909 | | Receptor of-activated protein C kinase 1 | | 12731.45 | * |
| E9PD14 | | Receptor of-activated protein C kinase 1 | | 13269.93 | * |
| J3KPE3 | | Receptor of-activated protein C kinase 1 | | 10906.64 | * |
| D6RHJ5 | | Receptor of-activated protein C kinase 1 | | 2492.41 | * |
| D6RHH4 | | Receptor of-activated protein C kinase 1 | | 15808.90 | * |
| D6RGK8 | | Receptor of-activated protein C kinase 1 | | 12731.45 | * |
| D6RF23 | | Receptor of-activated protein C kinase 1 | | 3721.49 | * |
| D6RDI0 | | Receptor of-activated protein C kinase 1 | | 2492.41 | * |
| D6RAU2 | | Receptor of-activated protein C kinase 1 | | 13269.93 | * |
| D6RAC2 | | Receptor of-activated protein C kinase 1 | | 18875.79 | * |
| H0Y9P0 | | Receptor of-activated protein C kinase 1 (Fragment) | | 3721.49 | * |
| H0Y8W2 | | Receptor of-activated protein C kinase 1 (Fragment) | | 8414.23 | * |
| H0Y8R5 | | Receptor of-activated protein C kinase 1 (Fragment) | | 5417.83 | * |
| D6RFZ9 | | Receptor of-activated protein C kinase 1 (Fragment) | | 15001.35 | * |
| D6RFX4 | | Receptor of-activated protein C kinase 1 (Fragment) | | 14931.79 | * |
| D6REE5 | | Receptor of-activated protein C kinase 1 (Fragment) | | 18875.79 | * |
| D6RBD0 | | Receptor of-activated protein C kinase 1 (Fragment) | | 15270.42 | * |
| H0YAM7 | | Receptor of-activated protein C kinase 1 (Fragment) | | 8380.48 | * |
| H0YAF8 | | Receptor of-activated protein C kinase 1 (Fragment) | | 8414.23 | * |
| D6R9Z1 | | Receptor of-activated protein C kinase 1 (Fragment) | | 14931.79 | * |
| D6R9L0 | | Receptor of-activated protein C kinase 1 (Fragment) | | 14931.79 | * |
| G3V112 | | Regulator of G-protein signalling 22_ isoform CRA_c | | 608.21 | * |
| A0A087WV61 | | Regulator of G-protein-signaling 22 | | 614.12 | * |
| E5RGJ7 | | Regulator of G-protein-signaling 22 (Fragment) | | 196.77 | * |
| A0A087X0Z0 | | Rho GTPase-activating protein 23 (Fragment) | | 1695.88 | * |
| P08134 | | Rho-related GTP-binding protein RhoC | | 1543.27 | * |
| Q5JR05 | | Rho-related GTP-binding protein RhoC | | 1001.57 | * |
| E9PLA2 | | Rho-related GTP-binding protein RhoC | | 983.25 | * |
| Q5JR08 | | Rho-related GTP-binding protein RhoC (Fragment) | | 1543.27 | * |
| Q5JR07 | | Rho-related GTP-binding protein RhoC (Fragment) | | 1001.57 | * |
| Q5JR06 | | Rho-related GTP-binding protein RhoC (Fragment) | | 983.25 | * |
| E9PQH6 | | Rho-related GTP-binding protein RhoC (Fragment) | | 1001.57 | * |
| E9PN11 | | Rho-related GTP-binding protein RhoC (Fragment) | | 983.25 | * |
| E5RHC1 | | Serine/threonine-protein phosphatase (Fragment) | | 1179.64 | * |
| P62714 | | Serine/threonine-protein phosphatase 2A catalytic subunit beta isoform | | 2662.48 | * |
| H0YBN9 | | Serine/threonine-protein phosphatase 2A catalytic subunit beta isoform (Fragment) | | 262.75 | * |
| Q9NRF2 | | SH2B adapter protein 1 | | 581.91 | * |
| O14492 | | SH2B adapter protein 2 | | 696.01 | * |
| C9JK89 | | SH2B adapter protein 2 (Fragment) | | 696.01 | * |
| G3V5H7 | | SKI family transcriptional corepressor 1 | | 1102.56 | * |
| G3V3E1 | | SKI family transcriptional corepressor 1 | | 1102.56 | * |
| P84550 | | SKI family transcriptional corepressor 1 | | 1115.76 | * |
| F8WB84 | | Sonic hedgehog protein | | 1038.38 | * |
| Q6IQ16 | | Speckle-type POZ protein-like | | 478.73 | * |
| P61586 | | Transforming protein RhoA | | 1699.96 | * |
| C9JX21 | | Transforming protein RhoA | | 1154.57 | * |
| A0A7I2V5E6 | | Transforming protein RhoA | | 983.25 | * |
| A0A7I2V3G1 | | Transforming protein RhoA | | 1001.57 | * |
| C9JRM1 | | Transforming protein RhoA | | 983.25 | * |
| A0A7I2YQV1 | | Transforming protein RhoA | | 1681.64 | * |
| C9JNR4 | | Transforming protein RhoA (Fragment) | | 1001.57 | * |
|  | | CELL RESCUE, DEFENSE AND VIRULENCE | |  |  |
|  | | **Stress response** | |  |  |
| A0A7I2V2S7 | | 75 kDa glucose-regulated protein | | 7343.21 | 0.677 |
| A0A7I2V2G2 | | 75 kDa glucose-regulated protein | | 7419.77 | 0.670 |
| P04040 | | Catalase | | 6229.48 | * |
| P00390 | | Glutathione reductase_ mitochondrial | | 3504.57 | * |
| H0YC68 | | Glutathione reductase_ mitochondrial (Fragment) | | 1247.97 | * |
| A0A2R8YF59 | | Glutathione reductase_ mitochondrial (Fragment) | | 2577.27 | * |
| P78417 | | Glutathione S-transferase omega-1 | | 1469.15 | * |
| Q5TA01 | | Glutathione S-transferase omega-1 (Fragment) | | 1455.32 | * |
| A0A2R8Y5E5 | | Glutathione S-transferase P | | 1096.33 | * |
| A0A087X2E9 | | Glutathione S-transferase P (Fragment) | | 10858.33 | * |
| A0A087X243 | | Glutathione S-transferase P (Fragment) | | 996.04 | * |
| Q5TA02 | | Glutathione-dependent dehydroascorbate reductase (Fragment) | | 1469.15 | * |
| A8MX94 | | GST class-pi | | 21612.53 | 0.386 |
| A0A6Q8PGK1 | | Heat shock 27 kDa protein | | 30162.08 | * |
| H0Y8K0 | | Heat shock 70kDa protein 9B (Mortalin-2)_ isoform CRA_a | | 5920.49 | * |
| C9J3N8 | | Heat shock protein beta-1 | | 19745.92 | * |
| P04792 | | Heat shock protein beta-1 | | 30168.58 | * |
| A0A6Q8PHJ6 | | Heat shock protein beta-1 | | 10422.65 | * |
| A0A6Q8PHA6 | | Heat shock protein beta-1 | | 10416.16 | * |
| A0A6Q8PGY2 | | Heat shock protein beta-1 | | 10416.16 | * |
| A0A6Q8PH65 | | Heat shock protein beta-1 | | 10416.16 | * |
| A0A6Q8PFK8 | | Heat shock protein beta-1 | | 30168.58 | * |
| A0A6Q8PFE7 | | Heat shock protein beta-1 | | 10416.16 | * |
| A0A6Q8PF43 | | Heat shock protein beta-1 | | 10416.16 | * |
| H3BSU0 | | Liver carboxylesterase 1 (Fragment) | | 407.80 | * |
| P55145 | | Mesencephalic astrocyte-derived neurotrophic factor | | 12025.95 | * |
| H7C2D6 | | Mesencephalic astrocyte-derived neurotrophic factor (Fragment) | | 3610.90 | * |
| Q6XQN6 | | Nicotinate phosphoribosyltransferase | | 2556.60 | * |
| G5E977 | | Nicotinate phosphoribosyltransferase | | 2537.61 | * |
| C9J8U2 | | Nicotinate phosphoribosyltransferase | | 1943.98 | * |
| A0A087WUT5 | | Nicotinate phosphoribosyltransferase (Fragment) | | 1708.59 | * |
| A1KZ92 | | Peroxidasin-like protein | | 344.36 | * |
| A6NIW5 | | Peroxiredoxin 2_ isoform CRA_a | | 805.46 | * |
| A0A0A0MRQ5 | | Peroxiredoxin-1 | | 6482.96 | * |
| Q13162 | | Peroxiredoxin-4 | | 8387.69 | * |
| A6NG45 | | Peroxiredoxin-4 | | 666.98 | * |
| A6NJJ0 | | Peroxiredoxin-4 (Fragment) | | 214.46 | * |
| H7C3T4 | | Peroxiredoxin-4 (Fragment) | | 5900.82 | * |
| P30044 | | Peroxiredoxin-5_ mitochondrial | | 1369.47 | * |
| P30041 | | Peroxiredoxin-6 | | 13986.24 | * |
| X6RA14 | | S-formylglutathione hydrolase | | 1536.74 | * |
| P10768 | | S-formylglutathione hydrolase | | 1667.33 | * |
| U3KQT1 | | S-formylglutathione hydrolase | | 1624.87 | * |
| H7BZT7 | | S-formylglutathione hydrolase (Fragment) | | 1494.28 | * |
| A0A3B3ITI4 | | Stress-70 protein_ mitochondrial | | 3204.14 | * |
| P38646 | | Stress-70 protein_ mitochondrial | | 7419.77 | 0.670 |
| A0A7I2V3F7 | | Stress-70 protein_ mitochondrial | | 7419.77 | 0.663 |
| H0Y8S0 | | Stress-70 protein_ mitochondrial (Fragment) | | 1040.56 | 0.594 |
| F5GXD8 | | Stress-induced-phosphoprotein 1 | | 3769.22 | * |
| F5H783 | | Stress-induced-phosphoprotein 1 | | 4270.66 | * |
| P31948 | | Stress-induced-phosphoprotein 1 | | 10056.83 | * |
| H0YGI8 | | Stress-induced-phosphoprotein 1 (Fragment) | | 4616.45 | * |
| P00441 | | Superoxide dismutase [Cu-Zn] | | 8491.53 | * |
| H7BYH4 | | Superoxide dismutase [Cu-Zn] | | 4907.62 | * |
| P10599 | | Thioredoxin | | 7245.89 | * |
| O95881 | | Thioredoxin domain-containing protein 12 | | 1161.27 | * |
| Q16881 | | Thioredoxin reductase 1_ cytoplasmic | | 4239.45 | * |
| A0A087WSY9 | | Thioredoxin reductase 1_ cytoplasmic | | 4239.45 | * |
| A0A0B4J225 | | Thioredoxin reductase 1_ cytoplasmic (Fragment) | | 2849.05 | * |
| E9PIZ5 | | Thioredoxin reductase 1_ cytoplasmic (Fragment) | | 2849.05 | * |
| E9PQI3 | | Thioredoxin reductase 1_ cytoplasmic (Fragment) | | 2784.60 | * |
| E9PLT3 | | Thioredoxin reductase 1_ cytoplasmic (Fragment) | | 2811.60 | * |
| E9PKI4 | | Thioredoxin reductase 1_ cytoplasmic (Fragment) | | 2811.60 | * |
| E9PKD3 | | Thioredoxin reductase 1_ cytoplasmic (Fragment) | | 3555.23 | * |
| P30048 | | Thioredoxin-dependent peroxide reductase_ mitochondrial | | 5641.69 | * |
| E9PIR7 | | Thioredoxin-disulfide reductase | | 4235.96 | * |
| E2QRB9 | | Thioredoxin-disulfide reductase | | 1451.36 | * |
| A0A182DWI3 | | Thioredoxin-disulfide reductase | | 4235.96 | * |
| A0A087WSW9 | | Thioredoxin-disulfide reductase | | 4235.96 | * |
| F8W809 | | Thioredoxin-disulfide reductase | | 4235.96 | * |
|  | | INTERACTION WITH THE ENVIRONMENT | |  |  |
|  | | **Cell motility** | |  |  |
| Q562R1 | | Beta-actin-like protein 2 | | 15804.58 | * |
| A0A087WW53 | | Coronin | | 609.62 | * |
| F5H390 | | Coronin | | 609.62 | * |
| F5H0D2 | | Coronin (Fragment) | | 278.48 | * |
| Q9BR76 | | Coronin-1B | | 1415.13 | * |
| A0A1C7CYX9 | | Dihydropyrimidinase-related protein 2 | | 10607.71 | * |
| Q16555 | | Dihydropyrimidinase-related protein 2 | | 10607.71 | * |
| E5RFU4 | | Dihydropyrimidinase-related protein 2 (Fragment) | | 3428.53 | * |
| A0A2R8YGU1 | | Girdin (Fragment) | | 298.95 | * |
| Q9UIW2 | | Plexin-A1 | | 515.60 | * |
| O75051 | | Plexin-A2 | | 537.05 | * |
| P51805 | | Plexin-A3 | | 487.28 | * |
| Q9HCM2 | | Plexin-A4 | | 539.73 | * |
| K7EJ44 | | Profilin | | 21029.76 | * |
| I3L3D5 | | Profilin (Fragment) | | 2924.76 | * |
| P07737 | | Profilin-1 | | 23549.97 | * |
|  | | **Cell adhesion** | |  |  |
| Q9C0A0 | | Contactin-associated protein-like 4 | | 625.70 | * |
| F5H107 | | Contactin-associated protein-like 4 | | 618.79 | * |
| E9PDN6 | | Contactin-associated protein-like 4 | | 625.70 | * |
| A0A0A0MR20 | | Contactin-associated protein-like 4 | | 624.84 | * |
| A0A087WTA1 | | Contactin-associated protein-like 4 | | 619.64 | * |
| F5H6T4 | | Integrin beta | | 92.62 | * |
| V9GZ57 | | Integrin beta (Fragment) | | 126.09 | * |
| P26010 | | Integrin beta-7 | | 146.56 | * |
| A0A0G2JPP5 | | Protein scribble homolog | | 410.99 | * |
| A0A669KB89 | | Protein scribble homolog | | 410.99 | * |
| A0A0G2JNZ2 | | Protein scribble homolog | | 410.99 | * |
| A0A0G2JMS7 | | Protein scribble homolog | | 401.68 | * |
| Q14160 | | Protein scribble homolog | | 410.99 | * |
| H0YCG0 | | Protein scribble homolog (Fragment) | | 388.99 | * |
| Q9NRJ7 | | Protocadherin beta-16 | | 420.11 | * |
| A0A096LP81 | | Protocadherin beta-16 (Fragment) | | 412.22 | * |
| Q15942 | | Zyxin | | 7665.05 | 0.576 |
| C9JJK5 | | Zyxin (Fragment) | | 251.57 | * |
| C9IZ41 | | Zyxin (Fragment) | | 1106.40 | * |
| H7C3R3 | | Zyxin (Fragment) | | 278.86 | * |
| H7C3D3 | | Zyxin (Fragment) | | 5806.14 | 0.467 |
| SYSTEMIC INTERACTION WITH THE ENVIRONMENT | | | | | |
|  | | | **Muscle contraction** |  |  |
| F8W1I5 | | | Myosin light chain 6B | 951.98 | * |
| P14649 | | | Myosin light chain 6B | 960.31 | * |
| F8W1R7 | | | Myosin light polypeptide 6 | 12055.66 | 0.571 |
| F8W180 | | | Myosin light polypeptide 6 | 10483.03 | 0.571 |
| G3V1Y7 | | | Myosin light polypeptide 6 | 11370.61 | 0.576 |
| G3V1V0 | | | Myosin light polypeptide 6 | 12055.66 | 0.588 |
| J3KND3 | | | Myosin light polypeptide 6 | 12055.66 | 0.571 |
| P60660 | | | Myosin light polypeptide 6 | 12055.66 | 0.410 |
| F8VZU9 | | | Myosin light polypeptide 6 | 10483.03 | 0.571 |
| B7Z6Z4 | | | Myosin light polypeptide 6 | 12055.66 | 0.406 |
| F8VXL3 | | | Myosin light polypeptide 6 | 7928.26 | * |
| G8JLA2 | | | Myosin light polypeptide 6 | 12055.66 | 0.571 |
| F8VPF3 | | | Myosin light polypeptide 6 (Fragment) | 11168.08 | 0.406 |
| H0YI43 | | | Myosin light polypeptide 6 (Fragment) | 10483.03 | * |
| P35749 | | | Myosin-11 | 145.41 | * |
| A0A0C4DFM8 | | | Myosin-14 | 328.34 | * |
| A0A5F9UN72 | | | Tropomyosin 4_ isoform CRA_c | 10451.60 | * |
| A0A494C0V8 | | | Tropomyosin alpha-1 chain | 2321.22 | * |
| H0YL42 | | | Tropomyosin alpha-1 chain (Fragment) | 2321.22 | * |
| K7EPB9 | | | Tropomyosin alpha-4 chain (Fragment) | 9361.40 | * |
| U3KQK2 | | | Tropomyosin beta chain (Fragment) | 1899.29 | * |
|  | | | **Immune response** |  |  |
| G5E9S7 | | | Actin related protein 2/3 complex_ subunit 2_ 34kDa_ isoform CRA_e | 475.38 | * |
| P63261 | | | Actin_ cytoplasmic 2 | 43091.04 | 0.004 |
| I3L4N8 | | | Actin_ cytoplasmic 2 | 43091.04 | 0.013 |
| I3L3I0 | | | Actin_ cytoplasmic 2 (Fragment) | 19926.16 | 0.004 |
| I3L1U9 | | | Actin_ cytoplasmic 2 (Fragment) | 19926.16 | 0.007 |
| F8WDR7 | | | Actin-like protein 3 (Fragment) | 23355.66 | * |
| A0A1B0GWH0 | | | Actin-related protein 2 | 1732.37 | * |
| A0A1B0GWD0 | | | Actin-related protein 2 | 1732.37 | * |
| C9K057 | | | Actin-related protein 2/3 complex subunit 1B | 8234.91 | 0.631 |
| O15143 | | | Actin-related protein 2/3 complex subunit 1B | 8740.81 | 0.631 |
| F8VXW2 | | | Actin-related protein 2/3 complex subunit 1B | 6377.83 | 0.618 |
| C9JEY1 | | | Actin-related protein 2/3 complex subunit 1B (Fragment) | 8234.91 | 0.631 |
| C9JBJ7 | | | Actin-related protein 2/3 complex subunit 1B (Fragment) | 6377.83 | 0.618 |
| C9JTT6 | | | Actin-related protein 2/3 complex subunit 1B (Fragment) | 5367.82 | 0.625 |
| O15144 | | | Actin-related protein 2/3 complex subunit 2 | 3617.00 | * |
| O15145 | | | Actin-related protein 2/3 complex subunit 3 | 17431.37 | * |
| F8VR50 | | | Actin-related protein 2/3 complex subunit 3 (Fragment) | 14321.86 | * |
| C9JZD1 | | | Actin-related protein 2/3 complex subunit 3 (Fragment) | 16528.14 | * |
| R4GN08 | | | Actin-related protein 2/3 complex subunit 4 (Fragment) | 1106.88 | * |
| O15511 | | | Actin-related protein 2/3 complex subunit 5 | 3627.45 | * |
| B1ALC0 | | | Actin-related protein 2/3 complex subunit 5 | 1050.85 | * |
| F8WEW2 | | | Actin-related protein 3 | 9712.19 | * |
| F8WE84 | | | Actin-related protein 3 | 9712.19 | * |
| E9PHT9 | | | Annexin | 24068.45 | 0.084 |
| D6RBL5 | | | Annexin | 13850.32 | 0.650 |
| D6RBE9 | | | Annexin | 16804.13 | 0.650 |
| Q5T3N1 | | | Annexin (Fragment) | 16307.94 | * |
| Q5T3N0 | | | Annexin (Fragment) | 11929.89 | * |
| P04083 | | | Annexin A1 | 18072.62 | * |
| P08758 | | | Annexin A5 | 30602.12 | 0.644 |
| D6RCN3 | | | Annexin A5 | 10270.46 | * |
| O43150 | | | Arf-GAP with SH3 domain_ ANK repeat and PH domain-containing protein 2 | 594.14 | * |
| A0A286YFG8 | | | Arf-GAP with SH3 domain_ ANK repeat and PH domain-containing protein 2 (Fragment) | 793.06 | * |
| F5H6T1 | | | ARP2 actin-related protein 2 homolog (Yeast)_ isoform CRA_d | 2792.59 | * |
| G5E9J0 | | | Arp2/3 complex 34 kDa subunit | 475.38 | * |
| C9JTV5 | | | Arp2/3 complex 34 kDa subunit (Fragment) | 475.38 | * |
| H7C3F9 | | | Arp2/3 complex 34 kDa subunit (Fragment) | 3442.62 | * |
| P61769 | | | Beta-2-microglobulin | 3926.66 | * |
| F5H6I0 | | | Beta-2-microglobulin | 3926.66 | * |
| H0YLF3 | | | Beta-2-microglobulin (Fragment) | 3926.66 | * |
| Q9H078 | | | Caseinolytic peptidase B protein homolog | 521.33 | * |
| A0A7I2YQK9 | | | Cathepsin B | 2448.11 | * |
| A0A7P0NGZ6 | | | Cathepsin B | 4230.87 | * |
| E9PHZ5 | | | Cathepsin B | 6229.65 | * |
| P07858 | | | Cathepsin B | 6229.65 | * |
| E9PSG5 | | | Cathepsin B | 6229.65 | * |
| A0A7I2V668 | | | Cathepsin B | 6229.65 | * |
| E9PR21 | | | Cathepsin B | 1279.54 | * |
| E9PQM1 | | | Cathepsin B | 449.32 | * |
| A0A7I2V4Z9 | | | Cathepsin B | 6229.65 | * |
| A0A7I2V4S6 | | | Cathepsin B | 294.99 | * |
| A0A7I2V440 | | | Cathepsin B | 6042.11 | * |
| A0A7I2V367 | | | Cathepsin B | 2153.12 | * |
| E9PNL5 | | | Cathepsin B (Fragment) | 294.99 | * |
| E9PLY3 | | | Cathepsin B (Fragment) | 294.99 | * |
| E9PKQ7 | | | Cathepsin B (Fragment) | 606.37 | * |
| R4GMQ5 | | | Cathepsin B (Fragment) | 5780.33 | * |
| A0A7P0TAQ0 | | | Cathepsin S | 1155.57 | * |
| A0A7P0T8U1 | | | Cathepsin S | 680.63 | * |
| A0A7P0T904 | | | Cathepsin S | 1101.80 | * |
| A0A7P0T8H4 | | | Cathepsin S | 680.63 | * |
| A0A7P0T844 | | | Cathepsin S | 1097.97 | * |
| U3KQE7 | | | Cathepsin S | 680.63 | * |
| P25774 | | | Cathepsin S | 1155.57 | * |
| U3KPS4 | | | Cathepsin S (Fragment) | 474.94 | * |
| P36222 | | | Chitinase-3-like protein 1 | 1597.86 | * |
| H0Y3U8 | | | Chitinase-3-like protein 1 (Fragment) | 1024.24 | * |
| Q9Y281 | | | Cofilin-2 | 8142.94 | * |
| A6NC98 | | | Coiled-coil domain-containing protein 88B | 210.23 | * |
| I3L3Q7 | | | Complement component 1 Q subcomponent-binding protein_ mitochondrial (Fragment) | 4992.80 | * |
| I3L3B0 | | | Complement component 1 Q subcomponent-binding protein_ mitochondrial | 4992.80 | * |
| Q07021 | | | Complement component 1 Q subcomponent-binding protein_ mitochondrial | 7230.48 | * |
| A0A7P0TB36 | | | Endoplasmic reticulum chaperone BiP | 21012.03 | 0.582 |
| A0A7P0TAI0 | | | Endoplasmic reticulum chaperone BiP | 18584.81 | 0.582 |
| A0A0G2JHL1 | | | G patch domain and ankyrin repeat-containing protein 1 | 740.83 | * |
| O95872 | | | G patch domain and ankyrin repeat-containing protein 1 | 740.83 | * |
| P09382 | | | Galectin-1 | 15197.19 | * |
| P04899 | | | Guanine nucleotide-binding protein G(i) subunit alpha-2 | 397.29 | * |
| P34931 | | | Heat shock 70 kDa protein 1-like | 7162.77 | * |
| A8K7Q2 | | | Heat shock cognate 71 kDa protein | 14449.51 | 0.644 |
| P11142 | | | Heat shock cognate 71 kDa protein | 44297.06 | 0.650 |
| E9PS65 | | | Heat shock cognate 71 kDa protein (Fragment) | 10698.04 | 0.650 |
| E9PN89 | | | Heat shock cognate 71 kDa protein (Fragment) | 24043.66 | 0.136 |
| P54652 | | | Heat shock-related 70 kDa protein 2 | 11903.81 | 0.001 |
| P09429 | | | High mobility group protein B1 | 1668.83 | * |
| Q5T7C4 | | | High mobility group protein B1 | 1650.26 | * |
| A0A3B3IS11 | | | Histone H2A | 3271.32 | * |
| C9J386 | | | Histone H2A | 5081.07 | * |
| C9J0D1 | | | Histone H2A | 7828.83 | * |
| A0A0U1RRH7 | | | Histone H2A | 8902.71 | * |
| A0A0U1RR32 | | | Histone H2A | 8902.71 | * |
| A0A494C189 | | | Histone H2A | 7828.83 | * |
| H0YFX9 | | | Histone H2A (Fragment) | 6154.95 | * |
| P0C0S8 | | | Histone H2A type 1 | 8902.71 | * |
| Q96QV6 | | | Histone H2A type 1-A | 3271.32 | * |
| P04908 | | | Histone H2A type 1-B/E | 8902.71 | * |
| Q93077 | | | Histone H2A type 1-C | 8902.71 | * |
| P20671 | | | Histone H2A type 1-D | 8902.71 | * |
| Q96KK5 | | | Histone H2A type 1-H | 8902.71 | * |
| Q99878 | | | Histone H2A type 1-J | 8902.71 | * |
| Q6FI13 | | | Histone H2A type 2-A | 8902.71 | * |
| Q8IUE6 | | | Histone H2A type 2-B | 2747.75 | * |
| Q16777 | | | Histone H2A type 2-C | 8902.71 | * |
| Q9BTM1 | | | Histone H2A.J | 8902.71 | * |
| Q71UI9 | | | Histone H2A.V | 8676.91 | * |
| P0C0S5 | | | Histone H2A.Z | 8676.91 | * |
| P16104 | | | Histone H2AX | 3273.08 | * |
| U3KQK0 | | | Histone H2B | 16807.41 | * |
| Q96A08 | | | Histone H2B type 1-A | 7130.46 | * |
| P33778 | | | Histone H2B type 1-B | 17593.68 | * |
| P62807 | | | Histone H2B type 1-C/E/F/G/I | 16807.41 | * |
| P58876 | | | Histone H2B type 1-D | 16807.41 | * |
| Q93079 | | | Histone H2B type 1-H | 16807.41 | * |
| P06899 | | | Histone H2B type 1-J | 17593.68 | * |
| O60814 | | | Histone H2B type 1-K | 16807.41 | * |
| Q99880 | | | Histone H2B type 1-L | 16807.41 | * |
| Q99879 | | | Histone H2B type 1-M | 16807.41 | * |
| Q99877 | | | Histone H2B type 1-N | 16807.41 | * |
| P23527 | | | Histone H2B type 1-O | 17593.68 | * |
| Q16778 | | | Histone H2B type 2-E | 17593.68 | * |
| A0A2R8Y619 | | | Histone H2B type 2-E1 | 311.58 | * |
| Q5QNW6 | | | Histone H2B type 2-F | 16807.41 | * |
| Q8N257 | | | Histone H2B type 3-B | 17593.68 | * |
| P57053 | | | Histone H2B type F-S | 16800.03 | * |
| Q6NXT2 | | | Histone H3.3C | 3853.37 | * |
| P62805 | | | Histone H4 | 7844.59 | * |
| Q53FA3 | | | HSPA1L (Fragment) | 7162.77 | 0.001 |
| D3DSM0 | | | Integrin beta | 2644.32 | * |
| A0A494C0X7 | | | Integrin beta | 2644.32 | * |
| E7EVZ9 | | | Integrin beta (Fragment) | 2349.88 | * |
| J3KNI6 | | | Integrin beta (Fragment) | 2487.44 | * |
| E5RK25 | | | Integrin beta (Fragment) | 2349.88 | * |
| P05107 | | | Integrin beta-2 | 2518.87 | * |
| E5RK54 | | | Integrin beta-2 (Fragment) | 2250.55 | * |
| E5RIG7 | | | Integrin beta-2 (Fragment) | 2349.88 | * |
| E5RHE6 | | | Integrin beta-2 (Fragment) | 2250.55 | * |
| Q14624 | | | Inter-alpha-trypsin inhibitor heavy chain H4 | 590.15 | * |
| H7C0L5 | | | Inter-alpha-trypsin inhibitor heavy chain H4 (Fragment) | 551.63 | * |
| B7ZKJ8 | | | ITIH4 protein | 590.15 | * |
| P33241 | | | Lymphocyte-specific protein 1 | 820.58 | * |
| C9JKF7 | | | Lymphocyte-specific protein 1 (Fragment) | 728.40 | * |
| E9PBD8 | | | Lymphocyte-specific protein 1 (Fragment) | 758.95 | * |
| E7EMG9 | | | Lymphocyte-specific protein 1 (Fragment) | 758.95 | * |
| C9JU59 | | | Lymphocyte-specific protein 1 (Fragment) | 784.63 | * |
| P14174 | | | Macrophage migration inhibitory factor | 4042.46 | * |
| A0A0U1RQL8 | | | Macrophage-capping protein (Fragment) | 2540.74 | * |
| Q5T0I0 | | | Macrophage-capping protein (Fragment) | 4675.23 | * |
| Q99558 | | | Mitogen-activated protein kinase kinase kinase 14 | 552.28 | 0.506 |
| V9GZ54 | | | Moesin (Fragment) | 5010.75 | * |
| Q86W28 | | | NACHT_ LRR and PYD domains-containing protein 8 | 558.22 | * |
| P30414 | | | NK-tumor recognition protein | 361.90 | * |
| C9J5S7 | | | Peptidyl-prolyl cis-trans isomerase | 39560.98 | * |
| F8WE65 | | | Peptidyl-prolyl cis-trans isomerase | 39560.98 | * |
| A0A7I2V4V1 | | | Peptidyl-prolyl cis-trans isomerase | 25807.85 | * |
| P62937 | | | Peptidyl-prolyl cis-trans isomerase A | 46788.33 | * |
| A0A7P0S768 | | | Peptidyl-prolyl cis-trans isomerase A | 1667.07 | * |
| A0A7I2V5J5 | | | Peptidyl-prolyl cis-trans isomerase A | 12560.83 | * |
| E5RIZ5 | | | Peptidyl-prolyl cis-trans isomerase A | 1667.07 | * |
| P26447 | | | Protein S100-A4 | 2163.83 | * |
| Q96E17 | | | Ras-related protein Rab-3C | 1115.57 | * |
| A0A5F9ZHH6 | | | Retinoic acid receptor RXR-alpha | 654.98 | * |
| P19793 | | | Retinoic acid receptor RXR-alpha | 654.98 | * |
| P28702 | | | Retinoic acid receptor RXR-beta | 1177.33 | * |
| A0A0G2JKR7 | | | Retinoic acid receptor RXR-beta | 584.82 | * |
| P48443 | | | Retinoic acid receptor RXR-gamma | 630.23 | * |
| A0A087WZ88 | | | Retinoic acid receptor RXR-gamma | 630.23 | * |
| O00584 | | | Ribonuclease T2 | 1115.63 | * |
| D6REQ6 | | | Ribonuclease T2 | 1188.33 | * |
| A0A087WZM2 | | | Ribonuclease T2 | 1115.63 | * |
| D6RHI9 | | | Ribonuclease T2 (Fragment) | 1115.63 | * |
| Q5T7I8 | | | SHC-transforming protein 3 | 705.25 | * |
| Q92529 | | | SHC-transforming protein 3 | 826.62 | * |
| H0Y858 | | | Toll-like receptor 9 (Fragment) | 624.89 | * |
| A0A7I2YQL8 | | | Transforming growth factor beta | 615.74 | * |
| A0A499FJK2 | | | Transforming growth factor beta | 618.77 | * |
| P01137 | | | Transforming growth factor beta-1 proprotein | 618.77 | * |
| A0A7I2V5Z9 | | | Transforming growth factor beta-1 proprotein | 615.74 | * |
| A0A494C0A3 | | | Tripeptidyl-peptidase 2 | 297.38 | * |
|  | | | CELL FATE |  |  |
|  | | | **Cell growth/morphogenesis** |  |  |
| F8WBW6 | | | 28 kDa heat- and acid-stable phosphoprotein | 2964.96 | * |
| Q13442 | | | 28 kDa heat- and acid-stable phosphoprotein | 3390.79 | * |
| F8VR77 | | | Proliferation-associated protein 2G4 | 1930.99 | * |
| Q9UQ80 | | | Proliferation-associated protein 2G4 | 2283.23 | * |
| F8W0A3 | | | Proliferation-associated protein 2G4 (Fragment) | 1855.79 | * |
| F8VZ69 | | | Proliferation-associated protein 2G4 (Fragment) | 1409.37 | * |
|  | | | **Cell differentiation** |  |  |
| E9PS42 | | | Cysteine and glycine-rich protein 1 | 3539.93 | * |
| E9PP21 | | | Cysteine and glycine-rich protein 1 | 1901.26 | * |
| P21291 | | | Cysteine and glycine-rich protein 1 | 3580.14 | * |
| E9PND2 | | | Cysteine and glycine-rich protein 1 (Fragment) | 3326.21 | * |
| C9JL85 | | | Myotrophin | 5007.69 | * |
| P58546 | | | Myotrophin | 5007.69 | * |
| Q9UMX5 | | | Neudesin | 2756.47 | * |
| Q92882 | | | Osteoclast-stimulating factor 1 | 1218.25 | * |
| F8W122 | | | Phosphatidylinositol phosphatase PTPRQ (Fragment) | 2772.02 | * |
| F8VXI2 | | | Phosphatidylinositol phosphatase PTPRQ (Fragment) | 2765.23 | * |
| F8VW52 | | | Phosphatidylinositol phosphatase PTPRQ (Fragment) | 2760.86 | * |
| A0A087X0B9 | | | Protein-tyrosine-phosphatase | 2689.15 | * |
| A0A087WZU1 | | | Protein-tyrosine-phosphatase | 2668.97 | * |
| Q9Y6N7 | | | Roundabout homolog 1 | 562.02 | * |
| A0A087WTM1 | | | Roundabout homolog 1 | 562.02 | * |
| H0YCX0 | | | Translationally-controlled tumor protein (Fragment) | 457.44 | * |
|  | | | **Cell aging** |  |  |
| P10244 | | | Myb-related protein B | 288.81 | * |
|  | | | **Cell death** |  |  |
| F8WCQ3 | | | Death-associated protein kinase 1 | 258.66 | * |
| P53355 | | | Death-associated protein kinase 1 | 391.93 | * |
| P02792 | | | Ferritin light chain | 1044.93 | * |
| D6RB20 | | | Prelamin-A/C | 2348.04 | * |
| B8ZZQ6 | | | Prothymosin alpha | 1063.87 | 0.677 |
| P06454 | | | Prothymosin alpha | 1063.87 | 0.229 |
| H7C2N1 | | | Prothymosin alpha (Fragment) | 1035.11 | 0.657 |
| BIOGENESIS OF CELLULAR COMPONENTS | | | | | |
|  | | | **Cytoskeleton/structural proteins** |  |  |
| E7ESP9 | | | 160 kDa neurofilament protein | 358.46 | * |
| E7EMV2 | | | 160 kDa neurofilament protein | 339.00 | * |
| E7EVS6 | | | Actin_ cytoplasmic 1 | 38581.02 | * |
| A0A6Q8PFE4 | | | Actin_ cytoplasmic 1 | 29101.30 | 0.004 |
| P60709 | | | Actin_ cytoplasmic 1 | 43091.04 | 0.004 |
| A0A2R8Y793 | | | Actin_ cytoplasmic 1 (Fragment) | 38581.02 | 0.004 |
| A0A2R8YFE2 | | | Actin_ cytoplasmic 1 (Fragment) | 4510.02 | * |
| A0A2R8YEA7 | | | Actin_ cytoplasmic 1 (Fragment) | 14258.08 | 0.004 |
| F6RFD5 | | | Actin-depolymerizing factor | 364.09 | * |
| H9KV75 | | | Alpha-actinin-1 | 8761.10 | * |
| P12814 | | | Alpha-actinin-1 | 16303.51 | 0.058 |
| G3V5M4 | | | Alpha-actinin-1 (Fragment) | 8402.25 | 0.236 |
| G3V2X9 | | | Alpha-actinin-1 (Fragment) | 3380.78 | * |
| G3V2W4 | | | Alpha-actinin-1 (Fragment) | 3938.18 | * |
| G3V380 | | | Alpha-actinin-1 (Fragment) | 5412.84 | 0.236 |
| G3V2N5 | | | Alpha-actinin-1 (Fragment) | 11027.22 | 0.232 |
| G3V2E8 | | | Alpha-actinin-1 (Fragment) | 2989.41 | * |
| H0YJW3 | | | Alpha-actinin-1 (Fragment) | 2158.55 | * |
| H0YJ11 | | | Alpha-actinin-1 (Fragment) | 2228.30 | * |
| H7C5W8 | | | Alpha-actinin-1 (Fragment) | 3970.82 | * |
| P35609 | | | Alpha-actinin-2 | 7527.08 | * |
| A0A494C166 | | | Alpha-actinin-2 | 5941.26 | * |
| A0A494C0Q3 | | | Alpha-actinin-2 | 5838.99 | * |
| A0A494C033 | | | Alpha-actinin-2 | 5872.02 | * |
| A0A494C1A0 | | | Alpha-actinin-2 (Fragment) | 7527.08 | * |
| A0A494C060 | | | Alpha-actinin-2 (Fragment) | 1510.45 | * |
| A0A087WSZ2 | | | Alpha-actinin-3 | 6780.90 | * |
| Q16352 | | | Alpha-internexin | 332.34 | * |
| H3BT58 | | | Coactosin-like protein | 6834.72 | * |
| Q14019 | | | Coactosin-like protein | 12529.53 | * |
| E9PK25 | | | Cofilin_ non-muscle isoform | 106329.80 | * |
| G3V1A4 | | | Cofilin_ non-muscle isoform | 106303.30 | * |
| E9PS23 | | | Cofilin_ non-muscle isoform (Fragment) | 104388.30 | * |
| E9PQB7 | | | Cofilin_ non-muscle isoform (Fragment) | 104373.50 | * |
| E9PP50 | | | Cofilin_ non-muscle isoform (Fragment) | 106211.20 | * |
| E9PLJ3 | | | Cofilin_ non-muscle isoform (Fragment) | 104373.50 | * |
| P23528 | | | Cofilin-1 | 106348.10 | * |
| B4E3S0 | | | Coronin | 2596.64 | * |
| H3BRY3 | | | Coronin | 460.78 | * |
| F8VUX3 | | | Coronin | 1562.19 | * |
| F8VSA4 | | | Coronin (Fragment) | 1562.19 | * |
| F8VRE9 | | | Coronin (Fragment) | 1562.19 | * |
| F8W1H8 | | | Coronin (Fragment) | 1562.19 | * |
| H3BU76 | | | Coronin (Fragment) | 255.22 | * |
| H3BTU6 | | | Coronin (Fragment) | 443.15 | * |
| H3BSL1 | | | Coronin (Fragment) | 255.22 | * |
| H3BRJ0 | | | Coronin (Fragment) | 255.22 | * |
| H3BNA2 | | | Coronin (Fragment) | 255.22 | * |
| F8VVB7 | | | Coronin (Fragment) | 1562.19 | * |
| F8VV53 | | | Coronin (Fragment) | 1606.21 | * |
| F8VTT6 | | | Coronin (Fragment) | 1562.19 | * |
| P31146 | | | Coronin-1A | 2490.15 | 0.600 |
| Q9ULV4 | | | Coronin-1C | 2596.64 | * |
| H0YHL7 | | | Coronin-1C (Fragment) | 990.44 | * |
| P17661 | | | Desmin | 446.01 | * |
| P60981 | | | Destrin | 376.53 | * |
| Q14195 | | | Dihydropyrimidinase-related protein 3 | 2128.33 | * |
| H0YBT4 | | | Dihydropyrimidinase-related protein 3 (Fragment) | 2091.70 | * |
| D6RCR4 | | | Drebrin (Fragment) | 544.57 | * |
| Q16658 | | | Fascin | 6693.36 | * |
| C9JPH9 | | | Fascin (Fragment) | 261.77 | * |
| A0A0A0MSB2 | | | Fascin (Fragment) | 284.07 | * |
| C9JFC0 | | | Fascin (Fragment) | 284.07 | * |
| P60983 | | | Glia maturation factor beta | 1902.26 | * |
| G3V4P8 | | | Glia maturation factor beta (Fragment) | 1875.13 | * |
| G3V3J6 | | | HCG1983504_ isoform CRA_b | 3779.34 | * |
| G3V3R4 | | | HCG1983504_ isoform CRA_c | 7956.89 | * |
| G3V2N6 | | | HCG1983504_ isoform CRA_d | 8015.94 | * |
| G3V2R8 | | | HCG1983504_ isoform CRA_e | 7956.89 | * |
| A0A6Q8PGJ2 | | | Intraflagellar transport protein 172 homolog | 999.61 | * |
| Q9UG01 | | | Intraflagellar transport protein 172 homolog | 999.61 | * |
| Q8IYT4 | | | Katanin p60 ATPase-containing subunit A-like 2 | 1085.06 | * |
| K7EM02 | | | Katanin p60 ATPase-containing subunit A-like 2 (Fragment) | 1060.71 | * |
| K7EIJ8 | | | Katanin p60 ATPase-containing subunit A-like 2 (Fragment) | 1060.71 | * |
| A0A1B0GVI3 | | | Keratin_ type I cytoskeletal 10 | 474.68 | * |
| P13645 | | | Keratin_ type I cytoskeletal 10 | 474.68 | * |
| E7EVA0 | | | Microtubule-associated protein | 1433.80 | * |
| P27816 | | | Microtubule-associated protein 4 | 1405.98 | * |
| A0A0J9YVV8 | | | Microtubule-associated protein 4 (Fragment) | 1290.69 | * |
| A0A0J9YW37 | | | Microtubule-associated protein 4 (Fragment) | 1290.69 | * |
| H7C456 | | | Microtubule-associated protein 4 (Fragment) | 1304.76 | * |
| E9PJZ0 | | | Neuroblast differentiation-associated  protein AHNAK (Fragment) | 1160.71 | 0.501 |
| E9PJC6 | | | Neuroblast differentiation-associated protein AHNAK | 1160.71 | 0.511 |
| E9PQE3 | | | Neuroblast differentiation-associated protein AHNAK (Fragment) | 1146.96 | 0.501 |
| E9PLK4 | | | Neuroblast differentiation-associated protein AHNAK (Fragment) | 1160.71 | 0.501 |
| E9PKR9 | | | Neuroblast differentiation-associated protein AHNAK (Fragment) | 1160.71 | 0.491 |
| P12036 | | | Neurofilament heavy polypeptide | 263.87 | * |
| P07196 | | | Neurofilament light polypeptide | 313.13 | * |
| P07197 | | | Neurofilament medium polypeptide | 358.46 | * |
| Q9BYX7 | | | Putative beta-actin-like protein 3 | 6458.71 | * |
| P35241 | | | Radixin | 1067.46 | * |
| A0A2R8Y7M3 | | | Radixin | 1047.35 | * |
| A0A2R8Y5S7 | | | Radixin | 1067.46 | * |
| E9PNP4 | | | Radixin | 548.50 | * |
| A0A2R8Y5P0 | | | Radixin (Fragment) | 593.35 | * |
| E9PQ82 | | | Radixin (Fragment) | 442.05 | * |
| M0QZQ3 | | | Spectrin beta chain | 573.04 | * |
| C9JY79 | | | Spectrin beta chain | 580.51 | * |
| A0A087WUZ3 | | | Spectrin beta chain | 602.63 | * |
| P11277 | | | Spectrin beta chain_ erythrocytic | 533.15 | * |
| Q01082 | | | Spectrin beta chain_ non-erythrocytic 1 | 592.17 | * |
| F8W6C1 | | | Spectrin beta chain_ non-erythrocytic 1 (Fragment) | 486.77 | * |
| E9PJZ2 | | | Spectrin beta chain_ non-erythrocytic 2  (Fragment) | 603.55 | * |
| O15020 | | | Spectrin beta chain_ non-erythrocytic 2 | 756.45 | * |
| Q9H254 | | | Spectrin beta chain_ non-erythrocytic 4 | 580.51 | * |
| A4QPE4 | | | SPTBN2 protein | 688.92 | * |
| A0A494C034 | | | Tropomyosin alpha-3 chain (Fragment) | 573.69 | * |
| F5H5D3 | | | Tubulin alpha chain | 30863.15 | * |
| F8VQQ4 | | | Tubulin alpha chain (Fragment) | 28925.89 | * |
| C9J2C0 | | | Tubulin alpha chain (Fragment) | 1123.57 | * |
| C9JJQ8 | | | Tubulin alpha chain (Fragment) | 368.12 | * |
| C9JEV8 | | | Tubulin alpha chain (Fragment) | 368.12 | * |
| C9JQ00 | | | Tubulin alpha chain (Fragment) | 368.12 | * |
| F8W0F6 | | | Tubulin alpha-1A chain | 2495.29 | * |
| A0A7P0Z4A1 | | | Tubulin alpha-1A chain | 2495.29 | * |
| Q71U36 | | | Tubulin alpha-1A chain | 31013.09 | * |
| F8VRZ4 | | | Tubulin alpha-1A chain (Fragment) | 28557.78 | * |
| P68363 | | | Tubulin alpha-1B chain | 31118.12 | * |
| F8VRK0 | | | Tubulin alpha-1B chain (Fragment) | 28557.78 | * |
| F8VWV9 | | | Tubulin alpha-1B chain (Fragment) | 28557.78 | * |
| F8VX09 | | | Tubulin alpha-1B chain (Fragment) | 28557.78 | * |
| F8VVB9 | | | Tubulin alpha-1B chain (Fragment) | 29059.73 | * |
| F8VS66 | | | Tubulin alpha-1C chain | 28557.78 | * |
| A0A1W2PQM2 | | | Tubulin alpha-1C chain | 30455.04 | * |
| Q9BQE3 | | | Tubulin alpha-1C chain | 30863.15 | * |
| P0DPH7 | | | Tubulin alpha-3C chain | 4905.24 | * |
| P0DPH8 | | | Tubulin alpha-3D chain | 4905.24 | * |
| Q6PEY2 | | | Tubulin alpha-3E chain | 4610.08 | * |
| P68366 | | | Tubulin alpha-4A chain | 2094.78 | * |
| C9JDS9 | | | Tubulin alpha-4A chain (Fragment) | 870.08 | * |
| Q9NY65 | | | Tubulin alpha-8 chain | 1202.83 | * |
| A0A7P0T945 | | | Tubulin alpha-8 chain | 1202.83 | * |
| V9GZ17 | | | Tubulin alpha-8 chain (Fragment) | 1119.67 | * |
| A6NNZ2 | | | Tubulin beta 8B | 757.47 | * |
| Q5ST81 | | | Tubulin beta chain | 332.40 | * |
| P07437 | | | Tubulin beta chain | 8289.30 | * |
| Q5JP53 | | | Tubulin beta chain | 4509.95 | * |
| Q5SQY0 | | | Tubulin beta chain | 757.47 | * |
| K7ESM5 | | | Tubulin beta chain (Fragment) | 510.54 | * |
| G3V2A3 | | | Tubulin beta chain (Fragment) | 700.15 | * |
| M0R042 | | | Tubulin beta chain (Fragment) | 3881.02 | * |
| Q9H4B7 | | | Tubulin beta-1 chain | 762.81 | * |
| Q13885 | | | Tubulin beta-2A chain | 8336.82 | * |
| Q9BVA1 | | | Tubulin beta-2B chain | 8336.82 | * |
| G3V5W4 | | | Tubulin beta-3 chain | 7956.89 | * |
| G3V4U2 | | | Tubulin beta-3 chain | 3779.34 | * |
| G3V3W7 | | | Tubulin beta-3 chain | 3779.34 | * |
| Q13509 | | | Tubulin beta-3 chain | 8303.50 | * |
| P04350 | | | Tubulin beta-4A chain | 6010.50 | * |
| M0QY37 | | | Tubulin beta-4A chain | 5575.72 | * |
| M0QX14 | | | Tubulin beta-4A chain | 5575.72 | * |
| M0R2T4 | | | Tubulin beta-4A chain | 3862.69 | * |
| M0R0X0 | | | Tubulin beta-4A chain | 5575.72 | * |
| M0QZL7 | | | Tubulin beta-4A chain (Fragment) | 5575.72 | * |
| M0QYM7 | | | Tubulin beta-4A chain (Fragment) | 3881.02 | * |
| M0QY85 | | | Tubulin beta-4A chain (Fragment) | 5575.72 | * |
| M0R2D3 | | | Tubulin beta-4A chain (Fragment) | 3954.32 | * |
| M0R278 | | | Tubulin beta-4A chain (Fragment) | 5575.72 | * |
| P68371 | | | Tubulin beta-4B chain | 5845.29 | * |
| K7ESQ3 | | | Tubulin beta-6 chain | 720.43 | * |
| K7ERA8 | | | Tubulin beta-6 chain | 720.43 | * |
| K7EQT3 | | | Tubulin beta-6 chain | 720.43 | * |
| K7EPE5 | | | Tubulin beta-6 chain | 720.43 | * |
| K7EN98 | | | Tubulin beta-6 chain | 720.43 | * |
| K7EJZ4 | | | Tubulin beta-6 chain | 720.43 | * |
| K7EJ64 | | | Tubulin beta-6 chain | 720.43 | * |
| Q9BUF5 | | | Tubulin beta-6 chain | 1240.52 | * |
| K7EL29 | | | Tubulin beta-6 chain (Fragment) | 720.43 | * |
| A0A075B724 | | | Tubulin beta-8 chain | 1877.65 | * |
| Q3ZCM7 | | | Tubulin beta-8 chain | 1890.64 | * |
| B0YJC5 | | | Vimentin | 1024.20 | * |
| B0YJC4 | | | Vimentin | 1892.98 | * |
| P08670 | | | Vimentin | 1931.88 | * |
| O75083 | | | WD repeat-containing protein 1 | 9380.71 | * |
| D6RD66 | | | WD repeat-containing protein 1 (Fragment) | 5190.62 | * |
|  | | | **Golgi** |  |  |
| A0A087WVI0 | | | Conserved oligomeric Golgi complex subunit 1 | 1217.05 | * |
| Q8WTW3 | | | Conserved oligomeric Golgi complex subunit 1 | 1223.96 | * |
| J3KRP4 | | | Conserved oligomeric Golgi complex subunit 1 (Fragment) | 542.00 | * |
| E9PBL8 | | | Conserved oligomeric Golgi complex subunit 1 (Fragment) | 1217.05 | * |
|  | | | SUBCELLULAR LOCALIZATION |  |  |
|  | | | **Cytoplasm** |  |  |
| Q9UKY7 | | | Protein CDV3 homolog | 6332.00 | * |
| D6RFH2 | | | Protein CDV3 homolog | 6244.63 | * |
| D6RDN0 | | | Protein CDV3 homolog | 225.53 | * |
| D6R9V8 | | | Protein CDV3 homolog | 6244.63 | * |
| H0Y8K3 | | | Protein CDV3 homolog (Fragment) | 6437.91 | * |
| D6RAV0 | | | Protein CDV3 homolog (Fragment) | 6155.88 | * |
|  | | | **Centrosome** |  |  |
| A6NIR2 | | | Chromosome 1 open reading frame 41_ isoform CRA_b | 823.51 | * |
| Q9Y547 | | | Intraflagellar transport protein 25 homolog | 1453.69 | * |
| X6R7Y7 | | | Intraflagellar transport protein 25 homolog | 823.51 | * |
|  | | | **Endoplasmic** **reticulum** |  |  |
| Q9Y2B0 | | | Protein canopy homolog 2 | 4603.56 | * |
| F8W1K5 | | | Protein canopy homolog 2 (Fragment) | 4390.19 | * |
| H0YIH9 | | | Protein canopy homolog 2 (Fragment) | 394.22 | * |
| H0YI18 | | | Protein canopy homolog 2 (Fragment) | 425.97 | * |
| F8VXJ7 | | | Protein canopy homolog 2 (Fragment) | 4603.56 | * |
|  | | | **Mitochondrion** |  |  |
| A3KMH1 | | | von Willebrand factor A domain-containing protein 8 | 593.56 | * |
|  | | | **Extracellular / secretion proteins** |  |  |
| P05997 | | | Collagen alpha-2(V) chain | 715.26 | * |
| A0A3B3IRH9 | | | Collagen alpha-2(V) chain (Fragment) | 629.39 | * |
|  | | | UNCLASSIFIED |  |  |
| S4R3N1 | | | 10 kDa heat shock protein_ mitochondrial | 21059.43 | * |
| O95626 | | | Acidic leucine-rich nuclear phosphoprotein 32 family member D | 320.89 | * |
| Q8WXE0 | | | Caskin-2 | 627.55 | * |
| A0A0A0MTQ1 | | | Centrosomal protein of 95 kDa | 773.20 | * |
| Q96GE4 | | | Centrosomal protein of 95 kDa | 774.55 | * |
| I3L3A3 | | | Clustered mitochondria protein homolog (Fragment) | 1226.66 | * |
| Q9P1Z9 | | | Coiled-coil domain-containing protein 180 | 587.08 | * |
| A0A6E1Y6F7 | | | Coiled-coil domain-containing protein 180 | 587.08 | * |
| A6NF36 | | | Coiled-coil domain-containing protein 182 | 336.65 | * |
| A0A590UKA6 | | | Coiled-coil domain-containing protein 30 | 342.59 | * |
| A0A590UK19 | | | Coiled-coil domain-containing protein 30 | 608.16 | * |
| A0A590UJC1 | | | Coiled-coil domain-containing protein 30 | 363.63 | * |
| A0A590UJA8 | | | Coiled-coil domain-containing protein 30 | 375.70 | * |
| A0A590UJ28 | | | Coiled-coil domain-containing protein 30 | 344.63 | * |
| A0A2U3TZI4 | | | Coiled-coil domain-containing protein 30 | 593.59 | * |
| Q5VVM6 | | | Coiled-coil domain-containing protein 30 | 598.06 | * |
| A0A590UK35 | | | Coiled-coil domain-containing protein 30 (Fragment) | 359.20 | * |
| A0A590UK05 | | | Coiled-coil domain-containing protein 30 (Fragment) | 325.89 | * |
| A0A590UJL6 | | | Coiled-coil domain-containing protein 30 (Fragment) | 332.49 | * |
| A0A590UJ92 | | | Coiled-coil domain-containing protein 30 (Fragment) | 600.73 | * |
| A0A590UJ77 | | | Coiled-coil domain-containing protein 30 (Fragment) | 342.59 | * |
| H3BTI0 | | | Cysteine-rich secretory protein LCCL domain-containing 2 | 2287.28 | * |
| Q9H0B8 | | | Cysteine-rich secretory protein LCCL domain-containing 2 | 2287.28 | * |
| J3QKP2 | | | Cysteine-rich secretory protein LCCL domain-containing 2 (Fragment) | 2195.04 | * |
| B5MC82 | | | D-dopachrome decarboxylase | 2052.47 | * |
| J3KQ18 | | | D-dopachrome decarboxylase | 2370.25 | * |
| P30046 | | | D-dopachrome decarboxylase | 3307.91 | * |
| H7C342 | | | D-dopachrome decarboxylase (Fragment) | 1255.44 | * |
| A6NHG4 | | | D-dopachrome decarboxylase-like protein | 2052.47 | * |
| F8W1U5 | | | DUF3456 domain-containing protein (Fragment) | 394.22 | * |
| F8VP03 | | | DUF3456 domain-containing protein (Fragment) | 880.83 | * |
| F8W031 | | | DUF3456 domain-containing protein (Fragment) | 5484.38 | * |
| H3BSA3 | | | Fanconi anemia group A protein (Fragment) | 500.62 | * |
| H3BS03 | | | Fanconi anemia group A protein (Fragment) | 487.28 | * |
| H3BNS0 | | | Fanconi anemia group A protein (Fragment) | 526.72 | * |
| A0A0B4J269 | | | G_PROTEIN_RECEP_F1_2 domain-containing protein | 4524.16 | * |
| M0QZ24 | | | Glyco_hydro38C2 domain-containing protein (Fragment) | 5817.33 | * |
| A0A0G2JNQ3 | | | Heterogeneous nuclear ribonucleoprotein C-like 2 | 2334.81 | * |
| C9J660 | | | Inactive C-alpha-formylglycine-generating enzyme 2 | 835.53 | * |
| E9PG02 | | | Inactive C-alpha-formylglycine-generating enzyme 2 | 753.19 | * |
| E9PBT8 | | | Inactive C-alpha-formylglycine-generating enzyme 2 | 1004.45 | * |
| F8WEX5 | | | Inactive C-alpha-formylglycine-generating enzyme 2 | 753.19 | * |
| J3KQJ1 | | | Inactive C-alpha-formylglycine-generating enzyme 2 | 1004.45 | * |
| F8WEV7 | | | Inactive C-alpha-formylglycine-generating enzyme 2 | 753.19 | * |
| F8WES7 | | | Inactive C-alpha-formylglycine-generating enzyme 2 | 753.19 | * |
| F8WA42 | | | Inactive C-alpha-formylglycine-generating enzyme 2 | 753.19 | * |
| Q8NBJ7 | | | Inactive C-alpha-formylglycine-generating enzyme 2 | 1004.45 | * |
| A8MXB9 | | | Inactive C-alpha-formylglycine-generating enzyme 2 | 1004.45 | * |
| A0A494C1B1 | | | Inactive C-alpha-formylglycine-generating enzyme 2 | 1004.45 | * |
| J3QT17 | | | Inactive C-alpha-formylglycine-generating enzyme 2 | 1004.45 | * |
| C9JL30 | | | Inactive C-alpha-formylglycine-generating enzyme 2 (Fragment) | 835.53 | * |
| H7C3B2 | | | Inactive C-alpha-formylglycine-generating enzyme 2 (Fragment) | 835.53 | * |
| A6NCM1 | | | IQ and AAA domain-containing protein 1-like | 323.75 | * |
| Q86V48 | | | Leucine zipper protein 1 | 1738.55 | * |
| E5RFK8 | | | Leucine zipper protein 1 (Fragment) | 1542.58 | * |
| E5RHU7 | | | Leucine zipper protein 1 (Fragment) | 1511.20 | * |
| M0R2P5 | | | PAT complex subunit Asterix (Fragment) | 865.90 | * |
| H0YIN7 | | | Peptidase_M24 domain-containing protein (Fragment) | 1855.79 | * |
| Q6S5H5 | | | POTE ankyrin domain family member G | 126.09 | * |
| Q6S545 | | | POTE ankyrin domain family member H | 136.95 | * |
| A0A087WTB9 | | | POTE ankyrin domain family member H | 136.95 | * |
| U3KQV3 | | | PPM-type phosphatase domain-containing protein (Fragment) | 983.25 | * |
| U3KQA9 | | | PPM-type phosphatase domain-containing protein (Fragment) | 983.25 | * |
| E7EW31 | | | Proline-rich basic protein 1 | 487.28 | * |
| A0A087X187 | | | Putative POTE ankyrin domain family member M | 123.75 | * |
| A6NI47 | | | Putative POTE ankyrin domain family member M | 136.95 | * |
| Q5T123 | | | SH3 domain-binding glutamic acid-rich-like protein 3 | 10921.71 | * |
| Q9H299 | | | SH3 domain-binding glutamic acid-rich-like protein 3 | 10925.86 | * |
| E5RI56 | | | Skp1_POZ domain-containing protein (Fragment) | 1224.20 | * |
| Q96K17 | | | Transcription factor BTF3 homolog | 436.46 | * |
| E5RJG6 | | | Transforming acidic coiled-coil-containing protein 1 (Fragment) | 813.43 | * |
| P37802 | | | Transgelin-2 | 10280.07 | * |
| X6RJP6 | | | Transgelin-2 (Fragment) | 10280.07 | * |
|  | | |  |  |  |

**^1^** Accession number of matched protein from Homo sapiens’ macrophages Uniprot database (https://www.uniprot.org/).

**^2^** Proteins annotation from Homo sapiens' database or by homology in NCBI database (http://www.ncbi.nlm.nih.gov/) and biological process according to the classification of KEGG (https://www.genome.jp/kegg/), UniProt database (https://www.uniprot.org/), NCBI database (http://www.ncbi.nlm.nih.gov/) and CORUM database (http://mips.helmholtz-muenchen.de/corum/).

**^3^** PLGS score is the result of different mathematical models for peptide and fragment assign prediction.

**^4^** Fold-change values were obtained by dividing the values of protein abundance (in fmol) from macrophages during infection by live PB by the abundance in the uninfected macrophages. Proteins with a minimum fold-change of 50% (≤ 0.67) were considered to be dowregulated.

**^*^** Proteins detected only in uninfected macrophages.
