# Supplementary material for: Proteomic analysis reveals changes in the proteome of human THP-1 macrophages infected with Paracoccidioides brasiliensis
Source: Front Cell Infect Microbiol. 2023 Nov 16;13:1275954. doi: 10.3389/fcimb.2023.1275954 (PMC10693345; doi:10.3389/fcimb.2023.1275954)
Supplement: Supplementary file 3 [file Table_2.docx]

**Supplementary Table S2 -** Increased proteins in macrophages co-cultured with *Paracoccidioides*

*brasiliensis* yeast cells.

| Accession number^1^ | Protein description and biological process^2^ | Score^3^ | Fold change^4^ |
| --- | --- | --- | --- |
|  | METABOLISM |  |  |
|  | **Amino acid metabolism** |  |  |
| A0A5F9ZHD5 | Adenosylmethionine decarboxylase | 389.55 | * |
| P00367 | Glutamate dehydrogenase 1_ mitochondrial | 6316.26 | 2.181 |
| P49448 | Glutamate dehydrogenase 2_ mitochondrial | 2664.99 | 2.159 |
| B4E2I4 | Glutamate--cysteine ligase | 415.59 | 2.691 |
| A0A0C4DGB2 | Glutamate--cysteine ligase | 158.26 | * |
| E1CEI4 | Glutamate--cysteine ligase | 426.91 | 3.525 |
| A0A2R8YEL6 | Glutamate--cysteine ligase (Fragment) | 415.59 | 2.691 |
| P48506 | Glutamate--cysteine ligase catalytic subunit | 426.91 | 4.220 |
| F6R5I9 | S-adenosylmethionine decarboxylase proenzyme | 248.77 | * |
| Q5VXN5 | S-adenosylmethionine decarboxylase proenzyme | 140.78 | * |
| B4DZ60 | S-adenosylmethionine decarboxylase proenzyme | 297.42 | * |
| P17707 | S-adenosylmethionine decarboxylase proenzyme | 389.55 | * |
|  | **C-compound and carbohydrate metabolism** |  |  |
| P15121 | Aldo-keto reductase family 1 member B1 | 766.63 | 2.459 |
|  | **Lipid,fatty acid and isoprenoid metabolism** |  |  |
| P18054 | Polyunsaturated fatty acid lipoxygenase ALOX12 | 336.44 | * |
| K7ENN9 | Polyunsaturated fatty acid lipoxygenase ALOX12 (Fragment) | 325.36 | * |
| Metabolism of vitamins, cofactors, and prosthetic groups | | | |
| P13716 | Delta-aminolevulinic acid dehydratase | 466.52 | * |
| B7ZBK6 | Porphobilinogen synthase (Fragment) | 445.44 | * |
|  | ENERGY |  |  |
|  | **Glycolysis and Gluconeogenesis** |  |  |
| K7ERC6 | Glucose-6-phosphate isomerase (Fragment) | 2155.99 | 4.263 |
| K7ELR7 | Glucose-6-phosphate isomerase (Fragment) | 1279.05 | 3.455 |
| A0A0J9YYI8 | Glucose-6-phosphate isomerase (Fragment) | 1279.73 | * |
| K7EIL4 | Glucose-6-phosphate isomerase (Fragment) | 1279.73 | * |
| A0A0J9YXM3 | Glucose-6-phosphate isomerase (Fragment) | 1279.73 | * |
| A0A0J9YXH9 | Glucose-6-phosphate isomerase (Fragment) | 1279.73 | * |
| A0A2R8YF08 | Glucose-6-phosphate isomerase (Fragment) | 1279.05 | 5.929 |
| Electron transport and membrane-associated energy conservation | | | |
| K7ESA0 | ATP synthase subunit alpha_ mitochondrial | 2181.35 | 2.293 |
| P25705 | ATP synthase subunit alpha_ mitochondrial | 19527.25 | 1.632 |
| K7ERX7 | ATP synthase subunit alpha_ mitochondrial (Fragment) | 18247.12 | 1.632 |
|  | CELL CYCLE AND DNA PROCESSING |  |  |
|  | **DNA processing** |  |  |
| A6NMQ1 | DNA polymerase | 502.79 | * |
| A0A5F9ZHW2 | DNA polymerase alpha catalytic subunit | 466.49 | * |
| A0A7I2V2V3 | DNA polymerase alpha catalytic subunit | 482.77 | * |
| Q9UHN1 | DNA polymerase subunit gamma-2_ mitochondrial | 174.37 | * |
| A0A5F9ZHX9 | DNA polymerase subunit gamma-2_ mitochondrial (Fragment) | 113.25 | * |
| A0A5F9ZH93 | DNA polymerase subunit gamma-2_ mitochondrial (Fragment) | 113.25 | * |
| Q96JM7 | Lethal(3)malignant brain tumor-like protein 3 | 209.43 | * |
| E9PLL7 | Lethal(3)malignant brain tumor-like protein 3 (Fragment) | 198.06 | * |
| E7EV89 | Lysine-specific demethylase 5A (Fragment) | 306.57 | * |
| D6RAY8 | SWI/SNF-related matrix-associated actin-dependent regulator of chromatin subfamily A-containing DEAD/H box 1 | 423.39 | * |
| Q9H4L7 | SWI/SNF-related matrix-associated actin-dependent regulator of chromatin subfamily A containing DEAD/H box 1 | 451.32 | * |
|  | **Cell Cycle** |  |  |
| H0YDJ8 | Torsin-1A-interacting protein 1 (Fragment) | 638.33 | * |
|  | TRANSCRIPTION |  |  |
|  | **RNA synthesis** |  |  |
| Q9HC52 | Chromobox protein homolog 8 | 266.82 | * |
| C9J6K3 | Chromobox protein homolog 8 (Fragment) | 224.73 | * |
| C9JM54 | Chromobox protein homolog 8 (Fragment) | 224.73 | * |
| Q12948 | Forkhead box protein C1 | 209.89 | * |
| Q99958 | Forkhead box protein C2 | 209.89 | * |
| P55316 | Forkhead box protein G1 | 209.89 | * |
| Q12952 | Forkhead box protein L1 | 289.47 | * |
| M0R279 | Forkhead box protein L1 (Fragment) | 217.95 | * |
| Q1PSW9 | GLI2 transcription factor (Fragment) | 330.60 | * |
| Q8N196 | Homeobox protein SIX5 | 171.05 | * |
| Q5JY17 | Nuclear receptor coactivator 5 (Fragment) | 283.89 | * |
| K7EK70 | Putative oxidoreductase GLYR1 (Fragment) | 267.23 | * |
| A0A0G2JQ71 | Putative zinc finger protein 66 | 392.42 | * |
| A0A0G2JMG9 | Putative zinc finger protein 66 | 393.12 | * |
| I3L137 | Putative zinc finger protein 66 | 392.42 | * |
| Q6ZN08 | Putative zinc finger protein 66 | 393.12 | * |
| K7EK85 | Transcription elongation factor SPT6 | 261.84 | * |
| Q7KZ85 | Transcription elongation factor SPT6 | 279.50 | * |
| J3QS64 | Transcription elongation factor SPT6 (Fragment) | 261.84 | * |
| C9J5V9 | Y-box-binding protein 1 (Fragment) | 924.49 | 12.679 |
| H0Y449 | Y-box-binding protein 1 (Fragment) | 11663.48 | 1.750 |
| P52738 | Zinc finger protein 140 | 583.45 | 40.044 |
| F5GX08 | Zinc finger protein 140 | 541.74 | 3.095 |
| F5H644 | Zinc finger protein 140 | 541.74 | 38.861 |
| F5H3Z2 | Zinc finger protein 140 | 541.74 | 38.474 |
| F5H4I1 | Zinc finger protein 140 (Fragment) | 541.74 | 39.646 |
| O43296 | Zinc finger protein 264 | 135.63 | * |
| C9K0H3 | Zinc finger protein 28 (Fragment) | 353.53 | * |
| M0R0U4 | Zinc finger protein 28 (Fragment) | 353.53 | * |
| Q9BR84 | Zinc finger protein 559 | 745.12 | 1.786 |
| A0A7I2PJA1 | Zinc finger protein GLI2 | 109.27 | 1.973 |
| P10070 | Zinc finger protein GLI2 | 117.26 | 1.973 |
| F2Z2B4 | Zinc finger protein GLI2 | 330.60 | * |
| A0A6Q8PH00 | Zinc finger protein GLI2 (Fragment) | 384.57 | * |
| H7C1U2 | Zinc finger protein GLI2 (Fragment) | 330.60 | * |
|  | **RNA processing** |  |  |
| M0QYG1 | Far upstream element-binding protein 2 (Fragment) | 169.13 | * |
| P05455 | Lupus La protein | 1286.01 | * |
| E9PFL9 | Lupus La protein | 290.03 | * |
| E7ERC4 | Lupus La protein (Fragment) | 290.03 | * |
| E9PEQ6 | Peptidyl-prolyl cis-trans isomerase | 266.79 | * |
| E9PKY5 | Peptidyl-prolyl cis-trans isomerase (Fragment) | 266.79 | * |
| Q9UNP9 | Peptidyl-prolyl cis-trans isomerase E | 266.79 | * |
| Q01130 | Serine/arginine-rich splicing factor 2 | 5925.39 | 3.781 |
| J3QL05 | Serine/arginine-rich splicing factor 2 (Fragment | 5651.92 | 3.781 |
| J3KP15 | Serine/arginine-rich splicing factor 2 (Fragment) | 5925.39 | 3.819 |
| H3BRH3 | Zinc finger CCCH domain-containing protein 18 (Fragment) | 138.00 | * |
|  | PROTEIN SYNTHESIS |  |  |
|  | **Ribosome biogenesis** |  |  |
| F8WBS5 | 60S ribosomal protein L35a | 1133.55 | * |
| F8WB72 | 60S ribosomal protein L35a | 1133.55 | * |
| P18077 | 60S ribosomal protein L35a | 1190.37 | * |
| C9K025 | 60S ribosomal protein L35a (Fragment) | 1190.37 | * |
|  | **Translation** |  |  |
| Q86U42 | Polyadenylate-binding protein 2 | 2062.02 | 1.665 |
| Q9H0W8 | Protein SMG9 | 510.06 | * |
| M0QZH1 | Protein SMG9 (Fragment) | 508.76 | * |
| M0QZC7 | Protein SMG9 (Fragment) | 508.76 | * |
| M0QYR7 | Protein SMG9 (Fragment) | 508.76 | * |
| M0QX70 | Protein SMG9 (Fragment) | 508.76 | * |
| M0R2N0 | Protein SMG9 (Fragment) | 508.76 | * |
| PROTEIN FATE ( FOLDING, MODIFICATION, DESTINATION | | | |
|  | **Protein folding and stabilization** |  |  |
| B8ZZL8 | 10 kDa heat shock protein_ mitochondrial | 26487.71 | * |
| H0Y875 | Calumenin (Fragment) | 1386.37 | 8.331 |
| A0A7P0T917 | Endoplasmin | 1649.11 | 5.640 |
| A0A7P0Z405 | Endoplasmin | 1563.03 | 9.679 |
| F8W026 | Endoplasmin (Fragment) | 1475.51 | 15.180 |
| P50502 | Hsc70-interacting protein | 5607.26 | 4.095 |
| F8WAQ7 | Hsc70-interacting protein | 163.50 | * |
| H7C3I1 | Hsc70-interacting protein (Fragment) | 5430.14 | 4.178 |
| Q16543 | Hsp90 co-chaperone Cdc37 | 260.60 | * |
| K7EL68 | Hsp90 co-chaperone Cdc37 (Fragment) | 220.82 | * |
| P07498 | Kappa-casein | 277.43 | * |
| A0A499FI48 | Protein disulfide-isomerase | 3163.72 | 1.973 |
| P13667 | Protein disulfide-isomerase A4 | 3135.13 | 1.840 |
| Q8NFI4 | Putative protein FAM10A5 | 4104.27 | 2.664 |
| O75347 | Tubulin-specific chaperone A | 4386.05 | 1.877 |
| E5RJD8 | Tubulin-specific chaperone A | 4386.05 | 1.915 |
| E5RHG6 | Tubulin-specific chaperone A | 4386.05 | 1.858 |
| E5RIW3 | Tubulin-specific chaperone A | 4109.13 | 1.822 |
|  | **Protein targeting, sorting and translocation** |  |  |
| H0YHX9 | Nascent polypeptide-associated complex subunit alpha | 2657.93 | 1.877 |
| A0A7I2V473 | Nascent polypeptide-associated complex subunit alpha | 2657.93 | 1.858 |
| E9PAV3 | Nascent polypeptide-associated complex subunit alpha_ muscle-specific form | 2697.05 | 2.225 |
| P48741 | Putative heat shock 70 kDa protein 7 | 2332.37 | 5.365 |
|  | **Protein modification** |  |  |
| A0A2R8YDX6 | Ceramide transfer protein (Fragment) | 369.71 | ***** |
| A0A7I2V5M6 | Glycylpeptide N-tetradecanoyltransferase 1 | 947.19 | ***** |
| A0A7I2V5J6 | Glycylpeptide N-tetradecanoyltransferase 1 | 947.19 | ***** |
|  | **Protein/peptide degradation** |  |  |
| A0A7I2V540 | 15S Mg(2+)-ATPase p97 subunit | 15416.04 | 2.033 |
| A0A0C4DGQ5 | Calcium-activated neutral proteinase small subunit | 228.89 | * |
| K7ELJ7 | Calcium-activated neutral proteinase small subunit | 251.27 | * |
| K7EKD8 | Calcium-activated neutral proteinase small subunit | 230.98 | * |
| K7EM73 | Calcium-activated neutral proteinase small subunit (Fragment) | 195.62 | * |
| P04632 | Calpain small subunit 1 | 251.27 | * |
| C9JH19 | Cathepsin | 12408.95 | 2.509 |
| F8WD96 | Cathepsin D | 5320.80 | 2.484 |
| A0A1B0GWE8 | Cathepsin D | 12408.95 | 2.509 |
| A0A1B0GW44 | Cathepsin D | 12261.98 | 2.509 |
| A0A1B0GVP3 | Cathepsin D | 10054.63 | 2.484 |
| A0A1B0GVD5 | Cathepsin D | 12408.95 | 2.509 |
| A0A1B0GV23 | Cathepsin D | 12408.95 | 2.509 |
| P07339 | Cathepsin D | 12408.95 | 2.509 |
| A0A0A0MSM0 | Heat shock protein 105 kDa | 1546.22 | 2.138 |
| Q92598 | Heat shock protein 105 kDa | 6471.98 | 1.506 |
| A0A1B0GU92 | Peptidase A1 domain-containing protein | 12054.32 | 2.534 |
| A0A1B0GU03 | Peptidase A1 domain-containing protein | 12054.32 | 2.509 |
| H7C469 | Peptidase A1 domain-containing protein (Fragment) | 11751.54 | 2.509 |
| H3BQC6 | Ubiquitin carboxyl-terminal hydrolase 10 (Fragment) | 262.89 | * |
|  | PROTEIN WITH BINDING FUNCTION |  |  |
|  | **Nucleic acid binding** |  |  |
| I3L155 | Major vault protein (Fragment) | 173.44 | * |
|  | **Metal binding** |  |  |
| Q02818 | Nucleobindin-1 | 1215.42 | 3.525 |
| C9J3C1 | Nucleobindin-1 (Fragment) | 399.53 | 3.896 |
| C9JBD3 | Nucleobindin-1 (Fragment) | 399.53 | 4.220 |
| A6NMY6 | Putative annexin A2-like protein | 34060.55 | 3.034 |
|  | **Nucleotide/nucleoside/nucleobase binding** |  |  |
| E9PK39 | Leucine-rich repeat serine/threonine-protein kinase 1 | 551.61 | * |
| E9PMK9 | Leucine-rich repeat serine/threonine-protein kinase 1 | 551.61 | * |
| E9PLF8 | Leucine-rich repeat serine/threonine-protein kinase 1 (Fragment) | 523.75 | * |
| CELLULAR TRANSPORT, TRANSPORT FACILITIES AND TRANSPORT ROUTES | | | |
|  | **Transport compounds (substrates)** |  |  |
| Q6NUT3 | Major facilitator superfamily domain-containing protein 12 | 389.990 | * |
| K7EQ22 | Major facilitator superfamily domain-containing protein 12 (Fragment) | 328.15 | * |
| A0A0A0MS91 | Major facilitator superfamily domain-containing protein 12 (Fragment) | 328.15 | * |
| K7EJS5 | Major facilitator superfamily domain-containing protein 12 (Fragment) | 389.77 | * |
| A0A087WU85 | Major facilitator superfamily domain-containing protein 12 (Fragment) | 328.15 | * |
| U3KQ39 | Protein transport protein Sec16B | 194.26 | * |
| Q8NBI5 | Solute carrier family 43 member 3 | 288.80 | * |
| C9J0L0 | Zinc finger CCCH domain-containing protein 11A (Fragment) | 332.03 | * |
|  | **Transport facilities** |  |  |
| A0A2R8Y615 | Chloride intracellular channel protein | 138.13 | * |
| Q9NZA1 | Chloride intracellular channel protein 5 | 199.63 | * |
| A0A5F9ZHK1 | Chloride intracellular channel protein 5 | 138.13 | * |
| A0A2R8Y4M1 | Chloride intracellular channel protein 5 | 138.13 | * |
| A0A2R8YGW2 | Chloride intracellular channel protein 5 | 138.13 | * |
| A0A2R8YF14 | H(+)/Cl(-) exchange transporter 5 (Fragment) | 570.93 | * |
| Q9UIX4 | Potassium voltage-gated channel subfamily G member 1 | 184.88 | * |
| Q01118 | Sodium channel protein type 7 subunit alpha | 202.28 | * |
|  | **Transport routes** |  |  |
| A0A0U1RQP1 | Dynamin GTPase | 139.29 | * |
| A0A0D9SFE4 | Dynamin GTPase | 139.29 | * |
| A0A0D9SFB1 | Dynamin GTPase | 139.29 | * |
| Q05193 | Dynamin-1 | 139.29 | * |
| Q9UQ16 | Dynamin-3 | 161.36 | * |
| H0YBC6 | Dynamin-3 (Fragment) | 134.35 | * |
| Q9UNF0 | Protein kinase C and casein kinase substrate in neurons protein 2 | 546.61 | * |
| A0A0U1RR22 | Protein kinase C and casein kinase substrate in neurons protein 2 (Fragment) | 533.17 | * |
| B0QYG9 | Protein kinase C and casein kinase substrate in neurons protein 2 (Fragment) | 529.81 | * |
| B0QYG8 | Protein kinase C and casein kinase substrate in neurons protein 2 (Fragment) | 533.17 | * |
| B0QYG7 | Protein kinase C and casein kinase substrate in neurons protein 2 (Fragment) | 533.17 | * |
| I6L9J0 | Protein unc-13 homolog B | 761.12 | * |
| A0A1B0GVW8 | Protein unc-13 homolog B | 780.29 | * |
| F8W8M9 | Protein unc-13 homolog B | 761.12 | * |
| A0A0U1RRB5 | Protein unc-13 homolog B (Fragment) | 742.84 | * |
| P43487 | Ran-specific GTPase-activating protein | 6502.73 | 1.954 |
| CELLULAR COMMUNICATION/SIGNAL TRANSDUCTION MECHANISM | | | |
|  | **Cellular signalling** |  |  |
| B0AZS6 | 14-3-3 protein zeta/delta | 25151.68 | 1.632 |
| B7Z2E6 | 14-3-3 protein zeta/delta | 20953.16 | 1.632 |
| E5RGE1 | 14-3-3 protein zeta/delta (Fragment) | 10269.70 | 6.685 |
| H0YB80 | 14-3-3 protein zeta/delta (Fragment) | 21222.34 | 1.616 |
| E5RIR4 | 14-3-3 protein zeta/delta (Fragment) | 10269.70 | 4.854 |
| Q01518 | Adenylyl cyclase-associated protein 1 | 24878.29 | 1.537 |
| Q5T0S3 | Adenylyl cyclase-associated protein 1 (Fragment) | 3241.67 | * |
| Q5T0R8 | Adenylyl cyclase-associated protein 1 (Fragment) | 3241.67 | * |
| Q96HY3 | CALM1 protein | 17348.89 | 1.682 |
| P0DP23 | Calmodulin-1 | 25567.46 | 1.682 |
| G3V361 | Calmodulin-1 (Fragment) | 6429.11 | 1.682 |
| E7EMB3 | Calmodulin-2 | 25532.85 | 1.682 |
| P0DP24 | Calmodulin-2 | 25567.46 | 1.682 |
| A0A590UJI2 | Calmodulin-2 (Fragment) | 6429.11 | 1.682 |
| A0A590UJC0 | Calmodulin-2 (Fragment) | 1747.72 | 2.339 |
| P0DP25 | Calmodulin-3 | 25567.46 | 1.682 |
| Q9UKG1 | DCC-interacting protein 13-alpha | 734.98 | 3.974 |
| C9JAB0 | DCC-interacting protein 13-alpha (Fragment) | 654.49 | 3.819 |
| K7EPP6 | ELKS/Rab6-interacting/CAST family member 1 (Fragment) | 117.73 | * |
| C9JGS4 | GATOR complex protein DEPDC5 | 296.38 | * |
| A0A2R8Y7U6 | GATOR complex protein DEPDC5 | 303.18 | * |
| A0A2R8Y6Y2 | GATOR complex protein DEPDC5 | 296.38 | * |
| A0A2R8Y6H8 | GATOR complex protein DEPDC5 | 299.19 | * |
| A0A2R8Y6F4 | GATOR complex protein DEPDC5 | 296.38 | * |
| A0A2R8Y5T1 | GATOR complex protein DEPDC5 | 310.41 | * |
| A0A2R8Y5P2 | GATOR complex protein DEPDC5 | 296.38 | * |
| A0A2R8YFS1 | GATOR complex protein DEPDC5 | 296.38 | * |
| A0A2R8YEW8 | GATOR complex protein DEPDC5 | 310.41 | * |
| F8WAX3 | GATOR complex protein DEPDC5 (Fragment) | 296.38 | * |
| A0A2R8Y5V3 | GATOR complex protein DEPDC5 (Fragment) | 296.38 | * |
| A0A2R8Y5E9 | GATOR complex protein DEPDC5 (Fragment) | 303.18 | * |
| A0A2R8YF97 | GATOR complex protein DEPDC5 (Fragment) | 303.18 | * |
| H7C3I3 | GATOR complex protein DEPDC5 (Fragment) | 296.38 | * |
| A0A2R8Y5U8 | Hamartin (Fragment) | 340.25 | * |
| H0Y9M0 | Inositol hexakisphosphate and diphosphoinositol-pentakisphosphate kinase 2 (Fragment) | 268.05 | * |
| O43314 | Inositol hexakisphosphate and diphosphoinositol-pentakisphosphate kinase 2 | 270.64 | * |
| A0A087WZV0 | Inositol hexakisphosphate and diphosphoinositol-pentakisphosphate kinase | 269.38 | * |
| D6RBU4 | Inositol hexakisphosphate and diphosphoinositol-pentakisphosphate kinase (Fragment) | 232.19 | * |
| Q9Y243 | RAC-gamma serine/threonine-protein kinase | 62.22 | * |
| Q8NE09 | Regulator of G-protein signaling 22 | 618.01 | 5.640 |
| J3QQX2 | Rho GDP-dissociation inhibitor 1 | 900.50 | 1.822 |
| J3KRE2 | Rho GDP-dissociation inhibitor 1 | 900.50 | 1.733 |
| P52565 | Rho GDP-dissociation inhibitor 1 | 900.50 | 1.803 |
| J3KTF8 | Rho GDP-dissociation inhibitor 1 (Fragment) | 900.50 | 1.803 |
| E5RJV2 | Serine/threonine-protein phosphatase 2A 55 kDa regulatory subunit B alpha isoform | 939.90 | * |
| E5RIY1 | Serine/threonine-protein phosphatase 2A 55 kDa regulatory subunit B alpha isoform | 942.13 | * |
| E5RHQ2 | Serine/threonine-protein phosphatase 2A 55 kDa regulatory subunit B alpha isoform | 939.90 | * |
| A0A1B0GW57 | T-lymphoma invasion and metastasis-inducing protein 1 | 268.56 | * |
| H7C079 | T-lymphoma invasion and metastasis-inducing protein 1 (Fragment) | 199.35 | * |
|  | CELL RESCUE, DEFENSE AND VIRULENCE |  |  |
|  | **Stress response** |  |  |
| A0A7I2UQ36 | Glutathione peroxidase | 110.68 | * |
| A0A2R8Y6B6 | Glutathione peroxidase 1 | 2267.20 | 2.225 |
| P07203 | Glutathione peroxidase 1 | 4743.71 | 2.225 |
| E9PFN5 | Glutathione S-transferase kappa | 843.45 | * |
| Q9Y2Q3 | Glutathione S-transferase kappa 1 | 843.45 | * |
| P09211 | Glutathione S-transferase P | 22608.57 | 1.682 |
| K7EN27 | Maillard deglycase (Fragment) | 13395.05 | 2.117 |
| K7ELW0 | Parkinson disease protein 7 | 17649.65 | 3.706 |
| P32119 | Peroxiredoxin-2 | 1915.38 | 1.896 |
|  | INTERACTION WITH THE ENVIRONMENT |  |  |
|  | **Membrane excitability** |  |  |
| O15083 | ERC protein 2 | 227.00 | * |
| H7C4G9 | ERC protein 2 (Fragment) | 148.00 | * |
|  | **Cell motility** |  |  |
| F8VRV5 | Dynein light chain | 4810.98 | 3.455 |
| F8VXL2 | Dynein light chain | 4810.98 | 3.421 |
| F8VXI7 | Dynein light chain (Fragment) | 4810.98 | 3.525 |
| P63167 | Dynein light chain 1_ cytoplasmic | 4810.98 | 3.525 |
| J3QQJ7 | Hydrocephalus-inducing protein homolog (Fragment) | 154.84 | * |
| J3QL79 | Hydrocephalus-inducing protein homolog (Fragment) | 154.84 | * |
| H0YH52 | Hydrocephalus-inducing protein homolog (Fragment) | 147.19 | * |
| H7C4W9 | Roundabout homolog 2 (Fragment) | 317.20 | * |
|  | **Cell adhesion** |  |  |
| H0YAT7 | Dystonin (Fragment) | 115.70 | * |
| SYSTEMIC INTERACTION WITH THE ENVIRONMENT | | | |
|  | **Muscle contraction** |  |  |
| P68032 | Actin_ alpha cardiac muscle 1 | 26619.22 | 1.896 |
| P62736 | Actin_ aortic smooth muscle | 26539.45 | 1.934 |
| F6QUT6 | Actin_ aortic smooth muscle (Fragment) | 18640.27 | 1.934 |
| F6UVQ4 | Actin_ aortic smooth muscle (Fragment) | 18640.27 | 1.915 |
| P63267 | Actin_ gamma-enteric smooth muscle | 26792.96 | 1.954 |
| F8WB63 | Actin_ gamma-enteric smooth muscle | 16566.76 | 1.915 |
| B8ZZJ2 | Actin_ gamma-enteric smooth muscle | 16566.76 | 1.915 |
| C9JFL5 | Actin_ gamma-enteric smooth muscle (Fragment) | 18893.79 | 1.934 |
|  | **Immune response** |  |  |
| A0A7P0TBL1 | Actin_ cytoplasmic 2 | 1198.38 | 314.190 |
| I3L3R2 | Actin_ cytoplasmic 2 (Fragment) | 18212.88 | 1.934 |
| K7EM38 | Actin_ cytoplasmic 2 (Fragment) | 18212.88 | 1.934 |
| P61160 | Actin-related protein 2 | 5478.23 | 2.013 |
| Q7Z6M3 | Allergin-1 | 326.88 | * |
| S4R3N6 | Allergin-1 (Fragment) | 322.32 | * |
| I3L414 | Caspase recruitment domain family_ member 14_ isoform CRA_d | 406.41 | 37.712 |
| E7EQR4 | Ezrin | 1969.29 | 2.293 |
| P15311 | Ezrin | 1969.29 | 2.293 |
| V9GZ37 | Heat shock 70 kDa protein 1A | 129.76 | * |
| P0DMV8 | Heat shock 70 kDa protein 1A | 2588.57 | 5.312 |
| A0A0G2JIW1 | Heat shock 70 kDa protein 1B | 2588.57 | 5.365 |
| P0DMV9 | Heat shock 70 kDa protein 1B | 2588.57 | 5.312 |
| P17066 | Heat shock 70 kDa protein 6 | 2359.95 | 196.369 |
| E9PK54 | Heat shock cognate 71 kDa protein (Fragment) | 21826.13 | 1.584 |
| E9PI65 | Heat shock cognate 71 kDa protein (Fragment) | 20719.43 | 1.568 |
| E9PQQ4 | Heat shock cognate 71 kDa protein (Fragment) | 21826.13 | 5.312 |
| E9PQK7 | Heat shock cognate 71 kDa protein (Fragment) | 21826.13 | 1.568 |
| E9PPY6 | Heat shock cognate 71 kDa protein (Fragment) | 15828.53 | 1.568 |
| E9PN25 | Heat shock cognate 71 kDa protein (Fragment) | 12588.23 | 1.584 |
| E9PM13 | Heat shock cognate 71 kDa protein (Fragment) | 9361.32 | 2.054 |
| E9PLF4 | Heat shock cognate 71 kDa protein (Fragment) | 26486.34 | 1.584 |
| Q12912 | Inositol 1_4_5-triphosphate receptor associated 2 | 504.12 | * |
| F5H006 | Inositol 1_4_5-triphosphate receptor-associated 2 | 302.19 | * |
| A0A1B0GU45 | Inositol 1_4_5-triphosphate receptor-associated 2 | 514.40 | * |
| F8W9L6 | Inositol 1_4_5-triphosphate receptor-associated 2 | 499.50 | * |
| H0YJ39 | Inositol 1_4_5-triphosphate receptor-associated 2 (Fragment) | 243.41 | * |
| P26038 | Moesin | 11172.13 | 2.534 |
| P19878 | Neutrophil cytosol factor 2 | 334.05 | * |
| B1ALB7 | Neutrophil cytosol factor 2 (Fragment) | 296.82 | * |
| Q06330 | Recombining binding protein suppressor of hairless | 338.53 | * |
| D6R927 | Recombining-binding protein suppressor of hairless | 508.77 | * |
| A0A7P0Z4N7 | Recombining-binding protein suppressor of hairless | 323.13 | * |
| A0A7P0T9W9 | Recombining-binding protein suppressor of hairless | 322.46 | * |
| D6RF98 | Recombining-binding protein suppressor of hairless | 323.13 | * |
| D6RCM1 | Recombining-binding protein suppressor of hairless | 322.46 | * |
| A0A7P0TBJ4 | Recombining-binding protein suppressor of hairless (Fragment) | 323.13 | * |
| A0A7P0T9Y9 | Recombining-binding protein suppressor of hairless (Fragment) | 323.13 | * |
| A0A7P0T947 | Recombining-binding protein suppressor of hairless (Fragment) | 323.13 | * |
| D6REC2 | Recombining-binding protein suppressor of hairless (Fragment) | 322.46 | * |
| D6RBQ8 | Recombining-binding protein suppressor of hairless (Fragment) | 322.46 | * |
| D6RB37 | Recombining-binding protein suppressor of hairless (Fragment) | 322.46 | * |
| D6RA45 | Recombining-binding protein suppressor of hairless (Fragment) | 322.46 | * |
| D6R9X3 | Recombining-binding protein suppressor of hairless (Fragment) | 322.46 | * |
| D6R9K5 | Recombining-binding protein suppressor of hairless (Fragment) | 322.46 | * |
| D6R946 | Recombining-binding protein suppressor of hairless (Fragment) | 322.46 | * |
| Q8WWQ8 | Stabilin-2 | 238.15 | * |
|  | CELL FATE |  |  |
|  | **Cell differentiation** |  |  |
| P51858 | Hepatoma-derived growth factor | 4908.80 | 1.954 |
| H0Y9Q5 | Prominin-1 | 241.86 | * |
| O43490 | Prominin-1 | 284.86 | * |
|  | **Cell death** |  |  |
| A0A0D9SFE5 | Lamin B1_ isoform CRA_a | 1902.56 | 1.599 |
| E9PBF6 | Lamin-B1 | 2328.82 | 1.584 |
| P20700 | Lamin-B1 | 2407.09 | 1.599 |
| A0A0D9SFY5 | Lamin-B1 (Fragment) | 1669.33 | 1.616 |
| A0A6Q8PFF5 | Prelamin-A/C | 1143.77 | * |
| F8WB19 | Solute carrier family 35 member F6 | 566.37 | * |
|  | BIOGENESIS OF CELLULAR COMPONENTS |  |  |
|  | **Eukaryotic plasma membrane** |  |  |
| F8WCL1 | Limb region 1 protein homolog | 363.05 | * |
| Q7Z4Q8 | Membrane progestin receptor delta | 290.57 | * |
| B4DJ42 | Membrane progestin receptor delta | 248.99 | * |
|  | **Cytoskeleton/structural proteins** |  |  |
| A6NL76 | Actin_ alpha skeletal muscle | 21029.65 | 1.934 |
| P68133 | Actin_ alpha skeletal muscle | 26574.99 | 1.896 |
| A0A6Q8PH58 | Actin_ cytoplasmic 1 | 18212.88 | 1.915 |
| A0A6Q8PGD7 | Actin_ cytoplasmic 1 | 252.80 | 314.190 |
| C9JZR7 | Actin_ cytoplasmic 1 (Fragment) | 16393.01 | 1.915 |
| C9JUM1 | Actin_ cytoplasmic 1 (Fragment) | 16393.01 | 1.934 |
| C9JTX5 | Actin_ cytoplasmic 1 (Fragment) | 16313.25 | 1.934 |
| A0A2R8YGF8 | Actin_ cytoplasmic 1 (Fragment) | 18212.88 | 1.915 |
| F5GXS2 | Actinin_ alpha 4_ isoform CRA_a | 9149.45 | 1.896 |
| H7C144 | Alpha-actinin-4 | 9002.50 | 1.877 |
| K7EP19 | Alpha-actinin-4 (Fragment) | 3015.85 | 4.758 |
| F8WE39 | ARPC4-TTLL3 readthrough | 978.29 | * |
| E5RGX5 | Stathmin | 1093.45 | * |
| Q93045 | Stathmin-2 | 1093.45 | * |
| P62328 | Thymosin beta-4 | 5068.06 | 2.801 |
| Q5VU61 | Tropomyosin alpha-3 chain | 6622.03 | * |
|  | UNCLASSIFIED |  |  |
| A0A494C151 | Ankyrin repeat domain-containing protein 20A1 (Fragment) | 189.26 | 2.459 |
| Q3KPI0 | Carcinoembryonic antigen-related cell adhesion molecule 21 | 249.72 | * |
| A0A0B4J1W4 | Carcinoembryonic antigen-related cell adhesion molecule 21 | 249.72 | * |
| A0A0G2JSC8 | Carcinoembryonic antigen-related cell adhesion molecule 21 | 249.72 | * |
| A0A590UJ10 | Obscurin | 308.43 | * |
| A0A590UK18 | Obscurin (Fragment) | 292.06 | * |
| A0A590UJD5 | Obscurin (Fragment) | 292.06 | * |
| A5A3E0 | POTE ankyrin domain family member F | 14830.97 | 1.716 |
| J3KRF4 | Proline-rich protein 29 | 375.05 | * |
| H0YBZ6 | Protein FAM184A (Fragment) | 191.02 | * |
| H0YBE3 | Protein FAM184A (Fragment) | 191.02 | * |
| A0A0G2JLP6 | Sperm-associated antigen 11B | 271.20 | * |
|  |  |  |  |

**^1^** Accession number of matched protein from Homo sapiens’ macrophages Uniprot database (https://www.uniprot.org/).

**^2^** Proteins annotation from Homo sapiens' database or by homology in NCBI database (http://www.ncbi.nlm.nih.gov/) and biological process according to the classification of KEGG (https://www.genome.jp/kegg/), UniProt database (https://www.uniprot.org/), NCBI database (http://www.ncbi.nlm.nih.gov/) and CORUM database (http://mips.helmholtz-muenchen.de/corum/).

**^3^** PLGS score is the result of different mathematical models for peptide and fragment assign prediction.

**^4^** Fold-change values were obtained by dividing the values of protein abundance (in fmol) from macrophages during infection by live PB by the abundance in the uninfected macrophages. Proteins with a minimum fold-change of 50% (≥ 1.5) were considered to be upregulated.

**^*^** Proteins detected only in infected macrophages.
